# Supplementary material for: Navigating alcohol’s impact: A mixed-methods analysis of community perceptions and consequences in Northern Tanzania
Source: PLoS One. 2025 Sep 17;20(9):e0331490. doi: 10.1371/journal.pone.0331490 (PMC12443300; doi:10.1371/journal.pone.0331490)
Supplement: Attachment — Submitted filename: PONE-D-23-33073_R1 with Track Change.pdf [file pone.0331490.s007.pdf]

PLOS ONE

Understanding the Impacts and Perceptions of Alcohol Use in Northern Tanzania: A Mixed-Methods Analysis

--Manuscript Draft--

**Manuscript Number:**	PONE-D-23-33073R1
**Article Type:**	Research Article
**Full Title:**	Understanding the Impacts and Perceptions of Alcohol Use in Northern Tanzania: A Mixed-Methods Analysis
**Short Title:**	Understanding the Impacts and Perceptions of Alcohol Use in Northern Tanzania
**Corresponding Author:**	Catherine A. Staton, M.D., MScGH<br>Duke University<br>Durham, UNITED STATES
**Keywords:**	Alcohol; emergency medicine; women; Tanzania; consequences; community perceptions
**Abstract:**	Background: Worldwide, alcohol is a leading risk factor for death and disability. Tanzania has particularly high rates of consumption and few resources dedicated to minimizing alcohol-related harm. Ongoing policy efforts are hampered by dynamic sociocultural, economic, and regulatory factors contributing to alcohol consumption. Through the voices of Kilimanjaro Christian Medical Center (KCMC) patients, this study aimed to investigate community perceptions surrounding alcohol and the impact of its use in this region. Methods: This was a mixed-methods study conducted at KCMC between October 2021 and May 2022. 676 adult (≥18 years old) Kiswahili-speaking patients who presented to KCMC's Emergency Department (ED) or Reproductive Health Clinic (RHC) were enrolled through systematic random sampling to participate in quantitative surveys. Nineteen participants were selected for in-depth interviews (IDIs) through purposeful sampling. The impact and perceptions of alcohol use were measured through Drinkers' Inventory of Consequences (DrInC) scores and analyzed in RStudio using descriptive proportions. IDI responses were explored through a grounded theory approach using both inductive and deductive coding methodologies in NVivo. Results: Men attending the ED were found to have the highest average [SD] DrInC scores (16.4 [19.6]), followed by ED women (9.11 [13.1]), and RHC women patients (5.47 [9.33]), with higher scores indicating a broader array of consequences. Participants noted alcohol to have both perceived advantages and clear harms within their community. Increased conflict, long-term health outcomes, financial instability, stigma, and sexual assault were seen as negative consequences. Benefits were primarily identified for men and included upholding cultural practices, economic growth, and social unity. Physical and financial harm from alcohol impacted both genders, however, alcohol-related stigma and sexual assault were found to disproportionately affect women. Conclusion: Our findings suggest that perceptions around drinking are nuanced and alcohol's social and physical consequences differ significantly by gender. To effectively minimize local alcohol-related harm, future alcohol-focused research and policy efforts should consider the complex sociocultural role that alcohol holds in the Moshi community.
**Order of Authors:**	Alena Pauley, MScGH
	Madeline Metcalf
	Mia Buono
	Kirstin West
	Sharla Rent, MD, MScGH
	William Nkenguye
	Yvonne Sawe
	Mariana Mikindo
	Joseph Kilasara, APRN, MNS-FNP

Powered by Editorial Manager® and ProduXion Manager® from Aries Systems Corporation

	Bariki Mchome, MD, PhD
	Blandina T. Mmbaga, MD, PhD
	João Ricardo Nickenig Vissoci, MBA, MSc, PhD
	Catherine A. Staton, M.D., MScGH

Response to Reviewers:

February 9th, 2024

To Whom It May Concern:

We appreciate the helpful suggestions presented to us by our reviewers and thank them for the opportunity to improve our manuscript. We have incorporated the changes requested by both reviewers and our academic editor in our attached submission with our specific modifications outlined below. Our revised manuscript has been attached to this resubmission packet.

Reviewer #1:

Comment: Knowledge gap is not adequately described.
Response: We thank the reviewer for this comment. We have edited the final paragraph of the introduction to more clearly define the knowledge gap that we aimed to address with this research. This modification now reads, 'However, literature has yet to explore the specific societal perceptions and implications of alcohol use within the Moshi community. Understanding the full scope of alcohol's impact and why its use remains endemic is essential to mitigating its effects. This knowledge, in turn, can be used to better inform and shape future alcohol-reduction interventions.'

Comment: Study design not clearly described. The authors describe using secondary data analysis while on the other hand the authors describe the method for data collection were survey for quantitative data and in-depth interview for qualitative data. Is the author using already existing data for secondary analysis or collected the primary data during the study time?
Response: We thank the reviewer for this clarification. We have updated the study design section of our manuscript to read, 'This study used a sequential explanatory mixed-methods approach to explore community perceptions and implications of alcohol use among KCMC's Emergency Department (ED) and Reproductive Health Center (RHC) patients in Moshi, Tanzania.' In this revision, we have removed mention of a secondary analysis as primary data was collected during the study timeline and is analyzed in this manuscript.

Comment: Description regarding study sample/population not clear. The author does not explain the rationale of using female participants who attended RCH clinic while based on standard the pregnant woman or lactating mother were the mostly attending RCH clinic and are not allowed to take any kind of alcohol at that time due to pregnancy or lactation.
Response: We appreciate this feedback from the reviewer and the opportunity to improve our manuscript in addressing it. We have added greater detail around our decision to enroll RHC patients under the section 'Study Setting.' This addition reads, 'the RHC…was selected because of its primarily female patient population, which facilitated a richer, gynocentric perspective on alcohol use which has been under-represented in past local research. Measuring alcohol use in pregnant or postpartum women was also important for maternal and child health given the high rates of alcohol use during pregnancy previously described in this setting.' The data we collected on alcohol use in pregnancy will be explored in an upcoming manuscript from our team.

Comment: The inclusion and exclusion criteria is not clearly stated.
Response: We appreciate this feedback from the reviewer. We have more clearly stated our eligibility criteria in our updated section entitled 'Study Population.' This addition reads, 'Those enrolled met the following eligibility criteria: 1) fluency in Kiswahili, 2) ability to provide informed consent, 3) aged 18 or older, 4) not a prisoner, and 5) received initial care at KCMC's ED or RHC.'

Comment: The reasons for stratified sampling is not stated.
Response: We appreciate this comment from the reviewer. We have updated the

“Study Design and Sampling Technique” subsection of the methods to outline the enrollment groups and rationale in accordance with our study aims. This addition reads, ‘a systematic random sampling strategy was employed to acquire a representative sample of our three patient populations, male ED, female ED patients, and female RHC patients, and better describe the characteristics of each. Except for ED females, every third patient was approached for potential using the ED triage or RHC intake registries. To meet enrollment goals given that significantly fewer women seek care at the ED than men, every eligible female ED patient was approached for study participation’

Comment: Data collection is not clear. The reason of using systematic random sampling for male participants attending at Emergency Department and Female participant attending at RCH clinic only while including every female participant attending ED not stated
Response: We appreciate this feedback from the reviewer and have updated the relevant section as such. Under the same section specified in our comment above, our manuscript now reads: ‘Except for ED females, every third patient was approached for potential study participation using the ED triage or RHC intake registries. For women seeking ED care, every eligible individual was approached for study participation. This was done as significantly fewer women seek care at the ED and more women were needed in this particular study to accurately determine the prevalence of AUD.’

Comment: It is also not stated how the data analysis would be adjusted to accommodate the stratification
Response: We thank the reviewer for this comment. Given that our study sought to describe alcohol-related consequences and perceptions primarily as they relate to gender and clinical unit, data stratification was not performed. Instead, descriptive statistics were used in this analysis with the intention of understanding these populations holistically.

Comment: Data analysis for qualitative data is not clearly stated. The author describes using thematic analysis and grounded theory. Which method is used in analyzing qualitative data?
Response: We appreciate the reviewer for bringing this gap in our methods to our attention. We have heavily revised our qualitative data analysis section to list a grounded theory methodology as well as provided a more in-depth discussion of the exact analysis methods employed. This modification can be seen in our ‘Data Analysis Procedure’ subsection of our ‘Qualitative Data’ methodology.

Comment: The reasons for using grounded theory is not stated, also, it is not utilized either.
Response: We appreciate this comment from the reviewer. We have made substantial changes to our qualitative data analysis section to better describe why this methodology was chosen and how it was utilized in our analysis process.

Comment: To report if the questionnaire has been tested for internal validity not stated.
Response: We appreciate this feedback from the reviewer. We have updated our “Data Collection and Instruments” subsection of our quantitative methods to incorporate the previous cultural adaptation and validation of the DrInC instrument in this study setting. We have additionally modified our “Data Collection and Instruments” subsection of our qualitative methods to read, ‘the interview guide was piloted by research team members at KCMC to ensure interview relevancy and cultural and linguistic validity prior to the commencement of the interviews.’

Comment: The author did not describe the demographic characteristics of the participants participating in quantitative survey.
Response: We appreciate this feedback. We have included an additional table containing the demographic characteristics of the entire study sample in this revised manuscript version.

Comment: Most of the quantitative data before regression analysis is not supported by statistical data
Response: We thank the reviewer for bringing this feedback. We have added a table on study population demographics to the beginning of our quantitative results section.

Comment: Data analysis in qualitative data is not sufficiently described. How does the end up with themes and sub themes without codes and emerging themes?
Response: We appreciate this important question from the reviewer. We have updated our qualitative data analysis section to better describe our analysis process. The relevant part of this addition reads, ‘US and Tanzanian study team members co-developed the initial codebook from the first several IDIs. This codebook was inductively and deductively generated, using broad categories from the interview guide to provide a general framework for qualitative themes along with organic generation of codes without preconceived notions from the study team in concordance with grounded theory methodology. The themes established within the codebook facilitated the creation of content memos for each emerging theme and sub-theme which in turn provided the basis for our qualitative findings section below. The codebook remained an evolving document as new themes emerged throughout data collection.’

Comment: The protocol is in Standard English; however, there are numerous typographical and grammatical errors.
Response: We appreciate this feedback from the reviewer. We have revised this manuscript to address grammatical errors.

Comment: I think the author needs to provide the recommendations to relevant authorities based on the findings of the study.
Response: We appreciate this suggestion from the reviewer and have added a recommendation to the relevant authorities at the conclusion of our discussion section. This addition reads, ‘we recommend that local policy and healthcare efforts acknowledge and incorporate the roles of alcohol use in future alcohol-related programming and treatment initiatives in the Moshi community.’

Reviewer #2:
Comment: L114: The line states that "Factors underlying differential expectations of use will also be explored" how was this included in this study?
Response: We thank the reviewer for bringing this incongruity to our attention. As this manuscript did not fully explore factors underlying expectations of use, we have removed this sentence from the manuscript.

Comment: L137: Were all the participants recruited involved with alcohol use (already in use of alcohol) or anyone who falls in an interval of 3 in either EMD or RCH was involved?
Response: We thank the reviewer for this question. To answer your initial inquiry, perceived or measured alcohol use was not considered when assessing individuals for enrollment eligibility. We have updated the “Study Population” section of our methods to clarify this point accordingly.

Comment: L152: The line is openly stated but I suggest it to be restated as "researcher administered questionnaire was employed so as …"
Response: We appreciate this suggestion from the reviewer. We have restated this sentence to clarify that all survey questions were delivered orally by a member of the research team.

Comment: L163: Although there are no clear cutoff points for DrInC tool, what were the cutoff points used by previous studies that could be used as reference? OR what was the reference used to determine the cutoff points used in the current study?
Response: We appreciate this question from the reviewer and acknowledge this gap in our manuscript. We have updated our section ‘Instruments and Variable Measurements’ to further explain this gap. This change now reads, ‘…while the DrInC tool has been validated in the study setting (DOI: 10.3389/fpubh.2018.00330.), no particular clinically relevant cut-off score for this tool exists, in part because this is among the first studies to apply this tool among Kiswahili-speaking patients, but also because ideally individuals would not experience any alcohol-related consequences. To help account for this lack of cut-off score, DrInC scores were correlated with alcohol use as is detailed further below.’

Comment: L202: Was there any software tool used to analyze the qualitative data?
Response: Thank you for bringing this gap in our methods section to our attention. We

Powered by Editorial Manager® and ProduXion Manager® from Aries Systems Corporation

have added to our manuscript that 'All qualitative analysis was conducted in NVivo software version 12 and approved by the entire research team prior to the reporting of these results' to the end of our qualitative data analysis procedure subsection.

Comment: The discussion part is good, however the perceived harm which is one of the qualitative results was more discussed as compared to perceived benefits. The comparison from different study could provide more information which can be used for future interventions of alcohol use disorders.
Response: We appreciate this comment from the reviewer and acknowledge our discrepancy between discussed harms versus benefits. We have expanded upon our discussion of the perceived benefits of alcohol use, particularly in explaining the effects of increased social unity and financial growth for local sellers, throughout the discussion section. However, in general, IDI participants mentioned substantially more alcohol-related harms than benefits and thus were given greater space in our discussion section overall.

Comment: Also, I would suggest adding different studies which did not concur with the results obtained from the current study so as to bring more challenges to the future researchers.
Response: We have added greater discussion and reference to other studies that challenge our conclusions. For example, one addition reads, 'While this statement [from the WHO] and our qualitative data reports no physical benefits with alcohol use, past research has indicated that moderate alcohol intake can have positive circulatory and cardiovascular effects as well as being protective against the incidence of type 2 diabetes.'

Academic Editor:

Comment: List the authors before the authors' affiliation.
Response: Thank you for the feedback. The authors are listed above their respective affiliations on page 1.

Comment: Authors' contribution has to come at the end.
Response: We appreciate this revision. The authors' contributions have been moved to page 37 after the manuscript's conclusion section.

Comment: No need of repeating the title again in page 3
Response: We appreciate this revision. The title has been deleted from page 3.

Comment: Have a heading abstract, then its sub-heading.
Response: We acknowledge this request and have updated the headings and sub-headings for the abstract.

Comment: Conclude the background of abstract with the general purpose of the study.
Response: We appreciate this suggestion from the editor. We have updated the last sentence of our background within our abstract to read, 'through the voices of Kilimanjaro Christian Medical Center (KCMC) patients, this study aimed to investigate community perceptions surrounding alcohol and the impact of its use in this region.'

Comment: In the method section have a sentence on the data collection method and tool, as well it is not clear on the data analysis method used in the study.
Response: We thank the editor for this comment and have edited the methods section of our abstract accordingly. Our last sentences now read, 'The impact and perceptions of alcohol use were measured through Drinkers' Inventory of Consequences (DrInC) sores and analyzed in RStudio using descriptive proportions. IDI responses were explored through a grounded theory approach using both inductive and deductive coding methodologies in NVivo.'

Comment: Conclusion section is not talking on the key findings obtained from the quantitative part of the study, as well in the conclusion what do you mean be ED men and ED women? These were men and women attending emergency department they cannot be called ED men and women
Response: We appreciate this comment from the editor. We have edited the discussion section to include a more thorough discussion of quantitative results and how these in

turn interact with our qualitative findings. Additionally, we have also removed "ED men" and "ED women" phrasing from these sections and replaced it with "men and women attending the ED".

Comment: In line 79, the sentence has language issue, line 112 change the sentence to past tense, this is a report.
Response: We appreciate these comments from the editor and both sentences have been updated accordingly. The first now reads, 'While hazardous alcohol consumption is a global issue, its use is increasing especially rapidly in low- and middle-income countries.' The latter sentence has been updated to '…this analysis addressed this gap by…' in accordance with your feedback.

Comment: Line 118, what do you mean by secondary analysis, did the study used secondary data? How while you have qualitative part of the study
Response: We thank the editor for this question. At the prompting of this comment and with further discussion, we confirm that this was not a secondary analysis; rather, we collected primary, cross-sectional data between October and May 2022 and analysis occurred following the completion of all data collection. This sentence now reads, 'this cross-sectional study used a sequential explanatory mixed-methods approach to explore community perceptions and implications of alcohol use among ED and RHC patients at KCMC between October 2021 and May 2022.'

Comment: What was the study design, mixed method is not a design rather an approach.
Response: We appreciate this important question and clarification. As also outlined in the comment above, we have revised our methods section to describe our study design and analytical methods more accurately, including our cross-sectional study design and sequential explanatory mixed-methods approach.

Comment: Re-arrange this section to subheadings as follows
i. Study setting-justification of the selected setting and design, which design in the mixed method was used?
ii. Study population- the inclusion and exclusion criteria
iii. Quantitative part
1. Study design
2. Sample size estimation
3. Sampling technique
4. Data collection method and instrument
5. Variables and variables measurement
6. Data analysis procedure
iv. Qualitative
1. Study design
2. Sample size estimation
3. Sampling technique
4. Data collection method and instrument
5. Data analysis procedure
This is important for clarity, in the current state the section is not clear
Response: We appreciate this format and organization revision from the editor. We have revised our methods section to include the outlined subheadings with the exceptions of 'study setting' and 'data collection method and instrument.' The former has been renamed as 'study overview and setting' as the initial paragraph in this section describes the broad study methods and reasoning for our mixed-methods approach. The latter has been added as 'Data Collection Method' to improve manuscript clarity as a high amount of overlap exists between our 'instrument' and 'variable' content.

Comment: Line 137 there is RHC, this is mentioned for the first time, please clarify.
Response: We appreciate this comment. However, RHC had previously been clarified as the Reproductive Health Center in the first sentence of our methods section, proceeding line 137. Thus, no change was made.

Comment: How did you conduct data analysis? What analysis software did you use?
Response: Thank you for these questions. Data analysis has been noted in our A statement noting that all quantitative data analysis was conducted in R Studio is listed

	on line 234. A statement has been added to the qualitative data analysis section clarifying that all qualitative analysis was conducted in NVivo software and can be found on line 292.<br><br>Comment: Describe the study respondents' characteristics (frequency distribution table)<br>Response: We appreciate this suggestion from the editor. We have included a demographics table of the entire study population in the revised version of this manuscript.<br><br>Comment: No need for table 3 after you have attended a above<br>Response: We thank the editor for this suggestion. We have removed Table 3 from the manuscript.<br><br>Comment: No discussion for quantitative results<br>Response: We appreciate this comment from the editor. We have edited the discussion section to better incorporate our quantitative findings and describe how our qualitative data contradicts or supports these data.<br><br>Comment: Why men and women in emergency department?<br>Response: We appreciate this question from the editor and have revised our methods section to make this clearer for future readers. Men and women presenting at the emergency department were enrolled in this study as previous research has suggested the higher prevalence of alcohol use among ED patients. Given that this study sought to understand the consequences of drinking itself, a patient population with above average alcohol intake was well-suited for understanding the full scope of the impacts of alcohol use. The corresponding revision can be seen in the second paragraph of our 'study overview and setting section' and is also detailed in the last response of this rebuttal letter.<br><br>Comment: Why mixed method, what did you want to achieve by combining the approaches (quantitative and qualitative), at what point did these two approaches meet?<br>Response: Thank you for this question and the opportunity for us to strengthen our manuscript in answering it. A mixed-method approach was employed to better contextualize alcohol use in describing both the quantitative prevalence of alcohol-related consequences among ED and RHC patients in Moshi, Tanzania, as well as the more nuanced community perceptions of its use. Consequently, we have added to the end of our study design section, 'this mixed-methods approach was chosen to facilitate the triangulation of data so that robust and accurate epidemiological conclusions could be drawn while also providing deeper cultural context for the complex social, emotional, and physical implications of alcohol use in this community.' Additionally, we have revised our discussion section to better explore the overlap between qualitative and quantitative data.<br><br>Comment: Why health facility study and not community-based study, what motivated you to select health facility and ED in particular?<br>Response: We appreciate this question and have updated the 'study overview and setting' section of our manuscript in line with this suggestion. To respond to this question, this update reads, 'the ED was selected as one of the two study sites to better understand risky alcohol use behaviors given the correlation between excessive alcohol use and injuries like road traffic incidents or violence that necessitate emergency care. Additionally, previous work from our team in developing alcohol-reduction interventions at KCMC's ED meant that continued research in this unit would align with programmatic goals and increase the likelihood of intervention success. The RHC, on the other hand, was chosen because of its primarily female patient population, which facilitated a richer, gynocentric perspective on alcohol use which has been under-represented in past local research. Measuring alcohol use in pregnant or postpartum women was also important for maternal and child health given the high rates of alcohol use during pregnancy previously described in this setting. As such, enrolling patients from these two clinical units allowed us to better explore the intersection of gender and hazardous drinking, the primary goal of the overarching study.'
**Additional Information:**	

Question	Response
**Financial Disclosure**<br><br>Enter a financial disclosure statement that describes the sources of funding for the work included in this submission. Review the submission guidelines for detailed requirements. View published research articles from *PLOS ONE* for specific examples.<br><br>This statement is required for submission and **will appear in the published article** if the submission is accepted. Please make sure it is accurate.<br><br>**Funded studies**<br>Enter a statement with the following details:<br>• Initials of the authors who received each award<br>• Grant numbers awarded to each author<br>• The full name of each funder<br>• URL of each funder website<br>• Did the sponsors or funders play any role in the study design, data collection and analysis, decision to publish, or preparation of the manuscript?<br><br>Did you receive funding for this work?	Yes
Please add funding details.<br>as follow-up to "**Financial Disclosure**<br><br>Enter a financial disclosure statement that describes the sources of funding for the work included in this submission. Review the submission guidelines for detailed requirements. View published research articles from *PLOS ONE* for specific examples.<br><br>This statement is required for submission and **will appear in the published article** if the submission is accepted. Please make sure it is accurate.	This project was funded by the Duke Global Health Institute Graduate Student funds (AMP), and the Josiah Trent Foundation (21-06 to CAS). These two financial awards funded the salaries of JK, YS, and MMi as research assistants hired specifically for this study. No other authors received specific funding for this work. Infrastructure built by an NIH grant (R01 AA027512 to CAS) was used to support the data collection process for this grant to understand gender-related aspects of alcohol use at KCMC. The funders had no role in study design, data collection and analysis, decision to publish, or preparation of the manuscript.

Powered by Editorial Manager® and ProduXion Manager® from Aries Systems Corporation

**Funded studies**<br>Enter a statement with the following details:<br>• Initials of the authors who received each award<br>• Grant numbers awarded to each author<br>• The full name of each funder<br>• URL of each funder website<br>• Did the sponsors or funders play any role in the study design, data collection and analysis, decision to publish, or preparation of the manuscript?<br><br>Did you receive funding for this work?"	
Please select the country of your main research funder (please select carefully as in some cases this is used in fee calculation).<br>as follow-up to "**Financial Disclosure**<br><br>Enter a financial disclosure statement that describes the sources of funding for the work included in this submission. Review the submission guidelines for detailed requirements. View published research articles from *PLOS ONE* for specific examples.<br><br>This statement is required for submission and **will appear in the published article** if the submission is accepted. Please make sure it is accurate.<br><br>**Funded studies**<br>Enter a statement with the following details:<br>• Initials of the authors who received each award<br>• Grant numbers awarded to each author<br>• The full name of each funder<br>• URL of each funder website<br>• Did the sponsors or funders play any role in the study design, data collection and analysis, decision to publish, or preparation of the manuscript?<br><br>Did you receive funding for this work?"	UNITED STATES - US
**Competing Interests**	The authors have declared that no competing interests exist.

Use the instructions below to enter a competing interest statement for this submission. On behalf of all authors, disclose any competing interests that could be perceived to bias this work—acknowledging all financial support and any other relevant financial or non-financial competing interests.<br><br>This statement is required for submission and **will appear in the published article** if the submission is accepted. Please make sure it is accurate and that any funding sources listed in your Funding Information later in the submission form are also declared in your Financial Disclosure statement.<br><br>View published research articles from *PLOS ONE* for specific examples.<br><br>**NO authors have competing interests**<br><br>Enter: *The authors have declared that no competing interests exist.*<br><br>**Authors with competing interests**<br><br>Enter competing interest details beginning with this statement:<br><br>*I have read the journal's policy and the authors of this manuscript have the following competing interests: [insert competing interests here]*<br><br>* typeset	
**Ethics Statement**<br><br>Enter an ethics statement for this submission. This statement is required if the study involved:<br><br>• Human participants<br>• Human specimens or tissue<br>• Vertebrate animals or cephalopods<br>• Vertebrate embryos or tissues	Ethical approval for this study was obtained from the Duke University Institutional Review Board, the Kilimanjaro Christian Medical University College Ethical Review Board, and the Tanzanian National Institute of Medical Research prior to any data collection. Personal health information was employed in screening and enrollment procedures, but was de-identified when collected, stored, and analyzed and shared only via a data share agreement. Confirmation of IRB approval are uploaded as supplementary documents to this submission.

Powered by Editorial Manager® and ProduXion Manager® from Aries Systems Corporation

• Field research<br><br>Write "N/A" if the submission does not require an ethics statement.<br><br>General guidance is provided below. Consult the submission guidelines for detailed instructions. **Make sure that all information entered here is included in the Methods section of the manuscript.**	

**Format for specific study types**<br><br>**Human Subject Research (involving human participants and/or tissue)**<br>• Give the name of the institutional review board or ethics committee that approved the study<br>• Include the approval number and/or a statement indicating approval of this research<br>• Indicate the form of consent obtained (written/oral) or the reason that consent was not obtained (e.g. the data were analyzed anonymously)<br><br>**Animal Research (involving vertebrate animals, embryos or tissues)**<br>• Provide the name of the Institutional Animal Care and Use Committee (IACUC) or other relevant ethics board that reviewed the study protocol, and indicate whether they approved this research or granted a formal waiver of ethical approval<br>• Include an approval number if one was obtained<br>• If the study involved *non-human primates*, add *additional details* about animal welfare and steps taken to ameliorate suffering<br>• If anesthesia, euthanasia, or any kind of animal sacrifice is part of the study, include briefly which substances and/or methods were applied<br><br>**Field Research**<br><br>Include the following details if this study involves the collection of plant, animal, or other materials from a natural setting:<br>• Field permit number<br>• Name of the institution or relevant body that granted permission	
**Data Availability**<br><br>Authors are required to make all data underlying the findings described fully available, without restriction, and from the time of publication. PLOS allows rare exceptions to address legal and ethical concerns. See the PLOS Data Policy and FAQ for detailed information.	No - some restrictions will apply

A Data Availability Statement describing where the data can be found is required at submission. Your answers to this question constitute the Data Availability Statement and **will be published in the article**, if accepted.<br><br>**Important:** Stating 'data available on request from the author' is not sufficient. If your data are only available upon request, select 'No' for the first question and explain your exceptional situation in the text box.<br><br>Do the authors confirm that all data underlying the findings described in their manuscript are fully available without restriction?	
**Describe where the data may be found in full sentences. If you are copying our sample text, replace any instances of XXX with the appropriate details.**<br><br>• If the data are **held or will be held in a public repository**, include URLs, accession numbers or DOIs. If this information will only be available after acceptance, indicate this by ticking the box below. For example: *All XXX files are available from the XXX database (accession number(s) XXX, XXX.*).<br>• If the data are all contained **within the manuscript and/or Supporting Information files**, enter the following: *All relevant data are within the manuscript and its Supporting Information files.*<br>• If neither of these applies but you are able to provide **details of access elsewhere**, with or without limitations, please do so. For example:<br><br>*Data cannot be shared publicly because of [XXX]. Data are available from the XXX Institutional Data Access / Ethics Committee (contact via XXX) for researchers who meet the criteria for access to confidential data.*<br><br>*The data underlying the results presented in the study are available from (include the name of the third party*	Data are only available upon reasonable request, as participants did not consent to public data publishing, and data transfer requires a written agreement approved by Kilimanjaro Christian Medical Centre Ethics Committee and the National Institute for Medical Research (Tanzania). Data are available upon request from Kilimanjaro Christian Medical Centre Ethics Committee and the National Institute for Medical Research (Tanzania) representative Gwamaka W. Nselela via email (gwamakawilliam14@gmail.com) for researchers who meet the criteria for access to confidential data.

and contact information or URL).<br>• This text is appropriate if the data are owned by a third party and authors do not have permission to share the data.<br><br>* typeset	
Additional data availability information:	

Manuscript Click here to access/download;Manuscript;Manuscript.docx

Understanding the Impacts and Perceptions of Alcohol Use in Northern Tanzania: A Mixed-Methods Analysis

Authors and affiliations:

Alena Pauley MScGH[1] – alena.pauley@duke.edu

Madeline Metcalf[1] – madeline.metcalf@duke.edu

Mia Buono[1] – mia.buono@duke.edu

Kirstin West[1] – kirstin.west@duke.edu

Sharla Rent MD MScGH[1,2] - Sharla.Rent@duke.edu

William Nkenguye[3,4] –williamnkenguye@gmail.com

Yvonne Sawe[3] – yvonnesawe90@gmail.com

Mariana Mikindo[3] – mirianamikindo@gmail.com

Joseph Kilasara APRN MNS-FNP[3,5] – joseph.kilasara@kcmuco.ac.tz

Bariki Mchome MD PHD[3,6] – Barikimchome@gmail.com

Blandina T. Mmbaga, MD PhD[1,3,7] – blaymt@gmail.com

João Ricardo Nickenig Vissoci MBA MSc PhD[1,8] – jnv4@duke.edu

Catherine A. Staton MD MScGH[1,8] – catherine.lynch@duke.edu

1 Duke Global Health Institute, Duke University, Durham, NC USA

2 Duke Department of Pediatrics, Duke University, Durham, NC USA

3 Kilimanjaro Christian Medical Center, Moshi, Tanzania

4 Kilimanjaro Christian Medical University College, Moshi, Tanzania

5 Department of Clinical Nursing, Kilimanjaro Christian Medical University College, Moshi, Tanzania

6 Department of Obstetrics and Gynecology, Kilimanjaro Christian Medical University College, Moshi, Tanzania

7 Kilimanjaro Clinical Research Institute, Moshi, Tanzania

8 Duke Department of Surgery, Duke University Medical Center, Durham, NC USA

Funding: This project was funded by the Duke Global Health Institute Graduate Student funds (AMP), and the Josiah Trent Foundation (21-06 to CAS). These two financial awards funded the salaries of JK, YS, and MMi as research assistants hired specifically for this study. No other authors received specific funding for this work. Infrastructure built by NIH grants (R01 AA027512 and D43TW012205 to CAS) was used to support the data collection process for this grant to understand gender-related aspects of alcohol use at KCMC. The funders had no role in study design, data collection and analysis, decision to publish, or preparation of the manuscript.

Competing interests: The authors declare no competing interests

Abstract

Background: Worldwide, alcohol is a leading risk factor for death and disability. Tanzania has particularly high rates of consumption and few resources dedicated to minimizing alcohol-related harm. Ongoing policy efforts are hampered by dynamic sociocultural, economic, and regulatory factors contributing to alcohol consumption. Through the voices of Kilimanjaro Christian Medical Center (KCMC) patients, this study aimed to investigate community perceptions surrounding alcohol and the impact of its use in this region. **Methods**: This was a mixed-methods study conducted at KCMC between October 2021 and May 2022. 676 adult (≥18 years old) Kiswahili-speaking patients who presented to KCMC's Emergency Department (ED) or Reproductive Health Clinic (RHC) were enrolled through systematic random sampling to participate in quantitative surveys. Nineteen participants were selected for in-depth interviews (IDIs) through purposeful sampling. The impact and perceptions of alcohol use were measured through Drinkers' Inventory of Consequences (DrInC) scores and analyzed in RStudio using descriptive proportions. IDI responses were explored through a grounded theory approach using both inductive and deductive coding methodologies in NVivo. **Results:** Men attending the ED were found to have the highest average [SD] DrInC scores (16.4 [19.6]), followed by ED women (9.11 [13.1]), and RHC women patients (5.47 [9.33]), with higher scores indicating a broader array of consequences. Participants noted alcohol to have both perceived advantages and clear harms within their community. Increased conflict, long-term health outcomes, financial instability, stigma, and sexual assault were seen as negative consequences. Benefits were primarily identified for men and included upholding cultural practices, economic growth, and social unity. Physical and financial harm from alcohol impacted both genders, however, alcohol-related stigma and sexual assault were found to disproportionately affect women. **Conclusion**:

Our findings suggest that perceptions around drinking are nuanced and alcohol's social and physical consequences differ significantly by gender. To effectively minimize local alcohol-related harm, future alcohol-focused research and policy efforts should consider the complex sociocultural role that alcohol holds in the Moshi community.

Introduction

Alcohol consumption has been implicated as a leading cause of harm, disability, and death worldwide [1]. The harmful use of alcohol is a growing and avoidable cause of premature disability and death, each year causing 5.1% of all disability-adjusted life years (DALYs) and 3 million deaths globally [1]. Alcohol use has been linked to over 200 adverse medical conditions, including digestive diseases, infectious diseases, cardiovascular disease, certain cancers, and injuries [2–4], and has also been associated with poor employment outcomes [5], individual and societal economic harm [4,6–8], social stigma [9,10], and sexual violence [11–13].

While hazardous alcohol consumption is a global issue, its use is increasing especially rapidly in low- and middle-income countries [14–16]. For example, alcohol use has become the leading risk factor for disease burden in many sub-Saharan African countries including Tanzania [17,18]. As low-income populations typically have fewer resources, financial savings, and poorer access to health care services, alcohol-related harm in these contexts is more devastating both physically and financially [1,19–21]. Though the harms of alcohol intake are significant, its use remains endemic and has been ingrained in cultural and social practices in populations across the world [22]. This holds true in Tanzania, where the cultural and social practices of alcohol use, such as the normalization of intake starting from a young age or use as a social currency, support larger quantities of consumption [23].

Adding greater complexity to alcohol's impact on individuals and communities, alcohol consumption has been shown to differ in populations according to cultural norms, regulations, age, and socio-economic status [24]. To address the factors influencing alcohol consumption, the World Health Organization (WHO) created the Conceptual Model of Alcohol Consumption and Health Outcomes. The model aims to decrease the morbidity and mortality that follow harmful

alcohol use patterns and demonstrates that understanding an individual country's sociocultural context is imperative to alter alcohol use behaviors at an individual and country level [25].

Moshi, located near Mount Kilimanjaro National Park in Tanzania, has seen exceptionally high rates of alcohol use, 2.5 times higher than in nearby regions [26,27]. Previous studies have shown that 60% of Moshi residents frequently consume alcohol, and 23% suffer from alcohol use disorder [28]. In this location, alcohol consumption has also been linked to social stigma and ostracization around female alcohol users [29], unsafe sexual behaviors [30,31], and an increased prevalence of injuries among both men and women [32]. While alcohol-related harms have been identified in Moshi, current literature specifying how community members view the harms and benefits of alcohol holistically is sparse.

Previous work from our team has examined alcohol-related stigma [29,33], perceptions of alcohol use [23], the prevalence of alcohol-related injuries upon Emergency Department (ED) arrival [32], and the development and implementation of interventions to reduce alcohol misuse for the ED injury patient population [34,35]. However, current literature has yet to explore the specific societal perceptions and implications of alcohol use within the Moshi community. Understanding the full scope of alcohol's impact and why its use remains endemic is essential in mitigating its effects. This knowledge can be used to better inform future alcohol-reduction interventions and research initiatives to be more effective and successful in decreasing alcohol-related harm in this setting [36]. This process of development can also serve as a model for population-specific harm reduction interventions in other settings. Thus, through the voices of patients at the Kilimanjaro Christian Medical Center (KCMC), this analysis addressed this gap by investigating the societal perceptions of alcohol along with the negative and positive ways that alcohol use impacts individuals and the broader Moshi community.

Methods

Study Overview and Setting

This cross-sectional study used a sequential explanatory mixed-methods approach to explore community perceptions and implications of alcohol use among Emergency Department (ED) and Reproductive Health Center (RHC) patients at KCMC. All data was collected between October 2021 and May 2022. This mixed-methods approach was chosen to facilitate the triangulation of data so that robust and accurate epidemiological conclusions could be drawn while also providing deeper cultural context for the complex social, emotional, and physical implications of alcohol use in this community.

KCMC, which is located near the border of Kenya and Kilimanjaro National Park, was chosen as the study setting as it is the largest referral and teaching hospital in the Kilimanjaro region, has existing research infrastructure, and is in an area known to have especially high rates of alcohol use. The ED was selected as one of the clinical study sites to better understand risky alcohol use behaviors given the correlation between excessive alcohol use and injuries like road traffic accidents and violence necessitating emergency care [32,37,38]. Additionally, previous work from our team in developing alcohol-reduction interventions at KCMC's ED meant that continued research in this unit would align with programmatic goals and increase the likelihood of intervention success [39]. The RHC, on the other hand, was chosen because of its primarily female patient population, which facilitated a richer, gynocentric perspective on alcohol use which has been under-represented in past local research. Measuring alcohol use in pregnant or postpartum women seeking gynecological care was also important for maternal and child health given the high rates of alcohol use during pregnancy previously described in this setting [40,41].

As such, enrolling patients from these two clinical units allowed us to better explore the intersection of gender and hazardous drinking, the primary goal of the overarching study.

Study Population

All enrolled patients met the following eligibility criteria: 1) fluency in Kiswahili, 2) ability to provide informed consent, 3) aged 18 or older, 4) not a prisoner, and 5) received initial care at KCMC's ED or RHC. Perceived or measured alcohol use was not considered when determining patient eligibility given that the study intended to provide a representative sample from the ED and RHC. Prior to administering the survey, all patients verbally confirmed that they had not been previously enrolled in the study. Ability to provide informed consent for participation in the study was defined as being medically stable, clinically sober from alcohol, and ability to complete the verbal survey independently. Patients who did not meet these criteria upon the initial approach were reevaluated for participation within 24 hours of arrival at KCMC or prior to discharge.

Quantitative Data

Study Design

Cross-sectional, quantitative survey data assessing demographic factors, alcohol use, and alcohol-related consequences were collected from KCMC ED and RHC patients. Preliminary quantitative analyses were used to guide our qualitative sampling strategy as will be discussed in detail further below.

Sample Size Estimation

Quantitative sample size was calculated based on the difference in average Alcohol Use Disorder Identification Test (AUDIT) scores between our RHC and ED patient populations as the prevalence of alcohol use disorder (AUD) was a primary point of interest in the broader study

[42]. Based on existing literature, we hypothesized the prevalence of AUD to be 10% among RHC women, 15% among ED women, and 30% among ED men. Thus, at 80% power and with 90% confidence, a sample size of 587 participants was calculated to sufficiently estimate the prevalence of risky drinking between all three study subgroups. Given that the expected difference in the prevalence of AUD was narrower between the two female populations than between either male or female groups, the study team planned to enroll significantly more women than men.

Preliminary data analysis revealed, however, that the prevalence of AUD was 40% in ED women and 45% in ED men, indicating that now 1,200 patients would need to be enrolled to adequately determine a difference in proportions between these two groups. While the study timeline and funding could not accommodate the newly estimated sample size of 1200 participants, with IRB approval, the study's target sample size was increased to enroll as many participants as feasible during the study timeline.

Sampling Technique

Over the course of the study timeline, 676 patients were enrolled through a systematic random sampling strategy, with 655 participants fully completing survey questionnaires. A systematic random sampling strategy was employed to acquire a representative sample of our three patient populations, male ED, female ED patients, and female RHC patients, and better describe the characteristics of each. Except for ED females, every third patient was approached for potential using the ED triage or RHC intake registries. For women seeking ED care, every eligible individual was approached for study participation. This was done as significantly fewer women seek care at the ED than men and more women were needed in this particular study to accurately determine the prevalence of AUD.

Data Collection Method

KCMC ED and RHC patients were approached for participation in this study by a same-gender, Kiswahili-speaking, Tanzanian research assistant trained in good clinical practices and specific study procedures. After the research assistants ensured patients were medically stable, they approached patients in a quiet, private location and introduced the study. All patients had the opportunity to decline participation, but if willing to proceed, an in-depth discussion of the risks and benefits of study participation was provided. If agreeing, written consent was obtained, and survey questions were delivered. All questions were administered orally by a research team member to encourage participation from all literacy levels.

Instruments and Variable Measurements

The DrInC was used to quantitatively assess the impact alcohol use had on an individual. DrInC, which had been cross-culturally adapted and clinically tested previously at KCMC [43], is a 50-item survey tool consisting of yes or no questions designed to assess alcohol-related consequences across five different domains [44,45]. These include physical, social responsibility, interpersonal, intrapersonal, and impulse control (Table 1). DrInC scores range from 0 to 50, with higher scores overall indicating more affirmative responses to various alcohol-related items as shown in Table 1 below. Of note, while DrInC helps to quantitatively illustrate the range of alcohol-related consequences one has experienced, it does not measure the severity or frequency of these consequences. Further, while the DrInC tool has been validated in the study setting [43], no clinically relevant cut-off score for this tool exists, in part because this is among the first studies to apply this tool among Kiswahili-speaking patients, but also because ideally individuals would not experience any alcohol-related consequences. To help account for this lack of cut-off score, DrInC scores were correlated with alcohol use as is detailed further below.

Table 1: Consequence Domains and Corresponding Items of the Drinkers' Inventory of Consequences

DrInC Subcategories	Corresponding Items
Physical	"I have had a hangover after drinking"<br>"After drinking, I have had trouble with sleeping, staying asleep, or nightmares"<br>"I have been sick and vomited after drinking"<br>"Because of my drinking, I have not eaten properly"<br>"My physical health has been harmed by my drinking"<br>"My physical appearance has been harmed by my drinking"<br>"My sex life has suffered because of my drinking"<br>"While drinking or intoxicated, I have been physically hurt, injured, or burned"
Social Responsibility	"I have missed days of work or school because of my drinking"<br>"The quality of my work has suffered because of my drinking"<br>"I have failed to do what is expected of me because of my drinking"<br>"I have gotten into trouble because of drinking"<br>"I have had money problems because of my drinking"<br>"I have spent too much or lost a lot of money because of my drinking"<br>"I have been suspended/fired from or left a job or school because of my drinking"
Interpersonal	"My family or friends have worried or complained about my drinking"<br>"My ability to be a good parent has been harmed by my drinking"<br>"While drinking, I have said or done embarrassing things"<br>"While drinking, I have said harsh or cruel things to someone"<br>"My marriage or love relationship has been harmed by my drinking"<br>"My family has been hurt by my drinking"<br>"A friendship or close relationship has been damaged by my drinking"<br>"My drinking has damaged my social life, popularity, or reputation"<br>"I have lost a marriage or a close love relationship because of my drinking"<br>"I have lost a friend because of my drinking"
Intrapersonal	"I have felt bad about myself because of my drinking"<br>"I have been unhappy because of my drinking"<br>"I have felt guilty or ashamed because of my drinking"<br>"When drinking, my personality has changed for the worse"<br>"I have lost interest in activities and hobbies because of my drinking"<br>"My spiritual or moral life has been harmed by my drinking"<br>"Because of my drinking, I have not had the kind of life that I want"<br>"My drinking has gotten in the way of my growth as a person"
Impulse Control	"I have driven a motor vehicle after having three or more drinks"<br>"My drinking has caused me to use other drugs more"<br>"I have taken foolish risks when I have been drinking"<br>"When drinking, I have done impulsive things that I regretted later"<br>"I have gotten into a physical fight while drinking"<br>"I have smoked more when I am drinking"<br>"I have been overweight because of my drinking"<br>"I have been arrested for driving under the influence of alcohol"

	"I have had trouble with the law (other than driving while intoxicated) because of my drinking"<br>"I have had an accident while drinking or intoxicated"<br>"While drinking or intoxicated, I have injured someone else"<br>"I have broken things or damaged property while drinking or intoxicated"

Data Analysis Procedure

Overall and category specific DrInC scores for the three patient populations were assessed through descriptive frequencies and proportions. DrInC scores were also analyzed in relation to patients' alcohol intake to identify if an association between increased alcohol consumption and more consequences existed. As previously referenced, alcohol consumption was measured by AUDIT, a clinical tool used to identify patients with unhealthy drinking behaviors that was administered at the same time as the DrInC survey. AUDIT scores range from 0 to 40, with higher scores representing more hazardous alcohol consumption and scores of 8 or greater clinically indicative of risk for adverse alcohol-related health effects both globally and in the study setting [46–49]. The relationship between patient DrInC and AUDIT scores was measured through a linear regression analysis, the independent variable being DrInC scores and the dependent variable being AUDIT scores. Incomplete surveys were still included in the analyses. All quantitative data were analyzed in R Studio version 1.4.

Qualitative Data

Study Design

Using a purposive sampling strategy guided by preliminary quantitative data analyses, 19 individuals who had previously been enrolled in this study and completed all study questionnaires were contacted either at the end of their survey or later by phone call to gauge their interest in participating in a semi-structured IDI. All contacted patients agreed to

participate. IDIs were conducted at KCMC and later analyzed using a grounded theory methodology.

Sample Size Estimation

All participants who completed the quantitative survey were tentatively eligible for enrollment in the semi-structured interview. The study team aimed to enroll 20 IDI participants or until data saturation had been met [50]. To maintain a representative sample of IDI participants, we aimed to enroll ten ED men, five ED women, and five RHC women for participation in the interview. For the purposes of this study, data saturation was defined as absence of new themes after three consecutive interviews for each subgroup (ED men, ED women, RHC women). IDI data collection was concluded at 19 interviews after data saturation had been met for the ED men IDI subgroup after 9 interviews.

Sampling Technique

Nineteen out of the 676 patients enrolled were selected to participate in IDIs. Participants were chosen through purposive sampling to be balanced by gender, encompass diverse demographic backgrounds in terms of age, religion, income, profession, and marital status, and hold varying degrees of personal alcohol consumption. Additionally, the research team strove to have multiple unique experiences with alcohol use represented in the final data set, from patients who only reported neutral or positive past experiences with alcohol, used to drink but are now abstinent, have a close friend or relative that drinks heavily, or suffered significant negative consequences from alcohol. Interviewee characteristics were re-reviewed after two subsequent IDIs, and any needed changes were implemented when selecting the next IDI candidate.

Data Collection and Instruments

Patients were approached for IDI participation at the conclusion of their initial survey or in a later phone call. If the patient expressed interest in participating, the same-gendered research assistant who had originally enrolled them set up a time to meet and conducted the interview with them in a private room at KCMC. All interviews were conducted in Kiswahili using a semi-structured interview guide featuring open-ended questions and probes for situations where the information provided was unclear, conflicted with previous statements, or warranted further explanation. The interview guide was piloted by research team members at KCMC to ensure interview relevancy and cultural and linguistic validity prior to the commencement of the interviews. With the consent of the participant, all IDIs were audio recorded using a handheld recording device for later transcription and translation. Lastly, IDI participants were given a small stipend (5000 TSH, equivalent to approximately $2 USD) to reimburse their travels and were offered a snack and drink midway through.

Data Analysis Procedure

A grounded theory approach, chosen as this topic remains poorly understood in the local context, was used to analyze all qualitative data [51-53]. US and Tanzanian study team members co-developed the initial codebook from the first several IDIs. This codebook was inductively and deductively generated, using broad categories from the interview guide to provide a general framework for qualitative themes along with organic generation of codes without preconceived notions from the study team in concordance with grounded theory methodology [52-54]. The themes established within the codebook facilitated the creation of content memos for each emerging theme and sub-theme which in turn provided the basis for our qualitative findings

section below. The codebook remained an evolving document as new themes emerged throughout data collection.

Interviews were coded by both Tanzanian and US research team who first independently coded initial interviews and then met to discuss and resolve any coding discrepancies. This process was repeated until 80% agreement was reached. At that point, with approval from the research team, the lead analyst completed the coding of all remaining interviews, updating the codebook accordingly. After the preliminary quantitative analyses, qualitative and quantitative data collection occurred in parallel for the remainder of the study period. All qualitative analysis was conducted in NVivo software version 12 and approved by the entire research team prior to the reporting of these results.

Ethics Statement

Ethical approval for this study was obtained from the Duke University Institutional Review Board, the Kilimanjaro Christian Medical University College Ethical Review Board, and the Tanzanian National Institute of Medical Research prior to any data collection. Personal health information was employed in screening and enrollment procedures, but was de-identified when collected, stored, and analyzed and shared only via a data share agreement.

Results

Quantitative

Six-hundred and seventy-six patients started, and 655 patients completed the survey questionnaire. The average age of all ED and RHC participants was 41.7 years old (Table 2). 80% of all participants identified with the Christian religion, 50% reported being married, and 57% reported being employed at the time of survey ~~delivery~~. Additional reporting of stratified participant demographics can be found in Table 2. Men attending the ED were found to spend the most money on alcohol per week, drink the most frequently and in the largest quantities, and have the highest average AUDIT scores as compared to women ED and RHC patients [42]. In comparing alcohol consumption with alcohol-related consequences, AUDIT and DrInC scores were found to be linearly associated across all three patient populations (Figure 1). This association was strongest for women attending the ED ($R^2 = 0.76$; $p<0.001$) and weakest for men ED patients ($R^2 = 0.65$; $p<0.001$) and women RHC patients ($R^2 = 0.53$; $p<0.001$).

For alcohol-related consequences, men attending the ED were found to have the highest average [SD] DrInC scores (16.4 [19.6]), followed by ED women (9.11 [13.1]), and RHC women (5.47 [9.33]) (Table 2). This held true for DrInC subscores, where across every category except interpersonal, ED men had the highest, ED women had the second highest, and RHC women had the lowest average DrInC scores (Table 3; Figure 2). In the interpersonal subscore, the difference in average DrInC scores was not statistically significant. However, in all subcategories except social responsibility and impulse control, ED women had the highest range of DrInC scores (Figure 2).

Table 2: Study Population Demographics

Demographics by Population Type	Overall, N = 655[1]	ED Women, N = 271[1]	RHC Women, N = 270[1]	ED Men, N = 114[1]	P Value
**Age Category**					<0.001
18 to 24	110 (18%)	47 (19%)	53 (21%)	10 (9.5%)	
25 to 34	159 (26%)	41 (16%)	92 (37%)	26 (25%)	
35 to 44	106 (17%)	42 (17%)	45 (18%)	19 (18%)	
44 to 54	94 (16%)	47 (19%)	32 (13%)	15 (14%)	
Over 55	137 (23%)	75 (30%)	27 (11%)	35 (33%)	
Missing/refused per block	49 / 655	19 / 271	21 / 270	9 / 114	
**Average Age**	41.7 (18.7)	46.1 (22.8)	36.4 (12.7)	45.2 (16.4)	0.101
**Personal Income Category (TZS per month)**					0.001
0 to 50,000	205 (32%)	110 (41%)	68 (25%)	27 (25%)	
50,001 to 100,000	44 (6.8%	17 (6.3%)	15 (5.6%)	12 (11%)	
100,001 to 150,000	56 (8.7%)	17 (6.3%)	29 (11%)	10 (9.4%)	
150,001 to 200,000	91 (14%)	34 (13%)	37 (14%)	20 (19%)	
> 200,000	248 (39%)	92 (34%)	119 (44%)	37 (35%)	
Missing/refused per block	11 / 655	1 / 271	2 / 270	8 / 114	
**Religion**					0.187
None	11 (1.7%)	5 (1.8%)	3 (1.1%)	3 (2.6%)	

Demographics by Population Type	Overall, N = 655[1]	ED Women, N = 271[1]	RHC Women, N = 270[1]	ED Men, N = 114[1]	P Value
Christian	522 (80%)	218 (80%)	222 (82%)	82 (72%)	
Muslim	121 (18%)	47 (17%)	45 (17%)	29 (25%)	
Other	1 (0.2%)	1 (0.4%)	0 (0%)	0 (0%)	
Missing/refused per block	0 / 655	0 / 271	0 / 270	0 / 114	
**Highest Educational Attainment**					<0.001
None	52 (8.7%)	30 (12%)	7 (2.9%)	15 (13%)	
Primary	182 (31%)	82 (34%)	61 (25%)	39 (35%)	
Secondary	133 (22%)	48 (20%)	64 (27%)	21 (19%)	
College	170 (29%)	65 (27%)	87 (36%)	18 (16%)	
Graduate	13 (2.2%)	4 (1.7%)	2 (0.8%)	7 (6.2%)	
Vocational	45 (7.6%)	13 (5.4%)	20 (8.3%)	12 (11%)	
Missing/refused	60 / 655	29 / 271	29 / 270	2 / 112	
**Marital Status**					0.013
Never Married or Single	135 (21%)	55 (20%)	56 (21%)	24 (21%)	
Living with a partner, not in a registered marriage	79 (12%)	26 (9.6%)	41 (15%)	12 (11%)	
Living with a partner in a registered marriage	327 (50%)	128 (47%)	140 (52%)	59 (52%)	

Demographics by Population Type	Overall, N = 655[1]	ED Women, N = 271[1]	RHC Women, N = 270[1]	ED Men, N = 114[1]	P Value
Divorced or Separated	3 (4.7%)	16 (5.9%)	6 (2.2%)	9 (8.0%)	
Widowed	81 (12%)	46 (17%)	27 (10%)	8 (7.1%)	
Missing/refused	2 / 655	0 / 271	0 / 270	2 / 114	
**Employment Status**					<0.001
Employed	371 (57%)	127 (47%)	192 (71%)	52 (46%)	
Unemployed	215 (33%)	111 (41%)	50 (19%)	54 (47%)	
Student	69 (11%)	33 (12%)	28 (10%)	8 (7.0%)	
Missing/refused	0 / 655	0 / 271	0 / 270	0 / 114	
**Tribe**					0.070
Chagga	329 (50%)	126 (46%)	146 (54%)	57 (50%)	
Iraq	21 (3.2%)	9 (3.3%)	8 (3.0%)	4 (3.5%)	
Maasai	25 (3.8%)	13 (4.8%)	7 (2.6%)	5 (4.4%)	
Mmeru	29 (4.4%)	14 (5.2%)	11 (4.1%)	4 (3.5%)	
Muha or Non-African	6 (1.0%)	3 (1.1%)	3 (1.1%)	0 (0%)	
Nyaturu	17 (2.6%)	7 (2.6%)	10 (3.7%)	0 (0%)	
Pare	70 (11%)	27 (10.0%)	25 (9.3%)	18 (16%)	
Sambaa	26 (4.0%)	7 (2.6%)	10 (3.7%)	9 (7.9%)	
Sukuma	34 (5.2%)	10 (3.7%)	16 (5.9%)	8 (7.0%)	

Demographics by Population Type	Overall, N = 655[1]	ED Women, N = 271[1]	RHC Women, N = 270[1]	ED Men, N = 114[1]	P Value
Other African	98 (15%)	55 (20%)	34 (13%)	9 (7.9%)	
Missing/refused	0 / 655	0 / 271	0 / 270	0 / 114	

[1]n / N (%); Mean (SD)

Figure 1: Association of AUDIT and DrInC Scores across Patient Populations

Table 3: Reported Average DrInC Scores and Subscores across Patient Populations

	**RHC Women (n =270)**	**ED Women (n = 271)**	**ED Men (n = 114)**	**Total Population (n = 655)**
**Overall DrInC Score,** *missing: 17*	5.47 (9.33, 75)	9.11 (13.1, 80)	16.4 (19.6, 88)	9.00 (13.9,)
**Physical Subscore,** *missing: 17*	0.60 (1.73, 12)	1.39 (2.75, 15)	2.54 (3.37, 12)	1.28 (2.62, 15)
**Intrapersonal Subscore,** *missing: 17*	0.54 (1.87, 14)	1.00 (2.34, 20)	2.86 (3.97, 17)	1.15 (2.68, 20)
**Social Responsibility Subscore,** *missing: 17*	0.52 (1.74, 13)	1.15 (2.53, 14)	2.53 (3.77, 17)	1.15 (2.66, 17)
**Interpersonal Subscore,** *missing: 17*	0.39 (1.51, 14)	0.99 (2.67, 23)	3.02 (4.42, 17)	1.12 (2.91, 23)
**Impulse Control Subscore,** *missing: 17*	0.34 (1.35, 17)	0.70 (1.89, 13)	1.99 (3.34, 17)	0.80 (2.17, 17)
Data are presented as mean (SD, range) for quantitative variables, and count (%) for qualitative variables.				

Figure 2: Box-Plot Distribution of DrInC Scores and Subscores across Patient Populations

Qualitative

Nineteen individuals (RHC women, n = 5; ED women, n = 5; ED men, n = 9) participated in IDIs. The ages of IDI participants spanned from 20 to 70 years, with a mix of education, income, and alcohol intake levels represented. Approximately a third of participants held positive views or remarked that they generally benefited from alcohol, almost half said these views and experiences were overall negative, and the remaining quarter of participants stated that it depended. Two main themes and several sub-themes related to community perceptions and the impact of alcohol emerged from the IDIs. The two main themes were Alcohol's perceived harms and Alcohol's perceived benefits (Table 4). Participants noted the ways they felt alcohol harmed either them or their community, which included a failure to fulfill their responsibilities, stigma, sexual assault, increased conflict, chronic health issues, and financial instability. On the other hand, examples of how alcohol was viewed as beneficial within the community were mentioned, including fostering economic growth, creating an outlet to uphold cultural traditions, and instilling more social unity.

Table 4: Qualitative Themes and Sub-Themes

**Themes**	**Sub-Themes**
Alcohol’s Perceived Harms	Inability to Fulfill Familial Responsibilities<br>Stigma and Discrimination<br>Sexual Consequences<br>Interpersonal Conflict and Violence<br>Injuries and Chronic Disease<br>Financial Instability
Alcohol’s Perceived Benefits	Economic Growth<br>Upholding Cultural Practices<br>Social Unity

To preface this data, the communities' perceptions and expectations of alcohol use explored in this results section is the amalgamation of general themes and trends from 19 participants. While these findings have been grouped for clarity, it is important to note that there was a significant amount of variability in responses and opinions, especially across gender, "*various religions, clans, and tribes" (IDI #3, Female)*. As one participant explains:

> *"The community is the combination of many people with different ideologies about life; there is no way that my perception will be the same with everybody in the community. It's because we have been born and raised in different families and environments and everyone came up with his/her own life basics, so according to how one came across alcohol it is how the perception will be built." (IDI #19, Female)*

Alcohol's Perceived Harms

As part of their interview, IDI participants were asked how alcohol use negatively impacted their community. Respondents stated that alcohol leads to stigma, sexual violence, risky sexual behaviors, and an inability of community members to uphold individual responsibilities. Most also saw it as an instigator of physical, verbal, and emotional conflict among family members, and believed it contributed to long-term health issues and financial instability.

> *"What I can say is the society do not have positive expectation about alcohol use, because we see families disintegrated, people fall sick as a result of alcohol and many people at first drink but are able to work and carry on their daily activities and routine but later on they fail to carry on tasks because alcohol makes them weak." (IDI #3, Female)*

Inability to Fulfill Family Responsibilities

Most participants mentioned that alcohol was an inhibitor in one's ability to fulfill their responsibilities to their family. Those who drank in excess would "*forget [their] responsibilities*" (IDI #11, Male), become too "*weak*" (IDI #3, Female), "*lazy*" (IDI #19, Female), or simply, "*fail to perform their daily duties*" (IDI #6, Female). One participant elaborates on their own experience:

> *"I see alcohol is not such a good thing…It can make one forgetting everything about his/her family, even children can go to bed hungry without you having an idea because you're busy drinking. Children left home by drunkard parents can even experience assaults of different kinds just because the parents are not there to protect them…I advice my fellow parents and those who have families to care for to stop taking alcohol"* (IDI #7, Female)

<u>Stigma and Discrimination</u>

All interviewees noted alcohol-related stigma to be present to some extent in their communities, but to varying degrees and concentrated in certain circumstances. One participant reported that they "*haven't seen any stigma among men or women…unless if after drinking you cause problems like fighting or abusing other people*" (IDI#12, Male). As another explains, stigma could also arise when one fails to accomplish preset responsibilities or drinks unsafe alcoholic beverages:

> "*Alcohol intake in Tanzania is like a ritual. Anyone can drink. The stigma arises when someone fails to control their drinking habit, like when someone drinks all the time and fails to fulfill his or her obligations, or if someone drinks those homemade beers (local brews) that are not tested and not standardized by the bureau of standards. People who drink Gongo and Dadii are really stigmatized in the community. Because once you drink*

> *those, you may even fail to control your bowels, which leads to shame and embarrassment*" (IDI #2, Male).

In addition to the quality of liquor, another notable facet of alcohol-related stigma is its interaction with gender. Participants noted that "*women who drink are stigmatized more than men*" (IDI #7, Female) and that their "*community strongly hate women who drinks alcohol"* (IDI #19, Male). This arose in part because of gender roles, where because "*women stay with the children most of the time"* (IDI #19, Male) there is concern that their alcohol habits will have greater negative repercussions on their children.

<u>Sexual Consequences</u>

Interviewees reported that alcohol use caused or contributed to significant sexual consequences uniquely experienced by each gender. The first of these was the increased risk of acquiring a sexually transmitted disease – *"when you drink too much it can put you a risky of even practice unsafe sexual intercourse"* (IDI #4, Male), which generally stemmed from having more sexual partners:

> *"Men who drink lacks discipline, they don't respect themselves or their wives, they engage in sexual immorality without considering the fact that they are aged, married and with families."* (IDI #7, Female)

A couple of men and women participants noted that alcohol could hinder their ability to perform sexual acts –

> *"A person cannot perform sexual activity well as before, to men they may encounter erectile dysfunction and women may also not be able to involve and perform sexual activity properly. Alcohol can make a person really desire the sexual intimacy but just not able to perform it because of the effect of alcohol in the brain."* (IDI #16, Female)

This, though, was only mentioned by a few participants and stands in sharp contrast to the common theme of alcohol-related sexual assault. This final sexual consequence was described almost exclusively as men being violent against women – "*there is someone I know she was invited to a man house to drink alcohol it was her first time to drink alcohol she got drunk the man raped her*" (IDI #9, Female). Participants disagreed on whether or not men incurred sexual assault when drinking, one saying *"men…cannot experience sexual violence because it is not a common practice in Tanzania"* (IDI #2, Male), while another reported that *"men…when they are drunk, they have been sexually abused by the fellow men"* (IDI #13, Female).

<u>Interpersonal Conflict and Violence</u>

Sexual assault was not the only product of the increased violent and aggressive behavior that alcohol use incited. Interpersonal conflict and violence were themes noted by three-quarters of participants. IDI #19 states,

> "*Alcohol causes misunderstandings and fights in society, I have witnessed many times that people get drunk and start to say abusive words, become very aggressive and arrogant.*" (IDI #19, Female)

Respondents described how alcohol use instigates emotional and physical conflict and distress within the family – "*I know many families that when the father is drunk, the mother and children become so anxious and afraid to sit with him closely. The most common violence is physical and emotional abuse*" (IDI #17, Male). As seen in this and the next quote, aggression was described as being more common in men – *"it's very difficult to see women fighting after drinking. But men are always aggressive and violent"* (IDI #3, Female), with the ensuing conflict leading to family disintegration.

Moreover, several participants (4 of the 19 participants) discuss how a child's mental health is significantly affected by witnessing their parents "*fighting and shouting*" (INT#12, Male), a form of emotional conflict, due to alcohol consumption. As a result, many pre-existing interpersonal relationships were harmed or broken – *"I have witnessed many times that people get drunk and start to say abusive words to one another ...I can say that alcohol can truly bring people together but can also destroy people's relationship"* (IDI #19, Female).

<u>Injuries and Chronic Disease</u>

While all the categories of harms thus far discussed can lead to significant physical, mental, and emotional problems, a plethora of other long-term health issues stemming from alcohol use like injuries and road traffic incidents, illness and chronic disease were also discussed by most participants.

> *"Alcohol can lead to organ damage like liver so there is a lot of complications...I had a friend of mine who was drinking every day, and he was not eating well so he ends up dying. Because of alcohol intoxication. But before that he was having anemia (low blood), low body weight, and muscle wasting" (IDI #12, Male)*

Health issues that were attributed to alcohol use by participants included the following: liver cirrhosis, cancer, kidney failure, "*puffy cheek*" (water retention resulting from alcohol use), neurological insults, and even death. Some participants even recount how local brews —which are "*not tested and not safe*" *(IDI #4, Male)* and have been characterized as "*strong alcohol*" *(IDI #8, Male)*— may incite even greater health consequences in regular consumers.

<u>Financial Instability</u>

Lastly, almost half of the participants mentioned economic loss and financial instability, encouraged via a variety of different mechanisms, as a major source of personal and community

harm. First, participants proposed that economic loss and financial instability seem to arise from the perceived difficulty of drinkers to find or maintain employment. That is, participants agreed that employed individuals who drink experience a loss of productivity. IDI #17, for example, stated, *"Alcohol-related behaviors may lower the individual performance at (the) workplace which leads to inadequate or inefficient production" (IDI #17, Male).*

In addition, if an individual spends *"a lot of money buying alcohol"* (IDI #12, Male) for themselves, this can take a toll on personal savings –*"I lost my business because of drinking too much"* (IDI #12, Male). One participant describes the monetary loss alcohol-related spending could cause:

> "*Alcohol affected my uncle so badly, he was so rich, and helped people in his family and relatives but once he became addicted with alcohol everything turns to zero. He lost everything, even closer friends. He is unable to take care of his family even paying school fees for his children!" (IDI# 5, Male)*

As men especially were seen to be the primary breadwinners for their families, a father's excess spending on alcohol could "*decrease in family income"* (IDI #17, Male). This created negative repercussions for a man's wife and children and permanently cripple their financial holdings:

> *"Alcohol is a reason for many families here to fall into extreme poverty because of men as family providers spend a lot of resources in alcohol while they just earn little to help their families survive as most of them work as drivers of tricycles and motorcycles. So, when a man has no control of his drinking habits and spends all of his income into drinking and definitely the family get into a vicious cycle of poverty." (INT #19)*

While increased alcohol consumption was seen as contributing to personal or familial financial distress for some, as will be discussed in the next section, several participants noted a financial benefit to increased alcohol use for sellers or the government.

Alcohol's Perceived Benefits

When asked how alcohol has positively impacted their community, a third (only one of which was female) commented on the economic gain in alcohol production and sales, and half of the participants commonly mentioned social and/or cultural benefits.

Economic Growth

In contrast to the last sub-theme presented, IDI #2 described the monetary benefit alcohol brought to his community, saying it

> *"contributes to the economic growth of an individual who is selling, a family or a national income. The government collects taxes from the brewing industry, which helps to increase national income" (*IDI# 2, Male*).*

Interestingly though, while the government benefited financially from alcohol sales, several respondents discussed how *"local brew"* (IDI #4, Male) is often not effectively tested for alcohol content levels and results in harmful consequences for their community. One participant explained this lack of government monitoring:

> *"The community views [alcohol] as a burden promoted by the government, it is the government that legalized it and allow people to manufacture alcohol. And sometimes the government fails to monitor these industries and see what kind of alcohol they are producing. It is very unfortunate that some of the businessmen are not faithful and manufacture alcohol which are very strong and damage people's brain." (IDI #12, Male)*

Importantly, while the production of alcohol was seen to bring economic benefit for some, even this perceived boon carried with it the potential for harm to community members.

Upholding Cultural Practices

As mentioned in the first quote in this manuscript, alcohol use was tied to cultural and tribal events – *"Many people consider alcohol to be part of their social culture and way of life"* (IDI #2, Male). Some respondents found it appropriate to consume alcohol at cultural events or special occasions, using alcohol during rituals as a way to "*signify respect and honor to our ancestors*" *(IDI #2, Male)* and "*honor their ancestors' way of living, social customs that have been practiced for generations and generations*" *(IDI #3, Female)*. One woman summarized these sentiments:

> *"There are social events that cannot be done without alcohol, for example, if you go to pay for dowry you have to bring alcohol to the event and even in some rituals, they must brew alcohol during the ritual service. So, there's a positive way alcohol affects our society as a whole…many people drink alcohol to spend time together and enjoy" (IDI #9, Female)*

Social Unity

Finally, alcohol's positive influence on the social aspect of community life was a common theme in IDIs – *"alcohol helps people interact and have time to discuss different issues…it strengthens unity among people" (IDI #4, Male)* and

> *"[Alcohol] helps to bring people together in unity. You may find friends or just random people sitting together, making stories about a thing or two and celebrating their achievement over a glass of beer. In my society, you cannot just gather around grown-up*

people to discuss about something and just give them soda or juice, you must offer them alcohol so that they will listen to you properly" (IDI #19, Female).

"The good results of alcohol, to me personally it has made my social life very great and I have been meeting a lot of people when I go for a drink, for my friends as we enjoy drinks together and talk about our lives but I have also met important and influential people to discuss important matters in business and work as we enjoy 1 or 2 glass of beer" (IDI #10, Female)

Not only was alcohol use noted as bringing people together, but alcohol was also mentioned as having a key role in resolving social conflict and social gatherings:

"If you have misunderstandings with friends or family members, and you want to resolve those disputes…you need to offer them alcohol while you talk! You can't offer them soft drinks! Even when you want to get married, you need to buy a lot of alcohol for elderly people to drink and enjoy" (IDI #2, Male).

Discussion

This paper explored the perceptions of alcohol-related consequences through the lens of KCMC ED and RHC patients. This analysis is the first of its kind to provide a holistic overview of the harms and benefits of alcohol as perceived by patients in Moshi, Tanzania. Existing literature on Moshi has described the scope of alcohol use in this region [26,28,32,55], examined the determinants of use [56], and explored specific consequences related to alcohol, such as drunk driving [57] and stigma [29,33]. Perceptions of alcohol use have been explored [23], but without looking at alcohol-related consequences and exclusively from the perspectives of injury patients. Our paper takes the existing data one step further by providing a comprehensive view of perceived alcohol-related impacts on individuals, their families, and their communities to allow for a clearer picture of alcohol's role in this context. Our results show that individuals and their families experienced alcohol-related social harms like stigma and interpersonal conflict; however, alcohol was also thought to foster unity at the broader community level. From a social dimension, while patients perceived alcohol to have some level of financial benefit on the community and government level through sales and taxes, substantial economic harm from excess spending on alcohol arose for the individuals and families of heavy users. Finally, no health benefits were linked to alcohol consumption, but a slew of physical harms were mentioned by participants.

In this analysis, we found that participants saw both positive and negative impacts of alcohol use at the individual, interpersonal, and community levels, including the perceived benefit of social unity and upholding traditional customs contrasted by the harm of greater conflict and stigma. One stipulation to these results is that while male ED patients had the highest average scores on the DrInC interpersonal subscale, stigma was found to affect women

more severely in our qualitative findings. Looking at the DrInC scale, on average, men responded affirmatively to experiencing approximately three different types of interpersonal consequences related to alcohol use, compared to 0.39 for female RHC patients and one for female ED patients. While these social harms were well documented, social benefits could also be seen as participants mentioned alcohol brought people together and fostered a sense of social unity. These mixed-methods findings, when taken together, demonstrate that the social ramifications of alcohol use are significant but nuanced, and vary by gender. Men, for example, generally experience a wider array of social stipulations, whereas women experience consequences that are more severe.

Previous Moshi-based studies have identified alcohol-related stigma [33,58] and a disproportionate stigma against women [29], however greater exploration on this topic is needed as these data sets were limited to ED injury patients. At the interpersonal level, alcohol use was purported to foster social connections but at the same time had the potential to generate conflict, going as far as destroying romantic and family relationships. Though the first of these findings is not well-supported by academic literature, alcohol has been previously noted to be a mediator in conflict and intimate partner violence both globally and regionally [59–62]. While both social benefits and harms were noted by participants, only the latter is currently supported in scholarly publications. However, further research aimed at understanding the perceived positive associations of alcohol use can help better contextualize its ubiquitous usage in Moshi and pinpoint focus areas for future alcohol-reduction-focused interventions.

Similar to the social implications of alcohol use, we found that alcohol consumption can have important financial consequences at the individual and country levels. IDI participants described a decreased household income that arose from excessive spending on alcohol. This

echoes previously published study data where we found nearly 40% of male ED patients spent between 10 - 46% of overall average monthly household income on alcohol alone [42,63]. This current analysis further supports a gendered component to alcohol-related financial consequences. In our survey data, men had a higher positive response rate to questions like ‘I have missed days or work’ and ‘I have spent too much’ as encapsulated by DrInC’s social responsibility subscale. While women are spending the least amount on alcohol overall, previous data report that women consume less alcohol but also experience limited autonomy when it comes to the use of household funds. Other important economic-based findings from this analysis are the impacts at broader community and government levels, the effects of which were noted by participants to be overall positive. IDI participants believed that high rates of consumption were advantageous for sellers of alcohol and rising national excise taxes on alcohol products contributed to increasing revenue for the government of Tanzania. However, specifics of how this additional revenue was used to benefit Tanzanians were not mentioned.

Currently, while there is a lack of literature outlining the broader financial implications of alcohol use within the East African context. Matzopoulos et al. estimate that within South Africa, the tangible and intangible costs incurred from harmful alcohol consumption comprise between 10-12% of the country’s total GDP and outweigh the economic benefits associated with alcohol taxes and sales [64]. Further, research has suggested the inverse relationship between alcohol consumption and labor force participation globally [65]. This contributes to larger macroeconomic implications, including losses in economic productivity and development. Overall, our data suggests that financial benefits from alcohol use were perceived to only exist for sellers of alcohol and at the government level. For individuals and the broader community, participants almost exclusively noted the negative monetary impacts of alcohol use. Based off

these results and the scarce regional data, we suggest that future research has the opportunity to examine the micro- and macroeconomic implications of alcohol to inform policy recommendations in Tanzania.

Along with the social and financial consequences of alcohol consumption, we noted several physical harms stemming from alcohol use. Male ED patients were seen to experience a wider range of physical consequences than women presenting at the ED and RHC as illustrated by our DrInC physical harm scores. Men answered positively to experiencing 2.54 alcohol-related physical harms compared to 0.60 from female RHC patients and 1.39 female ED patients. IDI data provided more information on the types of physical harm thought to be suffered by this patient population. These listed harms included increases in sexual assault, violence, risky sexual behaviors, and chronic health issues, with no mention of any physical benefit to themselves or their communities. This last finding echoes a recent statement made by the World Health Organization (WHO) stating that any level of drinking is harmful to physical health [66,67]. While this statement and our qualitative data reports no physical benefits with alcohol use, past research has indicated that moderate alcohol intake can have positive circulatory and cardiovascular effects as well as being protective against the incidence of type 2 diabetes. The physical consequences of alcohol use noted by participants have been seen in other Africa-based studies. In sub-Saharan Africa, harmful alcohol use has been previously associated with increased road traffic injuries, drunk driving, and other injurious behaviors [32,57,68,69]. Similar to our results, this past research has found a higher prevalence of alcohol-related physical harm in men. In addition, studies in Uganda and South Africa have found an association between sexual and intimate partner violence and alcohol consumption [70,71]. Last, our IDI data also echoes the WHO and the Center for Disease Control's growing concern over the chronic health

consequences of excess consumption such as liver and kidney failure [72]. Effective alcohol-reduction programs stand to reduce not only the negative health effects associated with alcohol but also violent behaviors that incite injuries. For example, Walton et al. and Ward et al. found, respectively, that interventions aiming to lower alcohol intake subsequently reduce aggression [73,74]. Given the notable physical risk that alcohol poses, there is a need to address alcohol use behaviors in the Moshi area. Additionally, as research has shown that interventions are more effective when culturally adapted to specific populations [75,76], future alcohol-reduction-related programs and policies implemented in Moshi should encompass the specific socio-cultural nuances of the region.

In considering the social, financial, and physical impacts of alcohol use, one final note is that while broader community benefits like social unity, the upholding of traditions, and increased government revenue were raised, participants identified few personal benefits of alcohol use. This raises the question of if alcohol truly has benefits in the community outside of historic incorporation into cultural and social events. Additional research exploring alcohol's impact on the personal versus community levels are thus recommended.

Limitations and Directions for Future Research

Limitations for this analysis are present primarily within the quantitative data set and the DrInC tool. First, the rate of patients who screen failed or declined survey participation was not originally collected. This has an impact on the generalizability of our quantitative data to the ED and RHC patient populations at large. Additionally, this data was collected in the midst of the COVID-19 pandemic. As such, for the safety of our Tanzanian research staff, those who presented to the ED for COVID-19-related symptoms were not approached for study participation. While it was necessary to take these safety precautions, this also limited our ability

to reach the full expected patient population. In both these cases, however, because of the large sample size and well-enforced sampling strategy, we still hold our data to be representative of the patient population. For DrInC, while this tool helps measure the range of consequences one might experience from alcohol use, it does not provide information on how severe these consequences are to the individual, which limits our understanding of alcohol's true impact. Finally, several important gender-specific consequences of alcohol use arose in this analysis, highlighting the need for further exploration of how alcohol differentially impacts men and women in this region. Even with these limitations, our paper offers one of the first published assessments of community perceptions of alcohol use in sub-Saharan Africa, offering insight into how alcohol is viewed both positively and negatively by patients and community members. Our work highlights the need to consider social, financial, and cultural elements when addressing alcohol use disorders. Particularly, we recommend that local policy and healthcare efforts acknowledge and incorporate the roles of alcohol use in future alcohol-related programming and treatment initiatives in the Moshi community.

Conclusion

Alcohol was perceived to have both positive and negative impacts on patients and their communities. Perceived harms included stigma, sexual harm, interpersonal conflict, injuries and disease, and financial instability. These harms stand in contrast to the mentioned benefits of increased social unity, upholding traditional practices, and economic growth. Further, quantitative data collected suggests that men presenting at the ED were found to experience a wider range of alcohol-related consequences than women presenting at either the RHC or ED. Our results allow for a sharper picture of alcohol's role in this community and point to the need to incorporate the sociocultural, financial, and physical implications of alcohol use in programming and treatment related to alcohol use disorders. These findings can help identify focus areas for future programs aiming to reduce alcohol misuse in this area.

Author contributions:

Conceptualization: AMP, CAS, BTM, BM

Methodology: AMP, CAS, JRNV

Formal analysis and investigation: CAS, BTM, BM

Data Collection: MMi, JK, YS, AMP

Writing - original draft preparation: AMP, MB, KW, MM, WN

Writing - review and editing: SR, AMP, MB, KW, MM, WN, CAS

Funding acquisition: AMP, CAS

Supervision: CAS, SR, JRNV, BTM

References

1. World Health Organization (WHO). Global status report on alcohol and health 2018 [Internet]. World Health Organization (WHO); 2018 [cited 2021 Nov 21]. Available from: https://apps.who.int/iris/bitstream/handle/10665/274603/9789241565639-eng.pdf

2. Shield KD, Parry C, Rehm J. Chronic diseases and conditions related to alcohol use. Alcohol Res. 2013;35(2):155–73.

3. Rehm J, Gmel GE, Gmel G, Hasan OSM, Imtiaz S, Popova S, et al. The relationship between different dimensions of alcohol use and the burden of disease-an update. Addiction. 2017 Jun;112(6):968–1001.

4. Rehm J, Mathers C, Popova S, Thavorncharoensap M, Teerawattananon Y, Patra J. Global burden of disease and injury and economic cost attributable to alcohol use and alcohol-use disorders. Lancet. 2009 Jun 27;373(9682):2223–33.

5. Kummetat JL, Leonhard A, Manthey J, Speerforck S, Schomerus G. Understanding the Association between Alcohol Stigma and Alcohol Consumption within Europe: A Cross-Sectional Exploratory Study. Eur Addict Res. 2022 Sep 9;28(6):446–54.

6. Burke TR. The economic impact of alcohol abuse and alcoholism. Public Health Rep. 1988 Dec;103(6):564–8.

7. Thavorncharoensap M, Teerawattananon Y, Yothasamut J, Lertpitakpong C, Chaikledkaew U. The economic impact of alcohol consumption: a systematic review. Subst Abuse Treat Prev Policy. 2009 Nov 25;4:20.

8. Manthey J, Hassan SA, Carr S, Kilian C, Kuitunen-Paul S, Rehm J. What are the economic costs to society attributable to alcohol use? A systematic review and modelling study. Pharmacoeconomics. 2021 Jul;39(7):809–22.

9. Schomerus G, Lucht M, Holzinger A, Matschinger H, Carta MG, Angermeyer MC. The stigma of alcohol dependence compared with other mental disorders: a review of population studies. Alcohol Alcohol. 2011 Apr;46(2):105–12.

10. Birtel MD, Wood L, Kempa NJ. Stigma and social support in substance abuse: Implications for mental health and well-being. Psychiatry Res. 2017 Jun;252:1–8.

11. Pitpitan EV, Kalichman SC, Eaton LA, Cain D, Sikkema KJ, Skinner D, et al. Gender-based violence, alcohol use, and sexual risk among female patrons of drinking venues in Cape Town, South Africa. J Behav Med. 2013 Jun;36(3):295–304.

12. Schulkind J, Mbonye M, Watts C, Seeley J. The social context of gender-based violence, alcohol use and HIV risk among women involved in high-risk sexual behaviour and their intimate partners in Kampala, Uganda. Cult Health Sex. 2016 Jul;18(7):770–84.

13. Bonner CP, Browne FA, Ndirangu JW, Howard B, Zule WA, Speizer IS, et al. Exploring

the associations between physical and sexual gender-based violence and HIV among women who use substances in South Africa: the role of agency and alcohol. AIDS Care. 2019 Nov;31(11):1369–75.

14. Walls H, Cook S, Matzopoulos R, London L. Advancing alcohol research in low-income and middle-income countries: a global alcohol environment framework. BMJ Glob Health. 2020 Apr 16;5(4):e001958.

15. Marten R, Amul GGH, Casswell S. Alcohol: global health's blind spot. Lancet Glob Health. 2020 Mar;8(3):e329–30.

16. Ferreira-Borges C, Parry CDH, Babor TF. Harmful Use of Alcohol: A Shadow over Sub-Saharan Africa in Need of Workable Solutions. Int J Environ Res Public Health. 2017 Mar 27;14(4).

17. Lim SS, Vos T, Flaxman AD, Danaei G, Shibuya K, Adair-Rohani H, et al. A comparative risk assessment of burden of disease and injury attributable to 67 risk factors and risk factor clusters in 21 regions, 1990-2010: a systematic analysis for the Global Burden of Disease Study 2010. Lancet. 2012 Dec 15;380(9859):2224–60.

18. Murray et al. CJL. Global, regional, and national comparative risk assessment of 79 behavioural, environmental and occupational, and metabolic risks or clusters of risks, 1990-2015: a systematic analysis for the Global Burden of Disease Study 2015. Lancet. 2016 Oct 8;388(10053):1659–724.

19. Shield KD, Rehm J. Societal development and the alcohol-attributable burden of disease. Addiction. 2021 Sep;116(9):2326–38.

20. Probst C, Kilian C, Sanchez S, Lange S, Rehm J. The role of alcohol use and drinking patterns in socioeconomic inequalities in mortality: a systematic review. Lancet Public Health. 2020 Jun;5(6):e324–32.

21. Katikireddi SV, Whitley E, Lewsey J, Gray L, Leyland AH. Socioeconomic status as an effect modifier of alcohol consumption and harm: analysis of linked cohort data. Lancet Public Health. 2017 Jun;2(6):e267–76.

22. Sudhinaraset M, Wigglesworth C, Takeuchi DT. Social and Cultural Contexts of Alcohol Use: Influences in a Social-Ecological Framework. Alcohol Res. 2016;38(1):35–45.

23. Meier BJ, El-Gabri D, Friedman K, Mvungi M, Mmbaga BT, Nickenig Vissoci JR, et al. Perceptions of alcohol use among injury patients and their family members in Tanzanian society. Alcohol. 2020 Mar;83:9–15.

24. WHO. Alcohol [Fact sheet] [Internet]. World Health Organization. 2022 [cited 2023 Jan 23]. Available from: https://www.who.int/news-room/fact-sheets/detail/alcohol

25. WHO. Global Strategy to Reduce the Harmful Use of Alcohol. Geneva: World Health Organization; 2010.

26. Mitsunaga T, Larsen U. Prevalence of and risk factors associated with alcohol abuse in Moshi, northern Tanzania. J Biosoc Sci. 2008 May;40(3):379–99.

27. Francis JM, Weiss HA, Mshana G, Baisley K, Grosskurth H, Kapiga SH. The Epidemiology of Alcohol Use and Alcohol Use Disorders among Young People in Northern Tanzania. PLoS ONE. 2015 Oct 7;10(10):e0140041.

28. Mushi D, Moshiro C, Hanlon C, Francis JM, Teferra S. Missed opportunity for alcohol use disorder screening and management in primary health care facilities in northern rural Tanzania: a cross-sectional survey. Subst Abuse Treat Prev Policy. 2022 Jul 6;17(1):50.

29. Griffin SM, Karia FP, Zimmerman A, Minnig MCC, Swahn M, Makelarski J, et al. A Mixed-Methods Study: Sex Differences in Experiences of Stigma Associated With Alcoholism and Alcohol Use Disorders Among Injury Patients in Tanzania. Alcohol Clin Exp Res. 2020 Aug 9;44(8):1700–7.

30. Norris AH, Kitali AJ, Worby E. Alcohol and transactional sex: how risky is the mix? Soc Sci Med. 2009 Oct;69(8):1167–76.

31. Cooper ML. Alcohol use and risky sexual behavior among college students and youth: evaluating the evidence. J Stud Alcohol Suppl. 2002 Mar;(s14):101–17.

32. Staton CA, Vissoci JRN, Toomey N, Abdelgadir J, Chou P, Haglund M, et al. The impact of alcohol among injury patients in Moshi, Tanzania: a nested case-crossover study. BMC Public Health. 2018 Feb 21;18(1):275.

33. El-Gabri D, Nickenig Vissoci JR, Meier BJ, Mvungi M, Haglund M, Swahn M, et al. Alcohol stigma as it relates to drinking behaviors and perceptions of drink drivers: A mixed method study in Moshi, Tanzania. Alcohol. 2020 Nov;88:73–81.

34. Zimmerman A, Pesambili M, Phillips AJ, Boshe J, Mmbaga BT, Pantalon MH, et al. Cultural adaptation of a brief intervention to reduce alcohol use among injury patients in tanzania. medRxiv. 2021 Jul 22;

35. Staton CA, Zadey S, O'Leary P, Phillips A, Minja L, Swahn MH, et al. PRACT: a pragmatic randomized adaptive clinical trial protocol to investigate a culturally adapted brief negotiational intervention for alcohol use in the emergency department in Tanzania. Trials. 2022 Feb 5;23(1):120.

36. Blas E Ed. Equity, Social Determinants and Public Health Programmes. 1st ed. Geneva, Switzerland: World Health Organization; 2010.

37. Cherpitel CJ, Bond J, Ye Y, Borges G, Macdonald S, Giesbrecht N. A cross-national meta-analysis of alcohol and injury: data from the Emergency Room Collaborative Alcohol Analysis Project (ERCAAP). Addiction. 2003 Sep;98(9):1277–86.

38. WHO. Alcohol and Injury in Emergency Departments. World Health Organization. 2007;

39. Staton CA, Friedman K, Phillips AJ, Minnig MC, Sakita FM, Ngowi KM, et al.

Feasibility of a pragmatic randomized adaptive clinical trial to evaluate a brief negotiational interview for harmful and hazardous alcohol use in Moshi, Tanzania. PLoS ONE. 2023 Aug 3;18(8):e0288458. pmid:37535693

40. Isaksen, A.B., Østbye, T., Mmbaga, B.T. et al. Alcohol consumption among pregnant women in Northern Tanzania 2000–2010: a registry-based study. BMC Pregnancy Childbirth 15, 205 (2015). https://doi.org/10.1186/s12884-015-0630-0

41. Mpelo M, Kibusi SM, Moshi F, Nyundo A, Ntwenya JE, Mpondo BCT. Prevalence and Factors Influencing Alcohol Use in Pregnancy among Women Attending Antenatal Care in Dodoma Region, Tanzania: A Cross-Sectional Study. J Pregnancy. 2018 Oct 18;2018:8580318. doi: 10.1155/2018/8580318. PMID: 30420920; PMCID: PMC6211147.

42. Pauley A, Buono M, West K, Metcalf M, Rent S, Kilasara J, et al. A Mixed-Methods Comparison of Gender Differences in Alcohol Consumption and Drinking Characteristics among Patients in Moshi, Tanzania. medRxiv. 2023 May 18;

43. Zhao D, Staton CA, He Q, Mmbaga BT, Vissoci JRN. Cross-Culture Adaptation and Psychometric Properties of the DrInC Questionnaire in Tanzanian Swahili. Front Public Health. 2018 Nov 20;6:330.

44. Miller WR, Tonigan S, Longabaugh R. THE DRINKER INVENTORY OF CONSEQUENCES (DrInC). Alcohol Res Health. 1995;

45. Kirouac M, Witkiewitz K. Revisiting the Drinker Inventory of Consequences: An extensive evaluation of psychometric properties in two alcohol clinical trials. Psychol Addict Behav. 2018;32(1):52–63.

46. Nadkarni A, Garber A, Costa S, Wood S, Kumar S, MacKinnon N, et al. Auditing the AUDIT: A systematic review of cut-off scores for the Alcohol Use Disorders Identification Test (AUDIT) in low- and middle-income countries. Drug Alcohol Depend. 2019 Sep 1;202:123–33.

47. Bohn MJ, Babor TF, Kranzler HR. The Alcohol Use Disorders Identification Test (AUDIT): validation of a screening instrument for use in medical settings. J Stud Alcohol. 1995 Jul;56(4):423–32.

48. Vissoci JRN, Hertz J, El-Gabri D, Andrade Do Nascimento JR, Pestillo De Oliveira L, Mmbaga BT, et al. Cross-Cultural Adaptation and Psychometric Properties of the AUDIT and CAGE Questionnaires in Tanzanian Swahili for a Traumatic Brain Injury Population. Alcohol Alcohol. 2018 Jan 1;53(1):112–20.

49. Saunders JB, Aasland OG, Babor TF, de la Fuente JR, Grant M. Development of the Alcohol Use Disorders Identification Test (AUDIT): WHO Collaborative Project on Early Detection of Persons with Harmful Alcohol Consumption--II. Addiction. 1993 Jun;88(6):791–804.

50. Hennink M, Kaiser BN. Sample sizes for saturation in qualitative research: A systematic review of empirical tests. Soc Sci Med. 2022 Jan;292:114523.

51. McQueen K, Namey E, Guest G. Applied Thematic Analysis. 2014, editor. SAGE Publications Inc; 2014.

52. Foley G, Timonen V. Using Grounded Theory Method to Capture and Analyze Health Care Experiences. Health Serv Res. 2015 Aug;50(4):1195-210. doi: 10.1111/1475-6773.12275. Epub 2014 Dec 18. PMID: 25523315; PMCID: PMC4545354.

53. Turner C, Astin F. Grounded theory: what makes a grounded theory study? Eur J Cardiovasc Nurs. 2021 Apr 28;20(3):285-289. Doi: 10.1093/eurjcn/zvaa034. PMID: 33772270.

54. Chapman AL, Hadfield M, Chapman CJ. Qualitative research in healthcare: an introduction to grounded theory using thematic analysis. J R Coll Physicians Edinb. 2015;45(3):201-5. doi: 10.4997/JRCPE.2015.305. PMID: 26517098.

55. Staton CA, Zhao D, Ginalis EE, Hirshon JM, Sakita F, Swahn MH, et al. Alcohol Availability, Cost, Age of First Drink, and Its Association with At-Risk Alcohol Use in Moshi, Tanzania. Alcohol Clin Exp Res. 2020 Nov 10;44(11):2266–74.

56. Ao TT-H, Sam N, Kiwelu I, Mahal A, Subramanian SV, Wyshak G, et al. Risk factors of alcohol problem drinking among female bar/hotel workers in Moshi, Tanzania: a multi-level analysis. AIDS Behav. 2011 Feb;15(2):330–9.

57. El-Gabri D, Barcenas LK, Meier B, Mvungi M, Haglund M, Gerardo CJ, et al. Injury patients' perceptions of drink-driving: A qualitative assessment of drink-driving behavior in Moshi, Tanzania. PLoS ONE. 2020 May 5;15(5):e0230662.

58. Zhao D, Nickenig Vissoci JR, Mmbaga BT, Abdullah AS, Staton CA. Alcohol-Related Risky Behavior Patterns and Their Association With Alcohol Use and Perceived Alcohol Stigma in Moshi, Tanzania. J Stud Alcohol Drugs. 2020 Sep;81(5):614–23.

59. Zaleski M, Pinsky I, Laranjeira R, Ramisetty-Mikler S, Caetano R. Intimate partner violence and alcohol consumption. Rev Saude Publica. 2010 Feb;44(1):53–9.

60. Foran HM, O'Leary KD. Alcohol and intimate partner violence: a meta-analytic review. Clin Psychol Rev. 2008 Oct;28(7):1222–34.

61. Messersmith LJ, Halim N, Steven Mzilangwe E, Reich N, Badi L, Holmes NB, et al. Childhood trauma, gender inequitable attitudes, alcohol use and multiple sexual partners: correlates of intimate partner violence in northern tanzania. J Interpers Violence. 2021 Jan;36(1–2):820–42.

62. Mootz JJ, Muhanguzi FK, Panko P, Mangen PO, Wainberg ML, Pinsky I, et al. Armed conflict, alcohol misuse, decision-making, and intimate partner violence among women in Northeastern Uganda: a population level study. Confl Health. 2018 Aug 6;12:37.

63. Pauley A, Thatcher EC, Sarafian JT, Zadey S, Shayo F, Mmbaga BT, et al. Alcohol Use among Emergency Medicine Department Patients in Tanzania: A Comparative Analysis of Injury Versus Non-Injury Patients. medRxiv. 2023 May 4;

64. Matzopoulos RG, Truen S, Bowman B, Corrigall J. The cost of harmful alcohol use in South Africa. S Afr Med J. 2014 Feb;104(2):127–32.

65. Jørgensen MB, Pedersen J, Thygesen LC, Lau CJ, Christensen AI, Becker U, et al. Alcohol consumption and labour market participation: a prospective cohort study of transitions between work, unemployment, sickness absence, and social benefits. Eur J Epidemiol. 2019 Apr;34(4):397–407.

66. WHO. No level of alcohol consumption is safe for our health [Internet]. World Health Organization. 2023 [cited 2023 May 11]. Available from: https://www.who.int/europe/news/item/04-01-2023-no-level-of-alcohol-consumption-is-safe-for-our-health

67. Anderson BO, Berdzuli N, Ilbawi A, Kestel D, Kluge HP, Krech R, et al. Health and cancer risks associated with low levels of alcohol consumption. Lancet Public Health. 2023 Jan;8(1):e6–7.

68. Kalichman SC, Simbayi LC, Kaufman M, Cain D, Jooste S. Alcohol use and sexual risks for HIV/AIDS in sub-Saharan Africa: systematic review of empirical findings. Prev Sci. 2007 Jun;8(2):141–51.

69. Ferreira-Borges C, Rehm J, Dias S, Babor T, Parry CDH. The impact of alcohol consumption on African people in 2012: an analysis of burden of disease. Trop Med Int Health. 2016 Jan;21(1):52–60.

70. Bello B, Moultrie H, Somji A, Chersich MF, Watts C, Delany-Moretlwe S. Alcohol use and sexual risk behaviour among men and women in inner-city Johannesburg, South Africa. BMC Public Health. 2017 Jul 4;17(Suppl 3):548.

71. Zablotska IB, Gray RH, Koenig MA, Serwadda D, Nalugoda F, Kigozi G, et al. Alcohol use, intimate partner violence, sexual coercion and HIV among women aged 15-24 in Rakai, Uganda. AIDS Behav. 2009 Apr;13(2):225–33.

72. Center for Disease Control and Prevention. Alcohol Use and Your Health [Internet]. Alcohol and Public Health. 2022 [cited 2023 May 10]. Available from: https://www.cdc.gov/alcohol/fact-sheets/alcohol-use.htm#:~:text=Over%20time%2C%20excessive%20alcohol%20use,liver%20disease%2C%20and%20digestive%20problems.&text=Cancer%20of%20the%20breast%2C%20mouth,liver%2C%20colon%2C%20and%20rectum

73. Walton MA, Chermack ST, Shope JT, Bingham CR, Zimmerman MA, Blow FC, et al. Effects of a brief intervention for reducing violence and alcohol misuse among adolescents: a randomized controlled trial. JAMA. 2010 Aug 4;304(5):527–35.

74. Ward CL, Mertens JR, Bresick GF, Little F, Weisner CM. Screening and Brief Intervention for Substance Misuse: Does It Reduce Aggression and HIV-Related Risk Behaviours? Alcohol Alcohol. 2015 May 1;50(3):302–9.

75. Griner D, Smith TB. Culturally adapted mental health intervention: A meta-analytic review. Psychotherapy. 2006;43(4):531–48.

76. Huey SJ, Polo AJ. Evidence-based psychosocial treatments for ethnic minority youth. J Clin Child Adolesc Psychol. 2008 Jan;37(1):262–301.

Supporting Information

1. Fig1
2. Fig2
3. S1 Checklist – STROBE Checklist
4. S2 Checklist – PLOS ONE Clinical Studies Checklist
5. S3 Checklist – PLOS ONE Human Subjects Research Checklist
6. S1 Questionnaire – PLOS Inclusivity in Global Research Questionnaire

Other

1. S1 File – Duke University Health System Institutional Review Board Approval Notice
2. S2 File – Kilimanjaro Christian Medical University College Research Ethical Clearance Certificate
3. S3 File – National Institute for Medical Research (Tanzania) Research Ethical Clearance Certificate

Figure 1

Click here to access/download;Figure;Fig1.tiff

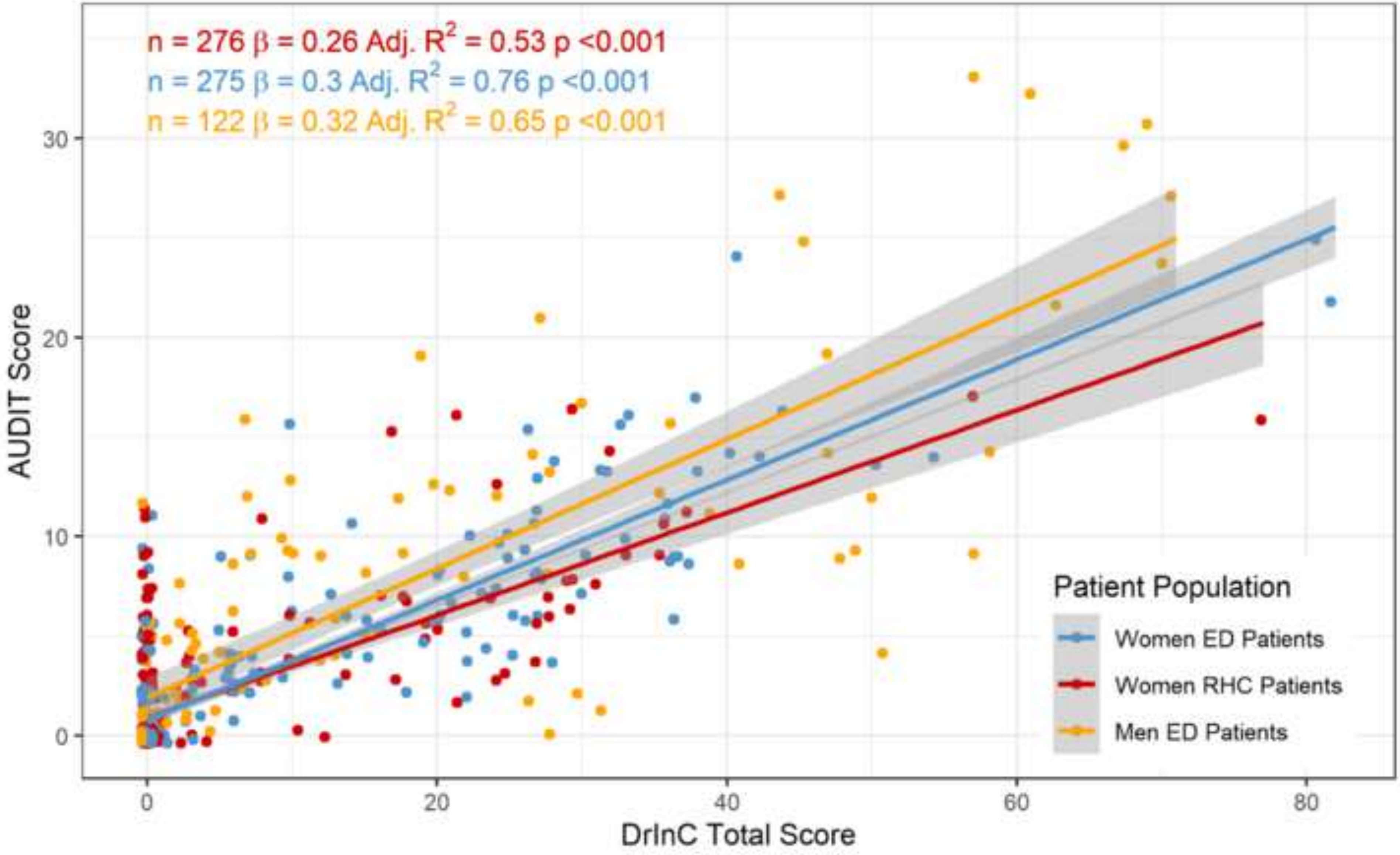

Figure 2

Click here to access/download;Figure;Fig2.tiff

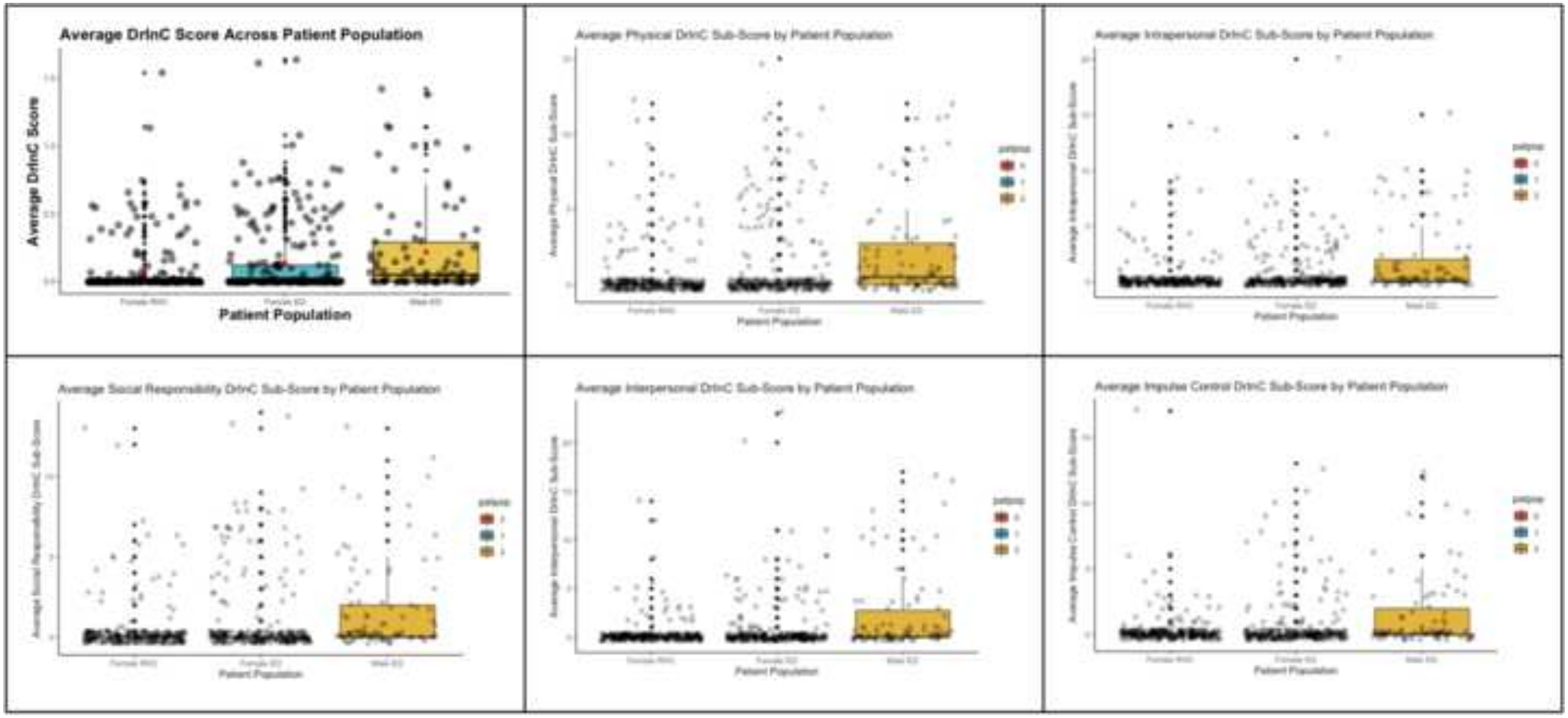

Click here to access/download
Supporting Information
S1_STROBE_Checklist.docx

PLOSE ONE Clinical Studies Checklist

Click here to access/download
Supporting Information
S2_PLOS_Checklist.docx

Click here to access/download
Supporting Information
S1_Inclusivity_Questionnaire.docx

Click here to access/download
Supporting Information
S3_HumanSubjects_Checklist.docx

Click here to access/download
Supporting Information
journal.pgph.0002009.pdf

Understanding the Impacts and Perceptions of Alcohol Use in Northern Tanzania: A Mixed-Methods Analysis

Authors and affiliations:

Alena Pauley MScGH[1] – alena.pauley@duke.edu

Madeline Metcalf[1] – madeline.metcalf@duke.edu

Mia Buono[1] – mia.buono@duke.edu

Kirstin West[1] – kirstin.west@duke.edu

Sharla Rent MD MScGH[1,2] - Sharla.Rent@duke.edu

William Nkenguye[3,4] –williamnkenguye@gmail.com

Yvonne Sawe[3] – yvonnesawe90@gmail.com

Mariana Mikindo[3] – mirianamikindo@gmail.com

Joseph Kilasara APRN MNS-FNP[3,5] – joseph.kilasara@kcmuco.ac.tz

Bariki Mchome MD PHD[3,6] – Barikimchome@gmail.com

Blandina T. Mmbaga, MD PhD[1,3,7] – blaymt@gmail.com

João Ricardo Nickenig Vissoci MBA MSc PhD[1,8] – jnv4@duke.edu

Catherine A. Staton MD MScGH[1,8] – catherine.lynch@duke.edu

1 Duke Global Health Institute, Duke University, Durham, NC USA

2 Duke Department of Pediatrics, Duke University, Durham, NC USA

3 Kilimanjaro Christian Medical Center, Moshi, Tanzania

4 Kilimanjaro Christian Medical University College, Moshi, Tanzania

5 Department of Clinical Nursing, Kilimanjaro Christian Medical University College, Moshi, Tanzania

6 Department of Obstetrics and Gynecology, Kilimanjaro Christian Medical University College, Moshi, Tanzania

7 Kilimanjaro Clinical Research Institute, Moshi, Tanzania

8 Duke Department of Surgery, Duke University Medical Center, Durham, NC USA

Funding: This project was funded by the Duke Global Health Institute Graduate Student funds (AMP), and the Josiah Trent Foundation (21-06 to CAS). These two financial awards funded the salaries of JK, YS, and MMi as research assistants hired specifically for this study. No other authors received specific funding for this work. Infrastructure built by NIH grants (R01 AA027512 and D43TW012205 to CAS) was used to support the data collection process for this grant to understand gender-related aspects of alcohol use at KCMC. The funders had no role in study design, data collection and analysis, decision to publish, or preparation of the manuscript.~~This research was supported by the Duke Global Health Institute and the Josiah Charles Trent Memorial Foundation Endowment Fund.~~

Competing interests: The authors declare no competing interests

~~**Author contributions:**~~

~~Conceptualization: AMP, CAS, BTM, BM~~

~~Methodology: AMP, CAS, JRNV~~

~~Formal analysis and investigation: CAS, BTM, BM~~

~~Data Collection: MMi, JK, YS, AMP~~

~~Writing - original draft preparation: AMP, MB, KW, MM, WN~~

~~Writing - review and editing: SR, AMP, MB, KW, MM, WN, CAS~~

Funding acquisition: AMP, CAS

Supervision: CAS, SR, JRNV, BTM

~~**Understanding the Individual and Community-Wide Impacts and Perceptions of Alcohol Use in Northern Tanzania: A Mixed-Methods Analysis**~~

Abstract

Background: Worldwide, alcohol is a leading risk factor for death and disability. Tanzania has particularly high rates of consumption and few resources dedicated to minimizing alcohol-related harm. Ongoing policy efforts are hampered by dynamic sociocultural, economic, and regulatory factors contributing to alcohol consumption. Through the voices of Kilimanjaro Christian Medical Center (KCMC) patients~~ and a gender-focused lens~~, this study aimed to~~e goal of this project was to~~ investigate community perceptions surrounding alcohol and the impact of its use in this region. **Methods**: This was a mixed-methods study conducted at KCMC between October 2021 and May 2022. 676~~8~~ adult (≥18 years old) Kiswahili-speaking patients who presented to KCMC's Emergency Department (ED) or Reproductive Health Clinic (RHC) were enrolled through systematic random sampling to participate in quantitative surveys. Nineteen participants were selected for in-depth interviews (IDIs) through purposeful sampling. The impact and perceptions of alcohol use were measured through Drinkers' Inventory of Consequences (DrInC) scores and analyzed in R-Studio ~~through~~ using descriptive proportions. ~~, and~~ IDI responses were explored through a grounded theory approach using~~tilizing~~ both inductive and deductive coding methodologies in NVivo.~~.~~ **Results:** ~~ED m~~Men attending the ED were found to have the highest average [SD] DrInC scores (16.4 [19.6]), followed by ED women (9.11 [13.1]), and RHC women patients (5.47 [9.33]), with higher scores indicating ~~greater perceived~~a broader array of consequences. Participants noted alcohol ~~has~~ to have both perceived advantages and clear harms within their community. Increased conflict, long-term health outcomes, financial instability,

Formatted: Font: (Default) Times New Roman, Italic

Formatted: Heading 1, Space Before: 0 pt, After: 0 pt, Line spacing: single

Formatted: Font: (Default) Times New Roman, 12 pt, Bold

Formatted: Font: (Default) Times New Roman, 12 pt, Bold

Formatted: Font: (Default) Times New Roman, 12 pt, Bold

stigma, and sexual assault were seen as negative consequences. Benefits were primarily identified for men and included upholding cultural practices, economic growth, and social unity. Physical and financial harm from alcohol impacted both genders, however, alcohol-related stigma and sexual assault were found to disproportionately affect women. **Conclusion**: Our findings suggest that perceptions around drinking are nuanced and alcohol's social and physical consequences differ significantly by gender. To effectively minimize local alcohol-related harm, future alcohol-focused research and policy efforts should consider the complex sociocultural role that alcohol holds in the Moshi community.~~distinct impacts alcohol has between genders.~~

Formatted: Font: (Default) Times New Roman, 12 pt, Bold

Introduction

Alcohol consumption has been implicated as a leading cause of harm, disability, and death worldwide [1]. The harmful use of alcohol is a growing and avoidable cause of premature disability and death, each year causing 5.1% of all disability-adjusted life years (DALYs) and 3 million deaths globally [1]. Alcohol use has been linked to over 200 adverse medical conditions, including digestive diseases, infectious diseases, cardiovascular disease, certain cancers, and injuries [2–4], and has also been associated with poor employment outcomes [5], individual and societal economic harm [4,6–8], social stigma [9,10], and sexual violence [11–13].

~~Although~~While hazardous alcohol consumption is a global ~~problem~~issue, ~~Although a global problem, alcohol~~ its use is increasing especially rapidly in low- and middle-income countries [14–16]. For example, alcohol use has become the leading risk factor for disease burden in many sub-Saharan African countries including Tanzania [17,18]. As low-income populations typically have fewer resources, financial savings, and poorer access to health care services, alcohol-related harm in these contexts is more devastating both physically and financially [1,19–21]. ~~While~~ Though the harms of alcohol intake are significant, its use remains endemic and has been ingrained in cultural and social practices in ~~different~~ populations across the world [22]. This holds true in Tanzania, where the cultural and social practices of alcohol use, such as the normalization of ~~use~~ intake starting from a young age ~~and~~ or use as a social currency, support larger quantities of consumption [23].

Adding greater complexity to alcohol's impact on individuals and communities, alcohol consumption has been shown to differ in populations according to cultural norms, regulations, age, and socio-economic status~~, etc~~ [24]. To address the factors influencing alcohol consumption, the World Health Organization (WHO) created the Conceptual Model of Alcohol Consumption

and Health Outcomes. The model aims to decrease the morbidity and mortality that follow harmful alcohol use patterns and demonstrates that understanding an individual country's sociocultural context is imperative to alter alcohol use behaviors at an individual and country level [25].

Moshi, located near Mount Kilimanjaro National Park in Tanzania, has seen exceptionally high rates of alcohol use, 2.5 times higher than in nearby regions [26,27]. Previous studies have shown that 60% of Moshi residents frequently consume alcohol, and 23% suffer from alcohol use disorder [28]. In this location, alcohol consumption has also been linked~~ here~~ to ~~greater ~~social stigma and ostracization around female alcohol users [29], unsafe sexual behaviors [30,31], and an increased prevalence of injuries among both men and women [32]. While alcohol-related harms have been identified in Moshi, current literature specifying how community members view the harms and benefits of alcohol holistically is sparse.

Previous work from our team has examined alcohol-related stigma [29,33], perceptions of alcohol use [23], the prevalence of alcohol-related injuries upon Emergency Department (ED) arrival [32], and the development~~ing~~ and implementation~~ing~~ ~~an ~~of interventions to reduce alcohol misuse for the ED injury patient population [34,35]. However, ~~we have yet to~~current literature has yet to explore ~~understand ~~~~Building off of this research, a greater understanding of~~ the specific societal perceptions and implications of alcohol use within the Moshi community. Understanding the full scope of alcohol's impact and why its use remains endemic is essential in mitigating its effects. ~~will allow for a clearer picture of its role within the Moshi community.~~ ~~This, in turn,~~This knowledge~~ can help~~ can be used to better inform ~~and shape ~~future alcohol-reduction interventions and research initiatives to be more effective and successful in decreasing alcohol-related harm in this setting [36]. This process of development can also serve as a model

for population-specific harm reduction interventions in other settings. Thus, through the voices of patients at the Kilimanjaro Christian Medical Center (KCMC), this analysisreport fillsaddressed thise gap study aims to fill this gap by investigating the societal perceptions of alcohol along with the negative and positive ways that alcohol use impacts individuals and the broader Moshi community along with perceptions of alcohol use. Factors underlying differential expectations of use will also be explored.

Methods

Study Overview and ~~Design~~Setting

This cross-sectional study used a sequential explanatory mixed~~-~~-methods approach to explore~~is a secondary analysis of a mixed-methods study on~~ ~~gender differences~~ community perceptions and implications of alcohol~~in alcohol~~ use among ~~KCMC's~~ Emergency Department (ED) and Reproductive Health Center (RHC) patients at KCMC. All data was collected between October 2021 and May 2022. This mixed-methods approach was chosen to facilitate the triangulation of data so that robust and accurate epidemiological conclusions could be drawn while also providing deeper cultural context for the complex social, emotional, and physical implications of alcohol use in this community. ~~in Moshi, Tanzania.~~ ~~The ED was chosen as one of the study sites to better understand risky alcohol use behaviors given the correlation between excessive alcohol use and injuries that prompt ED visits [32,37,38]. The RHC, on the other hand, was selected because of its primarily female patient population, which facilitated a richer, gynocentric perspective on alcohol use.~~

KCMC, which is located near the border of Kenya and Kilimanjaro National Park, was chosen as the study setting as it is the largest referral and teaching hospital in the Kilimanjaro region, has existing research infrastructure, and is in an area known to have especially high rates of alcohol use. The ED was selected as one of the clinical study sites to better understand risky alcohol use behaviors given the correlation between excessive alcohol use and injuries like road traffic accidents and violence necessitating emergency care [32,37,38]. Additionally, previous work from our team in developing alcohol-reduction interventions at KCMC's ED meant that continued research in this unit would align with programmatic goals and increase the likelihood of intervention success [39]. The RHC, on the other hand, was chosen because of its primarily

female patient population, which facilitated a richer, gynocentric perspective on alcohol use which has been under-represented in past local research. Measuring alcohol use in pregnant or postpartum women seeking gynecological care was also important for maternal and child health given the high rates of alcohol use during pregnancy previously described in this setting [40,41]. As such, enrolling patients from these two clinical units allowed us to better explore the intersection of gender and hazardous drinking, the primary goal of the overarching study.

Cross-sectional dData for this study were collected between October 2021 and May 2022 and consisted of descriptive quantitative surveys and qualitative in-depth interviews (IDIs). During this time, 678 patients were enrolled, but only 655 participants fully completing survey questionnaires. These participants were enrolled through a systematic random sampling strategy to ensure a balanced representation of each patient population. Of the pool of individuals already participating in this study, 19 were chosen via purposive sampling for IDIs. A full description of the methods for this project is noted elsewhere [39].

This current analysis seeks to understand 1) the impacts alcohol use has on patients themselves and 2) how they perceive alcohol to affect their community. In answering these questions, a quantitative tool that assesses an individual's alcohol-related consequences, the Drinkers Inventory of Consequences (DrInC), was used in addition to a qualitative exploration of the perceived harms and benefits of alcohol use. The mixed-methods approach of this study provides context encompassing the social, emotional, and physical implications of alcohol use in addition to epidemiological evidence.

Study PopulationSample

Formatted: Font: (Default) Times New Roman, 15 pt, Italic

All participants were recruited from KCMC's RHC or ED. The KCMC ED is the main referral facility for all injury patients in the Kilimanjaro region; thus, the ED The ED was chosen

as one of the clinical study sites to better understand risky alcohol use behaviors given the correlation between excessive alcohol use and injuries that prompt ED visits such as road traffic incidents and interpersonal violence [32,37,38]. The RHC, on the other hand, was selected because of its primarily female patient population, which facilitated a richer, gynocentric perspective on alcohol use. Additionally, measuring alcohol use in pregnant or postpartum women is of particular importance for protecting the health and safety of mothers and babies. Those All enrolled patients met the following inclusion eligibility criteria: 1) fluency in Kiswahili, 2) ability to provide informed consent, 3) aged 18 or older, and 4) not a prisoner, and 5) received initial care at KCMC's ED or RHC. Perceived or measured alcohol use was not considered when determining patient eligibility given that the study intended to provide a representative sample offrom the ED and RHC. whether individuals were tentatively eligible for participation in the study. Prior to administering the survey, all patients verbally confirmed that they had not been previously enrolled in the study. Ability to provide informed consent for participation in the study was defined as being medically stable, clinically sober from alcohol, and ability to complete the verbal survey independently. Patients who did not meet these criteria upon initial approach were reevaluated for participation within 24 hours of arrival at KCMC or prior to discharge. Lastly, we found it important to include women attending the RHC to be included in this study due to the adverse effects of alcohol use during pregnancy and perceptions of this use.

Quantitative Data

Study Design

Cross-sectional, quantitative survey data assessing demographic factors, alcohol use, and alcohol-related consequences were collected from KCMC ED and RHC patients. Preliminary

Formatted: Normal

Formatted: Font: (Default) Times New Roman, 12 pt

quantitative analyses were used to guide our qualitative sampling strategy as will be discussed in detail further below.

Sample Size Estimation

Quantitative sample size was calculated based on the difference in average Alcohol Use Disorder Identification Test (AUDIT) scores between our RHC and ED patient populations as the prevalence of alcohol use disorder (AUD) was a primary point of interest in the broader study [42]. Based on existing literature, we hypothesized the prevalence of AUD to be 10% among RHC women, 15% among ED women, and 30% among ED men. Thus, at 80% power and with 90% confidence, ~~An initial sample size calculation conducted estimated that~~ a ~~final~~ sample size of 587 participants ~~would~~was calculated to sufficiently estimate the prevalence of risky ~~drink as defined by the Alcohol Use Disorder Identification Test (AUDIT)~~drinking between all three study subgroups ~~with 80% power and 90% confidence~~. Given that the expected difference in the prevalence of AUD was narrower between the two female populations than between either male and female groups, the study team planned to enroll significantly more women than men.

Preliminary data analysis revealed, however, that the prevalence of AUD was 40% in ED women and 45% in ED men, indicating that now 1,200 patients would need to be enrolled to adequately determine a difference in proportions between these two groups. ~~the beginning stages of data collection, a difference in the proportion of risky drinking between men and women which guided prior sample size calculations was detected.~~ While the study timeline and funding could not accommodate the newly estimated sample size of 1200 participants, with IRB approval, the study's target sample size was increased to enroll as many participants as feasible during the study timeline. ~~A more comprehensive explanation of this sample size estimation can be found in our recently published manuscript.~~

Formatted: Font: 11 pt, Not Italic

Formatted: Normal, Indent: First line: 0.5"

Formatted: Font: (Default) Arial, 11 pt, Not Italic

Sampling Technique

Over the course of the study timeline, 676 patients were enrolled through a systematic random sampling strategy, with 655 participants fully completing survey questionnaires. A systematic random sampling strategy was employed to acquire a representative sample of our three patient populations, male ED, female ED patients, and female RHC patients, and better describe the characteristics of each. Except for ED females, every third patient was approached for potential using the ED triage or RHC intake registries. For women seeking ED care, every eligible individual was approached for study participation. This was done as significantly fewer women seek care at the ED than men and more women in this particular study to accurately determine the prevalence of AUD. ~~A systematic random sampling strategy was used to guide participant enrollment, where every third patient was approached based on ED and RHC intake registries, with the exception of ED females. Given that KCMC's ED patient population is heavily male, every female who presented to the ED was approached to maintain enrollment goals and recruit a representative study sample.~~

Data Collection Method

KCMC ED and RHC patients were approached for participation in this study by a same-gender, Kiswahili-speaking, Tanzanian research assistant trained in g~~G~~ood c~~C~~linical p~~P~~ractices and specific study procedures. ~~A systematic random sampling strategy was used to guide enrollment, where every third patient was approached based on ED and RHC intake registries, with the exception of ED females. Given that KCMC's ED patient population is heavily male, to maintain enrollment goals, every female who presented to the ED was approached.~~

After the research assistants ensured patients were medically stable, they approached patients in a quiet, private location and ~~provided an introduction to~~introduced the study. All

Formatted: Font: 12 pt

Formatted: Normal, Indent: First line: 0.5"

Formatted: Font: 12 pt

Formatted: Font: (Default) Arial, 11 pt, Not Italic

patients had the opportunity to decline participation, but if willing to proceed, an in-depth discussion of the risks and benefits of study participation was provided. If agreeing, written consent was obtained, and survey questions were delivered~~elivered~~. All questions were administered~~asked~~ orally by a research team member to encourage participation from all literacy levels.

~~*Instruments*~~

Instruments and Variable Measurements

Formatted: Font: (Default) Times New Roman

The DrInC was used to quantitatively assess the impact alcohol use had on an individual. DrInC, which had been cross-culturally adapted and clinically tested previously at KCMC [43~~0~~], is a 50-item survey tool consisting of yes or no questions designed to assess alcohol-related consequences across five different domains [44~~1~~,45~~2~~]. These include physical, social responsibility, interpersonal, intrapersonal, and impulse control (Table 1). DrInC scores range from 0 to 50, with higher scores overall indicating more affirmative responses to various alcohol-related items as shown in Table 1 below. Of note, while DrInC helps to quantitatively illustrate the range of alcohol-related consequences one has experienced, it does not measure the severity or frequency of these consequences. Further, while the DrInC tool has been validated in the study setting [43], no ~~particular clinically~~clinically relevant cut-off score for this tool exists, in part ~~.~~because this is among the first studies to apply this tool among Kiswahili-speaking patients, but also because ideally individuals would not experience any alcohol-related consequences. To help account for this lack of cut-off score, DrInC scores were correlated with alcohol use as is detailed further below.

Table 1: Consequence Domains and Corresponding Items of the Drinkers' Inventory of Consequences

DrInC	Corresponding Items

Subcategories	
Physical	“I have had a hangover after drinking”<br>“After drinking, I have had trouble with sleeping, staying asleep, or nightmares”<br>“I have been sick and vomited after drinking”<br>“Because of my drinking, I have not eaten properly”<br>“My physical health has been harmed by my drinking”<br>“My physical appearance has been harmed by my drinking”<br>“My sex life has suffered because of my drinking”<br>“While drinking or intoxicated, I have been physically hurt, injured, or burned”
Social Responsibility	“I have missed days of work or school because of my drinking”<br>“The quality of my work has suffered because of my drinking”<br>“I have failed to do what is expected of me because of my drinking”<br>“I have gotten into trouble because of drinking”<br>“I have had money problems because of my drinking”<br>“I have spent too much or lost a lot of money because of my drinking”<br>“I have been suspended/fired from or left a job or school because of my drinking”
Interpersonal	“My family or friends have worried or complained about my drinking”<br>“My ability to be a good parent has been harmed by my drinking”<br>“While drinking, I have said or done embarrassing things”<br>“While drinking, I have said harsh or cruel things to someone”<br>“My marriage or love relationship has been harmed by my drinking”<br>“My family has been hurt by my drinking”<br>“A friendship or close relationship has been damaged by my drinking”<br>“My drinking has damaged my social life, popularity, or reputation”<br>“~~8~~I have lost a marriage or a close love relationship because of my drinking”<br>“I have lost a friend because of my drinking”
Intrapersonal	“I have felt bad about myself because of my drinking”<br>“I have been unhappy because of my drinking”<br>“I have felt guilty or ashamed because of my drinking”<br>“When drinking, my personality has changed for the worse”<br>“I have lost interest in activities and hobbies because of my drinking”<br>“My spiritual or moral life has been harmed by my drinking”<br>“Because of my drinking, I have not had the kind of life that I want”<br>“My drinking has gotten in the way of my growth as a person”
Impulse Control	“I have driven a motor vehicle after having three or more drinks”<br>“My drinking has caused me to use other drugs more”<br>“I have taken foolish risks when I have been drinking”<br>“When drinking, I have done impulsive things that I regretted later”<br>“I have gotten into a physical fight while drinking”<br>“I have smoked more when I am drinking”<br>“I have been overweight because of my drinking”<br>“I have been arrested for driving under the influence of alcohol”<br>“I have had trouble with the law (other than driving while intoxicated) because of my drinking”<br>“I have had an accident while drinking or intoxicated”

	"While drinking or intoxicated, I have injured someone else"<br>"I have broken things or damaged property while drinking or intoxicated"

Data Analysis Procedure

Overall and category specific DrInC scores for the three patient populations were assessed through descriptive frequencies and proportions. DrInC scores were also analyzed in relation to patients' alcohol intake to identify if an association between increased alcohol consumption and more consequences existed. As previously referenced, a~~A~~lcohol consumption was measured by ~~the Alcohol Use Disorder Identification Test (~~AUDIT~~)~~, a clinical tool used to identify patients with unhealthy drinking behaviors that was administered at the same time as the DrInC survey. AUDIT scores range from 0 to 40, with higher scores representing more hazardous alcohol consumption and scores of 8 or greater clinically indicative of risk for adverse alcohol-related health effects both globally and in the study setting [46~~3~~–49~~6~~]. The relationship between patient DrInC and AUDIT scores was measured through a linear regression analysis, the independent variable being DrInC scores and the dependent variable being AUDIT scores. Incomplete surveys were still included in ~~analyses~~the analyses. All quantitative data were analyzed in R Studio version 1.4.

Qualitative Data

Study Design

Using a purposive sampling strategy guided by preliminary quantitative data analyses, 19 individuals who had previously been enrolled in this study and completed all study questionnaires were contacted either at the end of their survey or later by phone call to gauge their interest in participating in a semi-structured IDI. All contacted patients agreed to

Formatted: Indent: First line: 0.5"

participate. IDIs were conducted at KCMC and later analyzed using a grounded theory methodology.

Formatted: Font: 12 pt, Not Italic

Sample Size Estimation

All participants who completed the quantitative survey were tentatively eligible for enrollment in the ~~proceeding~~ semi-structured interview. The study team aimed to enroll 20 IDI participants or until data saturation had been met [50] ~~and agreed upon by the entire research team. Further,~~ T~~t~~o maintain a representative sample of IDI participants, we aimed to enroll ten~~10~~ ED men, five~~5~~ ED women, and five~~5~~ RHC women for participation in the interview. For the purposes of this study, data saturation was defined as absence of new themes after three~~3~~ consecutive interviews for each subgroup (ED men, ED women, RHC women). IDI data collection was concluded at 19 interviews after data saturation had been met for the ED men IDI subgroup after 9 interviews.

Formatted: Indent: First line: 0.5"

Formatted: Font: (Default) Times New Roman

Sampling Technique

Nineteen out of the 676 patients enrolled were selected to participate in IDIs. Participants were chosen through purposive sampling to be balanced by gender, encompass diverse demographic backgrounds in terms of age, religion, income, profession, and marital status, and hold varying degrees of personal alcohol consumption. Additionally, the research team strove to have multiple unique experiences with alcohol use represented in the final data set, from patients who only reported neutral or positive past experiences with alcohol, used to drink but are now abstinent, have a close friend or relative that drinks heavily, or suffered significant negative consequences from alcohol. Interviewee characteristics were re-reviewed after two subsequent IDIs, and any needed changes were implemented when selecting the next IDI candidate.

Formatted: Indent: First line: 0.5"

Formatted: Font: (Default) Times New Roman, 12 pt, Not Italic

Data Collection and Instruments

Nineteen out of the 678 patients enrolled were selected to participate in IDIs. Participants were chosen through purposive sampling to be balanced by gender, encompass diverse demographic backgrounds in terms of age, religion, income, profession, and marital status, and hold varying degrees of personal alcohol consumption. Additionally, the research team strove to have multiple unique experiences with alcohol use represented in the final data set, from patients who only reported neutral or positive past experiences with alcohol, used to drink but are now abstinent, have a close friend or relative that drinks heavily, or suffered significant negative consequences from alcohol. Interviewee characteristics were re-reviewed after two subsequent IDIs, and any needed changes were implemented when selecting the next IDI candidate.

PThese patients were approached for IDI participation at the conclusion of their initial survey or in a later phone call. If the patient expressed interest in participating, the same-gendered research assistant who had originally enrolled them set up a time to meet and conducted the interview with them in a private room at KCMC. All interviews were conducted in Kiswahili using a semi-structured interview guide featuring open-ended questions and probes for situations where the information provided was unclear, conflicted with previous statements, or when a notable story warranted further explanation. and were recorded on a handheld audio recorder. The interview guide was piloted by research team members at KCMC to ensure interview relevancy and cultural and linguistic validity prior to the commencement of the interviews. With the consent of the participant, all IDIs were audio recorded using a handheld recording device for later transcription and translation. Lastly, The research assistant conducting the interview asked probing follow-up questions when information was unclear, conflicted with previous statements, or when a notable story warranted further explanation. IDI participants were

given a small stipend (5000 TSH, equivalent to approximately $2 USD) to reimburse their travels and were offered a snack and drink midway through.

Data Analysis Procedure

~~An applied thematic coding~~A ~~,~~ grounded theory approach, chosen as this topic remains poorly understood in the local context, was used to ~~inductively and deductively analyze~~analyze all qualitative data [51-53]. US and Tanzanian study team members co-developed the initial codebook from the first several IDIs. This codebook was inductively and deductively generated, using broad categories from the interview guide to provide a general framework for qualitative themes along with organic generation of codes without preconceived notions from the study team in concordance with grounded theory methodology[52-54]. ~~.~~ The themes established within the codebook facilitated the creation of content memos for each emerging theme and sub-theme which in turn provided the basis for our qualitative findings section below. The codebook remained an evolving document as new themes emerged throughout data collection.

Interviews were coded by both Tanzanian and US research team who first independently coded initial interviews and then met to discuss and resolve any coding discrepancies. This process was repeated until 80% agreement was reached. At that point, with approval from the research team, the lead analyst completed the coding of all remaining interviews, updating the codebook accordingly. After the preliminary quantitative analyses, qualitative and quantitative data collection occurred in parallel for the remainder of the study period.~~Iterative development of the initial codebook was~~as ~~led by Tanzanian and US team members~~ and ~~, facilitated the creation of content memos per each emerging theme and sub-theme~~code~~. These content memos then served as the foundation for the final analysis~~ of all interviews ~~after discussion and feedback~~

~~with the full research team~~. All qualitative analysis was conducted in NVivo software version 12 and approved by the entire research team prior to the reporting of these results.

Ethics Statement

Ethical approval for this study was obtained from the Duke University Institutional Review Board, the Kilimanjaro Christian Medical University College Ethical Review Board, and the Tanzanian National Institute of Medical Research prior to any data collection. Personal health information was employed in screening and enrollment procedures, but was de-identified when collected, stored, and analyzed and shared only via a data share agreement.

Results

Quantitative

Six-hundred and seventy-six~~eight~~ patients started and ~~but only~~ 655 patients completed the~~our~~ survey questionnaire. ~~, w~~The average age of all ED and RHC participants was 41.7 years old (Table 2). 80% of all participants identified with the Christian religion, 50% reported being married, and 57% reported being employed at the time of survey delivery. Additional reporting of stratified participant demographics can be found in Table 2. ~~here ED m~~Men attending the ED were found to spend the most money on alcohol per week, drink the most frequently and in the largest quantities, and have the highest average AUDIT scores as compared to women ED and RHC patients [42~~39~~]. In comparing alcohol consumption with alcohol-related consequences, AUDIT and DrInC scores were found to be linearly associated across all three patient populations (Figure 1). This association was strongest for women attending the ED ~~patients~~ (R^2 = 0.76; p<0.001) and weakest for men ED patients (R^2 = 0.65; p<0.001) and women RHC patients (R^2 = 0.53; p<0.001).

For alcohol-related consequences, ~~ED~~ men attending the ED were found to have the highest average [SD] DrInC scores (16.4 [19.6]), followed by ED women (9.11 [13.1]), and RHC women (5.47 [9.33]) (Table 2). This held true for DrInC subscores, where across every category except interpersonal, ED men had the highest, ED women had the second highest, and RHC women had the lowest average DrInC scores (Table 3~~2~~; Figure 2). In the interpersonal subscore, the difference in average DrInC scores was not statistically significant. However, in all subcategories except social responsibility and impulse control, ED women had the highest range of DrInC scores (Figure 2).

Table 2: Study Population Demographics

Demographics by Population Type	Overall, N = 655[1]	ED Women, N = 271[1]	RHC Women, N = 270[1]	ED Men, N = 114[1]	P Value
**Age Category**					<0.001
18 to 24	110 (18%)	47 (19%)	53 (21%)	10 (9.5%)	
25 to 34	159 (26%)	41 (16%)	92 (37%)	26 (25%)	
35 to 44	106 (17%)	42 (17%)	45 (18%)	19 (18%)	
44 to 54	94 (16%)	47 (19%)	32 (13%)	15 (14%)	
Over 55	137 (23%)	75 (30%)	27 (11%)	35 (33%)	
Missing/refused per block	49 / 655	19 / 271	21 / 270	9 / 114	
**Average Age**	41.7 (18.7)	46.1 (22.8)	36.4 (12.7)	45.2 (16.4)	0.101
**Personal Income Category (TZS per month)**					0.001
0 to 50,000	205 (32%)	110 (41%)	68 (25%)	27 (25%)	
50,001 to 100,000	44 (6.8%	17 (6.3%)	15 (5.6%)	12 (11%)	
100,001 to 150,000	56 (8.7%)	17 (6.3%)	29 (11%)	10 (9.4%)	
150,001 to 200,000	91 (14%)	34 (13%)	37 (14%)	20 (19%)	
> 200,000	248 (39%)	92 (34%)	119 (44%)	37 (35%)	
Missing/refused per block	11 / 655	1 / 271	2 / 270	8 / 114	
**Religion**					0.187
None	11 (1.7%)	5 (1.8%)	3 (1.1%)	3 (2.6%)	

Demographics by Population Type	Overall, N = 655[1]	ED Women, N = 271[1]	RHC Women, N = 270[1]	ED Men, N = 114[1]	P Value
Christian	522 (80%)	218 (80%)	222 (82%)	82 (72%)	
Muslim	121 (18%)	47 (17%)	45 (17%)	29 (25%)	
Other	1 (0.2%)	1 (0.4%)	0 (0%)	0 (0%)	
Missing/refused per block	0 / 655	0 / 271	0 / 270	0 / 114	
**Highest Educational Attainment**					<0.001
None	52 (8.7%)	30 (12%)	7 (2.9%)	15 (13%)	
Primary	182 (31%)	82 (34%)	61 (25%)	39 (35%)	
Secondary	133 (22%)	48 (20%)	64 (27%)	21 (19%)	
College	170 (29%)	65 (27%)	87 (36%)	18 (16%)	
Graduate	13 (2.2%)	4 (1.7%)	2 (0.8%)	7 (6.2%)	
Vocational	45 (7.6%)	13 (5.4%)	20 (8.3%)	12 (11%)	
Missing/refused	60 / 655	29 / 271	29 / 270	2 / 112	
**Marital Status**					0.013
Never Married or Single	135 (21%)	55 (20%)	56 (21%)	24 (21%)	
Living with a partner, not in a registered marriage	79 (12%)	26 (9.6%)	41 (15%)	12 (11%)	
Living with a partner in a registered marriage	327 (50%)	128 (47%)	140 (52%)	59 (52%)	

Demographics by Population Type	Overall, N = 655[1]	ED Women, N = 271[1]	RHC Women, N = 270[1]	ED Men, N = 114[1]	P Value
Divorced or Separated	3 (4.7%)	16 (5.9%)	6 (2.2%)	9 (8.0%)	
Widowed	81 (12%)	46 (17%)	27 (10%)	8 (7.1%)	
Missing/refused	2 / 655	0 / 271	0 / 270	2 / 114	
**Employment Status**					<0.001
Employed	371 (57%)	127 (47%)	192 (71%)	52 (46%)	
Unemployed	215 (33%)	111 (41%)	50 (19%)	54 (47%)	
Student	69 (11%)	33 (12%)	28 (10%)	8 (7.0%)	
Missing/refused	0 / 655	0 / 271	0 / 270	0 / 114	
**Tribe**					0.070
Chagga	329 (50%)	126 (46%)	146 (54%)	57 (50%)	
Iraq	21 (3.2%)	9 (3.3%)	8 (3.0%)	4 (3.5%)	
Maasai	25 (3.8%)	13 (4.8%)	7 (2.6%)	5 (4.4%)	
Mmeru	29 (4.4%)	14 (5.2%)	11 (4.1%)	4 (3.5%)	
Muha or Non-African	6 (1.0%)	3 (1.1%)	3 (1.1%)	0 (0%)	
Nyaturu	17 (2.6%)	7 (2.6%)	10 (3.7%)	0 (0%)	
Pare	70 (11%)	27 (10.0%)	25 (9.3%)	18 (16%)	
Sambaa	26 (4.0%)	7 (2.6%)	10 (3.7%)	9 (7.9%)	
Sukuma	34 (5.2%)	10 (3.7%)	16 (5.9%)	8 (7.0%)	

Demographics by Population Type	Overall, N = 655[1]	ED Women, N = 271[1]	RHC Women, N = 270[1]	ED Men, N = 114[1]	P Value
Other African	98 (15%)	55 (20%)	34 (13%)	9 (7.9%)	
Missing/refused	0 / 655	0 / 271	0 / 270	0 / 114	

[1]n / N (%); Mean (SD)

Figure 1: Association of AUDIT and DrInC Scores across Patient Populations

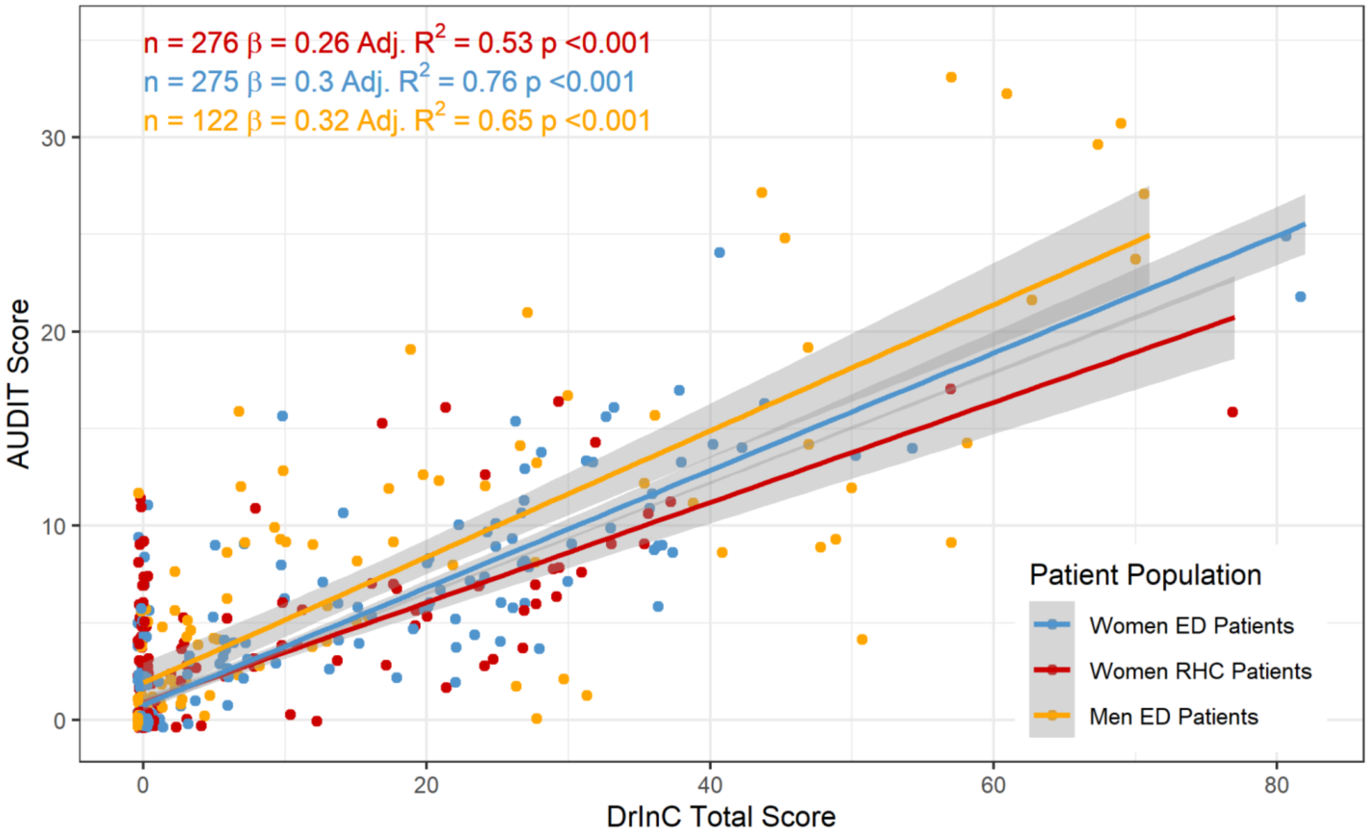

Table 32: Reported Average DrInC Scores and Subscores across Patient Populations

	RHC Women (n =270)	ED Women (n = 271)	ED Men (n = 114)	Total Population (n = 655)
**Overall DrInC Score,** *missing: 17*	5.47 (9.33, 75)	9.11 (13.1, 80)	16.4 (19.6, 88)	9.00 (13.9,)
**Physical Subscore,** *missing: 17*	0.60598 (1.73, 12)	1.39 (2.75, 15)	2.54 (3.37, 12)	1.28 (2.62, 15)
**Intrapersonal Subscore,** *missing: 17*	0.542 (1.87, 14)	1.000.996 (2.34, 20)	2.86 (3.97, 17)	1.15 (2.68, 20)
**Social Responsibility Subscore,** *missing: 17*	0.5217 (1.74, 13)	1.15 (2.53, 14)	2.53 (3.77, 17)	1.15 (2.66, 17)
**Interpersonal Subscore,** *missing: 17*	0.391 (1.51, 14)	0.993 (2.67, 23)	3.02 (4.42, 17)	1.12 (2.91, 23)
**Impulse Control Subscore,** *missing: 17*	0.3436 (1.35, 17)	0.70695 (1.89, 13)	1.99 (3.34, 17)	0.80796 (2.17, 17)
Data are presented as mean (SD, range) for quantitative variables, and count (%) for qualitative variables.				

Figure 2: Box-Plot Distribution of DrInC Scores and Subscores across Patient Populations

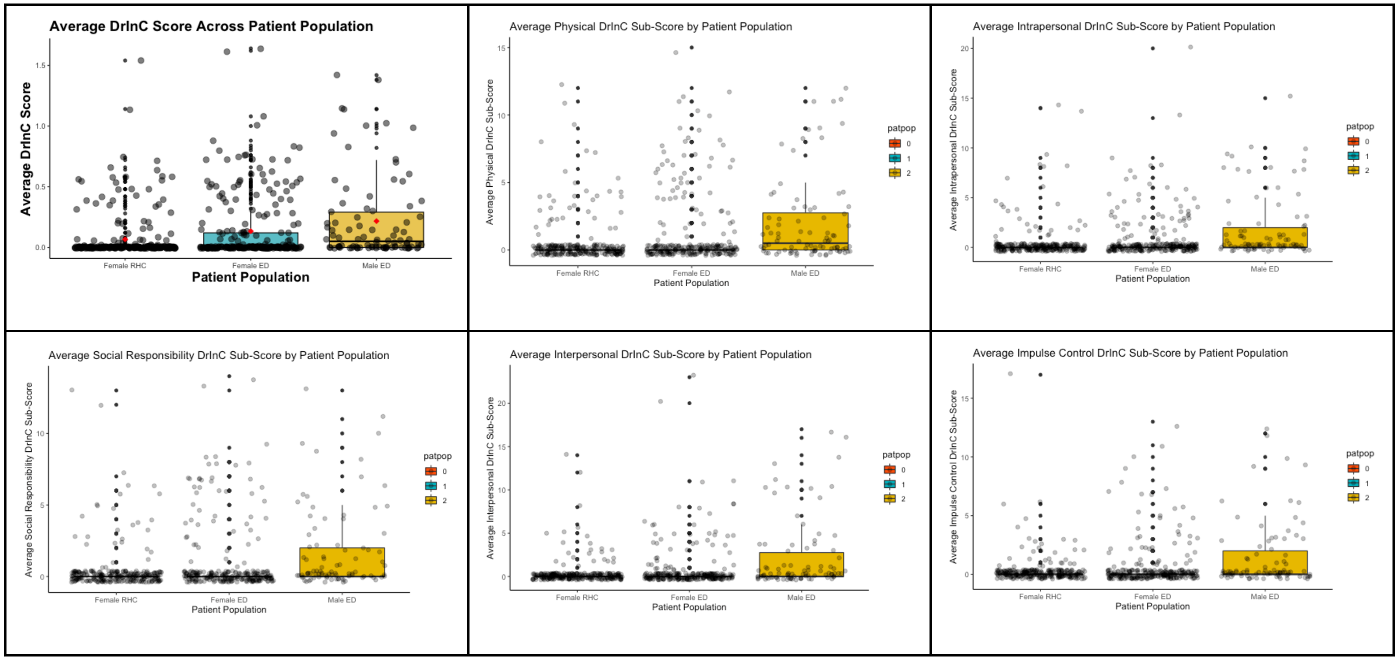

Qualitative

Nineteen individuals (RHC women, n = 5; ED women, n = 5; ED men, n = 9) participated in IDIs (Table 3). The ages of IDI participants spanned from 20 to 70 years, with a mix of education, income, and alcohol intake levels represented.

Formatted: Line spacing: Double

Table 3: Interviewee Demographics

Demographics by Population Type	Overall, N = 19[1]	ED Women, N = 5[1]	RHC Women, N = 5[1]	ED Men, N = 9[1]
**Age Category**				
18 to 24	4 / 19 (21.1%)	2 / 5 (40%)	2 / 5 (40%)	0 / 9 (0%)
25 to 34	4 / 19 (21.1%)	1 / 5 (20%)	0 / 5 (0%)	3 / 9 (33.3%)
35 to 44	5 / 19 (26.3%)	1 / 5 (20%)	2 / 5 (40%)	2 / 9 (22.2%)
44 to 54	4 / 19 (21.1%)	1 / 5 (20%)	1 / 5 (20%)	2 / 9 (22.2%)
Over 55	2 / 19 (10.5%)	0 / 5 (0%)	0 / 5 (0%)	2 / 9 (22.2%)
**Personal Income (TZS), *missing: 7***	282500 (422807.18)	360000 (505129.05)	268000 (422807.18)	125000 (106066.02)
**Household Income (TZS), *missing: 7***	516666.67 (484455.33)	730000 (582329.07)	390000 (484455.33)	300000 (141421.356)
**Religion**				
Christian	15 / 19 (78.9%)	4 / 5 (80%)	5 / 5 (100%)	6 / 9 (66.7%)
Muslim	4 / 19 (21.1%)	1 / 5 (20%)	0 / 5 (0 %)	3 / 9 (33.3%)
**Highest Educational Attainment, *missing: 2***				
College	9 / 19 (47.4%)	3 / 5 (60%)	3 / 5 (60%)	3 / 9 (33.3%)
Graduate	1 / 19 (5.3%)	0 / 5 (0%)	0 / 5 (0%)	1 / 9 (11.1%)
None	0 / 19 (0%)	0 / 5 (0%)	0 / 5 (0%)	0 / 9 (0%)
Primary	5 / 19 (26.3%)	1 / 5 (20%)	1 / 5 (20%)	3 / 9 (33.3%)
Secondary	2 / 19 (10.5%)	1 / 5 (20%)	1 / 5 (20%)	0 / 9 (0%)
Vocational	0 / 19 (0%)	0 / 5 (0%)	0 / 5 (0%)	0 / 9 (0%)
**Marital Status**				
Divorced or Separated	2 / 19 (10.5%)	1 / 5 (20%)	0 / 5 (0%)	1 / 9 (11.1%)
Living together with a partner but not in a registered marriage	2 / 19 (10.5%)	0 / 5 (0%)	1 / 5 (20%)	1 / 9 (11.1%)
Living together with a partner in a registered marriage	9 / 19 (47.4%)	2 / 5 (40%)	2 / 5 (40%)	5 / 9 (55.6%)
Never Married or Single	5 / 19 (26.3%)	2 / 5 (40%)	2 / 5 (40%)	1 / 9 (11.1%)
Widowed	1 / 19 (5.3%)	0 / 5 (0%)	0 / 5 (0%)	1 / 9 (11.1%)

Demographics by Population Type	Overall, N = 19[1]	ED Women, N = 5[1]	RHC Women, N = 5[1]	ED Men, N = 9[1]
**Employment Status**				
Employed	12 / 19 (63.2)	3 / 5 (60%)	5 / 5 (100%)	4 / 9 (44.4%)
Unemployed & not a Student	4 / 19 (21.1%)	0 / 5 (0%)	0 / 5 (0%)	4 / 9 (44.4%)
Unemployed but Student	3 / 19 (15.8%)	2 / 5 (40%)	0 / 5 (0%)	1 / 9 (11.1%)
**Tribe**				
Chagga	9 / 19 (47.4%)	2 / 5 (40%)	4 / 5 (80%)	3 / 9 (33.3%)
Iraq	1 / 19 (5.3%)	0 / 5 (0%)	0 / 5 (0%)	1 / 9 (11.1%)
Maasai	1 / 19 (5.3%)	0 / 5 (0%)	0 / 5 (0%)	1 / 9 (11.1%)
Mmeru	2 / 19 (10.5%)	0 / 5 (0%)	0 / 5 (0%)	2 / 9 (22.2%)
Nyaturu	1 / 19 (5.3%)	1 / 5 (20%)	0 / 5 (0%)	0 / 9 (0%)
Other African	3 / 19 (15.8)	2 / 5 (40%)	1 / 5 (20%)	0 / 9 (0%)
Pare	1 / 19 (5.3%)	0 / 5 (0%)	0 / 5 (0%)	1 / 9 (11.1%)
Sambaa	1 / 19 (5.3%)	0 / 5 (0%)	0 / 5 (0%)	1 / 9 (11.1%)
**Drinking Frequency**				
0 times/week	5 / 19 (26.3%)	1 / 5 (20%)	1 / 5 (20%)	3 / 9 (33.3%)
1-2 times/week	6 / 19 (31.6%)	1 / 5 (20%)	1 / 5 (20%)	4 / 9 (44.4%)
3-4 times/week	7 /19 (36.8%)	2 / 5 (40%)	3 / 5 (60%)	2 / 9 (22.2%)
5-6 times/week	1 / 19 (5.3%)	0 / 5 (0%)	0 / 5 (0%)	1 / 9 (11.1%)
Every day	0 / 19 (0%)	0 / 5 (0%)	0 / 5 (0%)	0 / 9 (0%)
Multiple times a day	0 / 19 (0%)	0 / 5 (0%)	0 / 5 (0%)	0 / 9 (0%)
**Drinking Quantity**				
>6 bottles	0 / 19 (0%)	0 / 5 (0%)	0 / 5 (0%)	0 / 9 (0%)
0 drinks	4 / 19 (21.1%)	1 / 5 (20%)	1 / 5 (20%)	2 / 9 (22.2%)
1-2 bottles	8 / 19 (42.1%)	2 / 5 (40%)	3 / 5 (60%)	3 / 9 (33.3%)
3-4 bottles	6 / 19 (31.6%)	2 / 5 (40%)	1 / 5 (20%)	3 / 9 (33.3%)
5-6 bottles	0 / 19 (0%)	0 / 5 (0%)	0 / 5 (0%)	0 / 9 (0%)
>6 bottles	0 / 19 (0%)	0 / 5 (0%)	0 / 5 (0%)	0 / 9 (0%)
Refused/Do not know	1 / 19 (5.3%)	0 / 5 (0%)	0 / 5 (0%)	1 / 9 (11.1%)

[1]n / N (%); Mean (SD)

Approximately a third of participants held positive views or remarked that they generally benefited from alcohol, almost half said these views and experiences were overall negative, and the remaining quarter of participants stated that it depended. Two main themes and several sub-themes related to community perceptions and the impact of alcohol emerged from the IDIs. The two main themes were Alcohol's perceived harms and Alcohol's perceived benefits (Table 4). Participants noted the ways they felt alcohol harmed either them or their community, which

included a failure to fulfill their responsibilities, stigma, sexual assault, increased conflict, chronic health issues, and financial instability. On the other hand, ~~several~~ examples of how alcohol was viewed~~seen~~ as beneficial within the community were mentioned, including fostering economic growth, creating an outlet to uphold cultural traditions, and instilling more social unity.

Table 4: Qualitative Themes and Sub-Themes

**Themes**	**Sub-Themes**
Alcohol’s Perceived Harms	Inability to Fulfill Familial Responsibilities<br>Stigma and Discrimination<br>Sexual Consequences<br>Interpersonal Conflict and Violence<br>Injuries and Chronic Disease<br>Financial Instability
Alcohol’s Perceived Benefits	Economic Growth<br>Upholding Cultural Practices<br>Social Unity

To preface this data, the communities' perceptions and expectations of alcohol use explored in this results section is the amalgamation of general themes and trends from 19 participants. While these findings have been grouped for clarity, it is important to note that there was a significant amount of variability in responses and opinions, especially across gender, “*various religions, clans, and tribes” (IDI #3, Female)*. As one participant explains:

“*The community is the combination of many people with different ideologies about life; there is no way that my perception will be the same with everybody in the community. It’s because we have been born and raised in different families and environments and everyone came up with his/her own life basics, so according to how one came across alcohol it is how the perception will be built~~.~~.” (IDI #19, Female)*

Alcohol's Perceived Harms

As part of their interview, IDI participants were asked how alcohol use negatively impacted their community. Respondents stated that alcohol leads to stigma, sexual violence, risky sexual behaviors, and an inability of community members to uphold individual responsibilities. Most also saw it as an instigator of physical, verbal, and emotional conflict among family members, and believed it contributed to long-term health issues and financial instability.

> *"What I can say is the society do not have positive expectation about alcohol use, because we see families disintegrated, people fall sick as a result of alcohol and many people at first drink but are able to work and carry on their daily activities and routine but later on they fail to carry on tasks because alcohol makes them weak." (IDI #3, Female)*

<u>Inability to Fulfill Family Responsibilities</u>

Most participants mentioned that alcohol was an inhibitor in one's ability to fulfill their responsibilities to their family. Those who drank in excess would "*forget [their] responsibilities*" (IDI #11, Male), become too "*weak*" (IDI #3, Female), "*lazy*" (IDI #19, Female), or simply, "*fail to perform their daily duties*" (IDI #6, Female). One participant elaborates on their own experience:

> *"I see alcohol is not such a good thing…It can make one forgetting everything about his/her family, even children can go to bed hungry without you having an idea because you're busy drinking. Children left home by drunkard parents can even experience assaults of different kinds just because the parents are not there to protect them…I advice*

my fellow parents and those who have families to care for to stop taking alcohol" (IDI #7, Female)

<u>Stigma and Discrimination</u>

All interviewees noted alcohol-related stigma to be present to some extent in their communities, but to varying degrees and concentrated in certain circumstances. One participant reported that they "*haven't seen any stigma among men or women…unless if after drinking you cause problems like fighting or abusing other people*" (IDI#12, Male). As another explains, stigma could also arise when one fails to accomplish preset responsibilities or drinks unsafe alcoholic beverages:

"*Alcohol intake in Tanzania is like a ritual. Anyone can drink. The stigma arises when someone fails to control their drinking habit, like when someone drinks all the time and fails to fulfill his or her obligations, or if someone drinks those homemade beers (local brews) that are not tested and not standardized by the bureau of standards. People who drink Gongo and Dadii are really stigmatized in the community. Because once you drink those, you may even fail to control your bowels, which leads to shame and embarrassment*" (IDI #2, Male).

In addition to the quality of liquor, another notable facet of alcohol-related stigma is its interaction with gender. Participants noted that "*women who drink are stigmatized more than men*" (IDI #7, Female) and that their "*community strongly hate women who drinks alcohol"* (IDI #19, Male). This arose in part because of aforementioned gender roles, where because "*women stay with the children most of the time"* (IDI #19, Male) there is concern that their alcohol habits will have greater negative repercussions on their children.

<u>Sexual Consequences</u>

Interviewees reported that alcohol use caused or contributed to significant sexual consequences uniquely experienced by each gender. The first of these was the increased risk of acquiring a sexually transmitted disease – *"when you drink too much it can put you a risky of even practice unsafe sexual intercourse"* (IDI #4, Male), which generally stemmed from having more sexual partners:

> *"Men who drink lacks discipline, they don't respect themselves or their wives, they engage in sexual immorality without considering the fact that they are aged, married and with families."* (IDI #7, Female)

A couple of men and women participants noted that alcohol could hinder their ability to perform sexual acts –

> *"A person cannot perform sexual activity well as before, to men they may encounter erectile dysfunction and women may also not be able to involve and perform sexual activity properly. Alcohol can make a person really desire the sexual intimacy but just not able to perform it because of the effect of alcohol in the brain."* (IDI #16, Female)

This, though, was only mentioned by a few participants and stands in sharp contrast to the common theme of alcohol-related sexual assault. This final sexual consequence was described almost exclusively as men being violent against women – "*there is someone I know she was invited to a man house to drink alcohol it was her first time to drink alcohol she got drunk the man raped her*" (IDI #9, Female). Participants disagreed on whether or not men incurred sexual assault when drinking, one saying *"men…cannot experience sexual violence because it is not a common practice in Tanzania"* (IDI #2, Male), while another reported that *"men…when they are drunk, they have been sexually abused by the fellow men"* (IDI #13, Female).

<u>Interpersonal Conflict and Violence</u>

Sexual assault was not the only product of the increased violent and aggressive behavior that alcohol use incited. Interpersonal conflict and violence was a theme noted by three-quarters of participants. IDI #19 states,

> "*Alcohol causes misunderstandings and fights in society, I have witnessed many times that people get drunk and start to say abusive words, become very aggressive and arrogant.*" (IDI #19, Female)

Respondents described how alcohol use instigates emotional and physical conflict and distress within the family – "*I know many families that when the father is drunk, the mother and children become so anxious and afraid to sit with him closely. The most common violence is physical and emotional abuse*" (IDI #17, Male). As seen in this and the next quote, aggression was described as being more common in men – *"it's very difficult to see women fighting after drinking. But men are always aggressive and violent"* (IDI #3, Female), with the ensuing conflict leading to family disintegration.

Moreover, several participants (4 of the 19 participants) discuss how a child's mental health is significantly affected by witnessing their parents "*fighting and shouting*" (INT#12, Male), a form of emotional conflict, due to alcohol consumption. As a result, many pre-existing interpersonal relationships were harmed or broken – *"I have witnessed many times that people get drunk and start to say abusive words to one another ...I can say that alcohol can truly bring people together but can also destroy people's relationship"* (IDI #19, Female).

Injuries and Chronic Disease

While all the categories of harms thus far discussed can lead to significant physical, mental, and emotional problems, a plethora of other long-term health issues stemming from

alcohol use like injuries and road traffic incidents, illness and chronic disease were also discussed by most participants.

> *"Alcohol can lead to organ damage like liver so there is a lot of complications...I had a friend of mine who was drinking every day, and he was not eating well so he ends up dying. Because of alcohol intoxication. But before that he was having anemia (low blood), low body weight, and muscle wasting" (IDI #12, Male)*

Health issues that were attributed to alcohol use by participants included the following: liver cirrhosis, cancer, kidney failure, "*puffy cheek*" (water retention resulting from alcohol use), neurological insults, and even death. Some participants even recount how local brews —which are "*not tested and not safe*" *(IDI #4, Male)* and have been characterized as "*strong alcohol*" *(IDI #8, Male)*— may incite even greater health consequences in regular consumers.

Financial Instability

Lastly, almost half of the participants mentioned economic loss and financial instability, encouraged via a variety of different mechanisms, as a major source of personal and community harm. First, participants proposed that economic loss and financial instability seem to arise from the perceived difficulty of drinkers to find or maintain employment. That is, participants agreed that employed individuals who drink experience a loss of productivity. IDI #17, for example, stated, *"Alcohol-related behaviors may lower the individual performance at (the) workplace which leads to inadequate or inefficient production" (IDI #17, Male).*

In addition, if an individual spends *"a lot of money buying alcohol"* (IDI #12, Male) for themselves, this can take a toll on personal savings –*"I lost my business because of drinking too much"* (IDI #12, Male). One participant describes the monetary loss alcohol-related spending could cause:

"*Alcohol affected my uncle so badly, he was so rich, and helped people in his family and relatives but once he became addicted with alcohol everything turns to zero. He lost everything, even closer friends. He is unable to take care of his family even paying school fees for his children!" (IDI# 5, Male)*

As men especially were seen to be the primary breadwinners for their families, a father's excess spending on alcohol could "*decrease in family income"* (IDI #17, Male). This created negative repercussions for a man's wife and children and permanently cripple their financial holdings:

"Alcohol is a reason for many families here to fall into extreme poverty because of men as family providers spend a lot of resources in alcohol while they just earn little to help their families survive as most of them work as drivers of tricycles and motorcycles. So, when a man has no control of his drinking habits and spends all of his income into drinking and definitely the family get into a vicious cycle of poverty." (INT #19)

While increased alcohol consumption was seen as contributing to personal or familial financial distress for some, as will be discussed in the next section, several participants noted a financial benefit to increased alcohol use for sellers or the government.

Alcohol's Perceived Benefits

When asked how alcohol has positively impacted their community, a third (only one of which was female) commented on the economic gain in alcohol production and sales, and half of the participants commonly mentioned social and/or cultural benefits.

<u>Economic Growth</u>

In contrast to the last sub-theme presented, IDI #2 described the monetary benefit alcohol brought to his community, saying it

*"contributes to the economic growth of an individual who is selling, a family or a national income. The government collects taxes from the brewing industry, which helps to increase national income" (*IDI# 2, Male*).*

Interestingly though, while the government benefited financially from alcohol sales, several respondents discussed how *"local brew"* (IDI #4, Male) is often not effectively tested for alcohol content levels and results in harmful consequences for their community. One participant explained this lack of government monitoring:

"The community views [alcohol] as a burden promoted by the government, it is the government that legalized it and allow people to manufacture alcohol. And sometimes the government fails to monitor these industries and see what kind of alcohol they are producing. It is very unfortunate that some of the businessmen are not faithful and manufacture alcohol which are very strong and damage people's brain." (IDI #12, Male)

Importantly, while the production of alcohol was seen to bring economic benefit for some, even this perceived boon carried with it the potential for harm to community members.

Upholding Cultural Practices

As mentioned in the first quote in this manuscript, alcohol use was tied to cultural and tribal events – *"Many people consider alcohol to be part of their social culture and way of life"* (IDI #2, Male). Some respondents found it appropriate to consume alcohol at cultural events or special occasions, using alcohol during rituals as a way to "*signify respect and honor to our ancestors*" *(IDI #2, Male)* and "*honor their ancestors' way of living, social customs that have*

been practiced for generations and generations" *(IDI #3, Female)*. One woman summarized these sentiments:

> *"There are social events that cannot be done without alcohol, for example, if you go to pay for dowry you have to bring alcohol to the event and even in some rituals, they must brew alcohol during the ritual service. So, there's a positive way alcohol affects our society as a whole…many people drink alcohol to spend time together and enjoy" (IDI #9, Female)*

<u>Social Unity</u>

Finally, alcohol's positive influence on the social aspect of community life was a common theme in IDIs – *"alcohol helps people interact and have time to discuss different issues…it strengthens unity among people" (IDI #4, Male)* and

> *"[Alcohol] helps to bring people together in unity. You may find friends or just random people sitting together, making stories about a thing or two and celebrating their achievement over a glass of beer. In my society, you cannot just gather around grown-up people to discuss about something and just give them soda or juice, you must offer them alcohol so that they will listen to you properly"(IDI #19, Female).*

> *"The good results of alcohol, to me personally it has made my social life very great and I have been meeting a lot of people when I go for a drink, for my friends as we enjoy drinks together and talk about our lives but I have also met important and influential people to discuss important matters in business and work as we enjoy 1 or 2 glass of beer" (IDI #10, Female)*

Not only was alcohol use noted as bringing people together, but alcohol was also mentioned as having a key role in resolving social conflict and social gatherings:

"If you have misunderstandings with friends or family members, and you want to resolve those disputes…you need to offer them alcohol while you talk! You can't offer them soft drinks! Even when you want to get married, you need to buy a lot of alcohol for elderly people to drink and enjoy" (IDI #2, Male).

Discussion

This paper explored the perceptions of alcohol-related consequences through the lens of KCMC ED and RHC patients. This analysis is the first of its kind to provide a holistic overview of the harms and benefits of alcohol as perceived by patients in Moshi, Tanzania. Existing literature on Moshi has described the scope of alcohol use in this region [26,28,32,5547], examined the determinants of use [5648], and explored specific consequences related to alcohol, such as drunk driving [5749] and stigma [29,33]. Perceptions of alcohol use have been explored [23], but without looking at alcohol-related consequences and exclusively from the perspectives of injury patients. Our paper takes the existing data one step further by providing a comprehensive view of perceived alcohol-related impacts on individuals, their families, and their communities to allow for a clearer picture of alcohol's role in this context. Our results show that individuals and their families experienced alcohol-related social harms like stigma and interpersonal conflict; however, alcohol was also thought to foster unity at the broader community level. From a social dimension, while patients perceived alcohol to have some level of financial benefit on the community and government level through sales and taxes, substantial economic harm from excess spending on alcohol arose for the individuals and families of heavy

Formatted: Font: (Default) Times New Roman, Italic

Formatted: Heading 1, Line spacing: single

users. Finally, no health benefits were linked to alcohol consumption, but a slew of physical harms were mentioned by participants.

In this analysis, we found that participants saw both positive and negative impacts of alcohol use at the individual, interpersonal, and community levels, including the perceived benefit of social unity and upholding traditional customs contrasted by the harm of greater conflict and stigma. One ~~notable~~ stipulation to these results~~, though,~~ is that while male ED patients~~ED men~~ had the highest average scores on the DrInC interpersonal~~social responsibility~~ subscale~~ category~~, stigma was found to more severely affect women in our qualitative findings. Looking at the DrInC scale, on average, men responded affirmatively to experiencing approximately three different types of interpersonal consequences related to alcohol use, compared to 0.39 for female RHC patients and one for female ED patients. While these social harms were well documented, social benefits could also be seen as participants mentioned alcohol brought people together and fostered a sense of social unity. These mixed-methods findings, when taken together, demonstrate that the social ramifications of alcohol use are significant but nuanced, and vary by gender. Men, for example, generally experience a wider array of social stipulations, whereas women experience consequences that are more severe. ~~Looking first at the social consequences to the individual, p~~

Previous Moshi-based studies have identified alcohol-related stigma [33,58~~0~~] and a disproportionate stigma against women [29], however greater exploration on this topic is needed as these data sets were limited to ED injury patients. At the interpersonal level, alcohol use was purported to foster social connections but at the same time had the potential to generate conflict, going as far as to destroy romantic and family relationships. Though the first of these findings is not well-supported by academic literature, alcohol has been previously noted to be a mediator in

conflict and intimate partner violence both globally and regionally [591–6254]. While both social benefits and harms were noted by participants, only the latter is currently supported in scholarly publications. However, further research aimed at understanding the perceived positive associations of alcohol use can help better contextualize its ubiquitous usage in Moshi and pinpoint focus areas for future alcohol-reduction-focused interventions.

Similar to the social implications of alcohol use, we found that alcohol consumption can have important financial consequences at the individual and country levels. IDI participants described the a decreased household income that arose from excessive spending on alcohol. This echoes This is supported by other previously published study data where we found nearly 40% of male ED patients spent between 10 - 46% of overall average monthly household income on alcohol alone [4239,6355]. This current analysis further supports a gendered component to alcohol-related financial consequences. In our survey data, men had a higher positive response rate to questions like ‘I have missed days or work’ and ‘I have spent too much’ as encapsulated by DrInC’s social responsibility subscale. While women are spending the least amount on alcohol overall, previous data reportshowed that women consume less alcohol but also experience limited autonomy when it comes to the use of household funds. Other important economic-based findings from this analysis are the impacts at broader community and government levels, the effects of which were noted by participants to be overall positive. IDI participants believed that high rates of consumption were advantageous for sellers of alcohol and rising national excise taxes on alcohol products contributed to increasing revenue for the government of Tanzania. However, specifics of how this additional revenue was used to benefit Tanzanians were not mentioned.

Currently, while there is a lack of literature outlining the broader financial implications of alcohol use within the East African context. Matzopoulos et al. estimate that within South Africa, the tangible and intangible costs incurred from harmful alcohol consumption comprise between 10-12% of the country's total GDP and outweigh the economic benefits associated with alcohol taxes and sales [64~~56~~]. Further, research has suggested the inverse relationship between alcohol consumption and labor force participation globally [65~~57~~]. This contributes to larger macroeconomic implications, including losses in economic productivity and development. Overall, our data suggests that financial benefits from alcohol use were perceived to only exist for sellers of alcohol and at the government level. For individuals and the broader community, participants almost exclusively noted the negative monetary impacts of alcohol use. Based off these results and the scarce regional data, we suggest that future research has the opportunity to examine the micro- and macroeconomic implications of alcohol to inform policy recommendations in Tanzania.

Along with the social and financial consequences of alcohol consumption, we noted several physical harms stemming from alcohol use. Male ED patients~~ED men~~ were seen to experience a wider ~~more diverse~~ range of physical consequences than women presenting at the ED and RHC ~~ED and RHC women~~ as illustrated by our DrInC physical harm scores. Men answered positively to experiencing 2.54 alcohol-related physical harms compared to 0.60 from female RHC patients and 1.39 female ED patients. IDI data provided more information on the types of physical harm thought to be suffered by this patient population. ~~suggested that~~ ~~We found in IDI data that~~ ~~perceived alcohol-related physical harms~~ These listed harms included increases in sexual assault, violence, risky sexual behaviors, and chronic health issues, with no mention of any physical benefit to themselves or their communities. This last finding echoes a recent

statement made by the World Health Organization (WHO) stating that any level of drinking is harmful to physical health [66~~58~~,67~~59~~]. ~~Elaborating on these harms, t~~ While this statement and our qualitative data reports no physical benefits with alcohol use, past research has indicated that moderate alcohol intake can have positive circulatory and cardiovascular effects as well as being protective against the incidence of type 2 diabetes. The physical consequences of alcohol use ~~mentioned by our IDI~~noted by participants have been ~~noted~~ seen in other Africa-based studies. In sub-Saharan Africa, harmful alcohol use has been previously associated with increased road traffic injuries, drunk driving, and other injurious behaviors [32,57~~49~~,68~~0~~,69~~1~~]. Similar to our results, this past research has found a higher prevalence of alcohol-related physical harm in men. In addition, studies in Uganda and South Africa have found an association between sexual and intimate partner violence and alcohol consumption [70~~62~~,71~~63~~]. Last, our IDI data also echoes the WHO and the Center for Disease Control's growing concern over the chronic health consequences of excess consumption such as liver and kidney failure [72~~64~~]. Effective alcohol-reduction programs stand to reduce not only the~~se~~ negative health effects associated with alcohol but also violent behaviors that incite injuries. For example, Walton et al. and Ward et al. found, respectively, that interventions aiming to lower alcohol intake subsequently reduce aggression [73~~65~~,74~~66~~]. Given the notable physical risk that alcohol poses, there is a need to address alcohol use behaviors in the Moshi area. Additionally, as research has shown that interventions are more effective when culturally adapted to specific populations [75~~67~~,76~~68~~], future alcohol-reduction-related programs and policies implemented in Moshi should encompass the specific socio-cultural nuances of the region.

In considering the social, financial, and physical impacts of alcohol use, one final note is that while broader community benefits like social unity, the upholding of traditions, and

increased government revenue were raised, participants identified few personal benefits of alcohol use. This raises the question of if alcohol truly has benefits in the community outside of historic incorporation into cultural and social events. Additional~~More~~ research exploring alcohol's impact at the personal versus community levels are thus recommended.

Limitations and Directions for Future Research

Limitations for this analysis are present primarily within the quantitative data set and the DrInC tool. First, the rate of patients who screen failed or declined survey participation was not originally collected. This has an impact on the generalizability of our quantitative data to the ED and RHC patient populations at large. Additionally, this data was collected in the midst of the COVID-19 pandemic. As such, for the safety of our Tanzanian research staff, those who presented to the ED for COVID-19-related symptoms were not approached for study participation. While it was necessary to take these safety precautions, this also limited our ability to reach the full expected patient population. In both these cases, however, because of the large sample size and well-enforced sampling strategy, we still hold our data to be representative of the patient population. For DrInC, while this tool helps measure the range of consequences one might experience from alcohol use, it does not provide information on how severe these consequences are to the individual, which limits our understanding of alcohol's true impact. Finally, several important gender-specific consequences of alcohol use arose in this analysis, highlighting the need for further exploration of how alcohol differentially impacts men and women in this region. Even with these limitations, our paper offers one of the first published assessments of community perceptions of alcohol use in sub-Saharan Africa, offering insight into how alcohol is viewed both positively and negatively by patients and community members. Our work highlights the need to consider social, financial, and cultural elements when addressing

alcohol use disorders. Particularly, we recommend that local policy and healthcare efforts acknowledge and incorporate the roles of alcohol use in future alcohol-related programming and treatment initiatives in the Moshi community.

Conclusion

Alcohol was perceived to have both positive and negative impacts on patients and their communities. Perceived harms included stigma, sexual harm, interpersonal conflict, injuries and disease, and financial instability. These harms stand in contrast to the mentioned benefits of increased social unity, upholding traditional practices, and economic growth. Further, quantitative data collected suggests that men presenting at the ED were found to experience a wider range of alcohol-related consequences than women presenting at either the RHC or ED.. Our results allow for a sharper picture of alcohol's role in this community and point to the need to incorporate the sociocultural, financial, and physical implications of alcohol use in programming and treatment related to alcohol use disorders. These findings can help identify focus areas for future programs aiming to reduce alcohol misuse in this area.

Author contributions:

Conceptualization: AMP, CAS, BTM, BM

Methodology: AMP, CAS, JRNV

Formal analysis and investigation: CAS, BTM, BM

Data Collection: MMi, JK, YS, AMP

Writing - original draft preparation: AMP, MB, KW, MM, WN

Writing - review and editing: SR, AMP, MB, KW, MM, WN, CAS

Funding acquisition: AMP, CAS

Supervision: CAS, SR, JRNV, BTM

References

1. World Health Organization (WHO). Global status report on alcohol and health 2018 [Internet]. World Health Organization (WHO); 2018 [cited 2021 Nov 21]. Available from: https://apps.who.int/iris/bitstream/handle/10665/274603/9789241565639-eng.pdf

2. Shield KD, Parry C, Rehm J. Chronic diseases and conditions related to alcohol use. Alcohol Res. 2013;35(2):155–73.

3. Rehm J, Gmel GE, Gmel G, Hasan OSM, Imtiaz S, Popova S, et al. The relationship between different dimensions of alcohol use and the burden of disease-an update. Addiction. 2017 Jun;112(6):968–1001.

4. Rehm J, Mathers C, Popova S, Thavorncharoensap M, Teerawattananon Y, Patra J. Global burden of disease and injury and economic cost attributable to alcohol use and alcohol-use disorders. Lancet. 2009 Jun 27;373(9682):2223–33.

5. Kummetat JL, Leonhard A, Manthey J, Speerforck S, Schomerus G. Understanding the Association between Alcohol Stigma and Alcohol Consumption within Europe: A Cross-Sectional Exploratory Study. Eur Addict Res. 2022 Sep 9;28(6):446–54.

6. Burke TR. The economic impact of alcohol abuse and alcoholism. Public Health Rep. 1988 Dec;103(6):564–8.

7. Thavorncharoensap M, Teerawattananon Y, Yothasamut J, Lertpitakpong C, Chaikledkaew U. The economic impact of alcohol consumption: a systematic review. Subst Abuse Treat Prev Policy. 2009 Nov 25;4:20.

8. Manthey J, Hassan SA, Carr S, Kilian C, Kuitunen-Paul S, Rehm J. What are the economic costs to society attributable to alcohol use? A systematic review and modelling study. Pharmacoeconomics. 2021 Jul;39(7):809–22.

9. Schomerus G, Lucht M, Holzinger A, Matschinger H, Carta MG, Angermeyer MC. The stigma of alcohol dependence compared with other mental disorders: a review of population studies. Alcohol Alcohol. 2011 Apr;46(2):105–12.

10. Birtel MD, Wood L, Kempa NJ. Stigma and social support in substance abuse: Implications for mental health and well-being. Psychiatry Res. 2017 Jun;252:1–8.

11. Pitpitan EV, Kalichman SC, Eaton LA, Cain D, Sikkema KJ, Skinner D, et al. Gender-based violence, alcohol use, and sexual risk among female patrons of drinking venues in Cape Town, South Africa. J Behav Med. 2013 Jun;36(3):295–304.

12. Schulkind J, Mbonye M, Watts C, Seeley J. The social context of gender-based violence, alcohol use and HIV risk among women involved in high-risk sexual behaviour and their intimate partners in Kampala, Uganda. Cult Health Sex. 2016 Jul;18(7):770–84.

13. Bonner CP, Browne FA, Ndirangu JW, Howard B, Zule WA, Speizer IS, et al. Exploring

the associations between physical and sexual gender-based violence and HIV among women who use substances in South Africa: the role of agency and alcohol. AIDS Care. 2019 Nov;31(11):1369–75.

14. Walls H, Cook S, Matzopoulos R, London L. Advancing alcohol research in low-income and middle-income countries: a global alcohol environment framework. BMJ Glob Health. 2020 Apr 16;5(4):e001958.

15. Marten R, Amul GGH, Casswell S. Alcohol: global health's blind spot. Lancet Glob Health. 2020 Mar;8(3):e329–30.

16. Ferreira-Borges C, Parry CDH, Babor TF. Harmful Use of Alcohol: A Shadow over Sub-Saharan Africa in Need of Workable Solutions. Int J Environ Res Public Health. 2017 Mar 27;14(4).

17. Lim SS, Vos T, Flaxman AD, Danaei G, Shibuya K, Adair-Rohani H, et al. A comparative risk assessment of burden of disease and injury attributable to 67 risk factors and risk factor clusters in 21 regions, 1990-2010: a systematic analysis for the Global Burden of Disease Study 2010. Lancet. 2012 Dec 15;380(9859):2224–60.

18. Murray et al. CJL. Global, regional, and national comparative risk assessment of 79 behavioural, environmental and occupational, and metabolic risks or clusters of risks, 1990-2015: a systematic analysis for the Global Burden of Disease Study 2015. Lancet. 2016 Oct 8;388(10053):1659–724.

19. Shield KD, Rehm J. Societal development and the alcohol-attributable burden of disease. Addiction. 2021 Sep;116(9):2326–38.

20. Probst C, Kilian C, Sanchez S, Lange S, Rehm J. The role of alcohol use and drinking patterns in socioeconomic inequalities in mortality: a systematic review. Lancet Public Health. 2020 Jun;5(6):e324–32.

21. Katikireddi SV, Whitley E, Lewsey J, Gray L, Leyland AH. Socioeconomic status as an effect modifier of alcohol consumption and harm: analysis of linked cohort data. Lancet Public Health. 2017 Jun;2(6):e267–76.

22. Sudhinaraset M, Wigglesworth C, Takeuchi DT. Social and Cultural Contexts of Alcohol Use: Influences in a Social-Ecological Framework. Alcohol Res. 2016;38(1):35–45.

23. Meier BJ, El-Gabri D, Friedman K, Mvungi M, Mmbaga BT, Nickenig Vissoci JR, et al. Perceptions of alcohol use among injury patients and their family members in Tanzanian society. Alcohol. 2020 Mar;83:9–15.

24. WHO. Alcohol [Fact sheet] [Internet]. World Health Organization. 2022 [cited 2023 Jan 23]. Available from: https://www.who.int/news-room/fact-sheets/detail/alcohol

25. WHO. Global Strategy to Reduce the Harmful Use of Alcohol. Geneva: World Health Organization; 2010.

26. Mitsunaga T, Larsen U. Prevalence of and risk factors associated with alcohol abuse in Moshi, northern Tanzania. J Biosoc Sci. 2008 May;40(3):379–99.

27. Francis JM, Weiss HA, Mshana G, Baisley K, Grosskurth H, Kapiga SH. The Epidemiology of Alcohol Use and Alcohol Use Disorders among Young People in Northern Tanzania. PLoS ONE. 2015 Oct 7;10(10):e0140041.

28. Mushi D, Moshiro C, Hanlon C, Francis JM, Teferra S. Missed opportunity for alcohol use disorder screening and management in primary health care facilities in northern rural Tanzania: a cross-sectional survey. Subst Abuse Treat Prev Policy. 2022 Jul 6;17(1):50.

29. Griffin SM, Karia FP, Zimmerman A, Minnig MCC, Swahn M, Makelarski J, et al. A Mixed-Methods Study: Sex Differences in Experiences of Stigma Associated With Alcoholism and Alcohol Use Disorders Among Injury Patients in Tanzania. Alcohol Clin Exp Res. 2020 Aug 9;44(8):1700–7.

30. Norris AH, Kitali AJ, Worby E. Alcohol and transactional sex: how risky is the mix? Soc Sci Med. 2009 Oct;69(8):1167–76.

31. Cooper ML. Alcohol use and risky sexual behavior among college students and youth: evaluating the evidence. J Stud Alcohol Suppl. 2002 Mar;(s14):101–17.

32. Staton CA, Vissoci JRN, Toomey N, Abdelgadir J, Chou P, Haglund M, et al. The impact of alcohol among injury patients in Moshi, Tanzania: a nested case-crossover study. BMC Public Health. 2018 Feb 21;18(1):275.

33. El-Gabri D, Nickenig Vissoci JR, Meier BJ, Mvungi M, Haglund M, Swahn M, et al. Alcohol stigma as it relates to drinking behaviors and perceptions of drink drivers: A mixed method study in Moshi, Tanzania. Alcohol. 2020 Nov;88:73–81.

34. Zimmerman A, Pesambili M, Phillips AJ, Boshe J, Mmbaga BT, Pantalon MH, et al. Cultural adaptation of a brief intervention to reduce alcohol use among injury patients in tanzania. medRxiv. 2021 Jul 22;

35. Staton CA, Zadey S, O'Leary P, Phillips A, Minja L, Swahn MH, et al. PRACT: a pragmatic randomized adaptive clinical trial protocol to investigate a culturally adapted brief negotiational intervention for alcohol use in the emergency department in Tanzania. Trials. 2022 Feb 5;23(1):120.

36. Blas E Ed. Equity, Social Determinants and Public Health Programmes. 1st ed. Geneva, Switzerland: World Health Organization; 2010.

37. Cherpitel CJ, Bond J, Ye Y, Borges G, Macdonald S, Giesbrecht N. A cross-national meta-analysis of alcohol and injury: data from the Emergency Room Collaborative Alcohol Analysis Project (ERCAAP). Addiction. 2003 Sep;98(9):1277–86.

38. WHO. Alcohol and Injury in Emergency Departments. World Health Organization. 2007;

39. Staton CA, Friedman K, Phillips AJ, Minnig MC, Sakita FM, Ngowi KM, et al.

Feasibility of a pragmatic randomized adaptive clinical trial to evaluate a brief negotiational interview for harmful and hazardous alcohol use in Moshi, Tanzania. PLoS ONE. 2023 Aug 3;18(8):e0288458. pmid:37535693

40. Isaksen, A.B., Østbye, T., Mmbaga, B.T. et al. Alcohol consumption among pregnant women in Northern Tanzania 2000–2010: a registry-based study. BMC Pregnancy Childbirth 15, 205 (2015). https://doi.org/10.1186/s12884-015-0630-0

41. Mpelo M, Kibusi SM, Moshi F, Nyundo A, Ntwenya JE, Mpondo BCT. Prevalence and Factors Influencing Alcohol Use in Pregnancy among Women Attending Antenatal Care in Dodoma Region, Tanzania: A Cross-Sectional Study. J Pregnancy. 2018 Oct 18;2018:8580318. doi: 10.1155/2018/8580318. PMID: 30420920; PMCID: PMC6211147.

4239. Pauley A, Buono M, West K, Metcalf M, Rent S, Kilasara J, et al. A Mixed-Methods Comparison of Gender Differences in Alcohol Consumption and Drinking Characteristics among Patients in Moshi, Tanzania. medRxiv. 2023 May 18;

430. Zhao D, Staton CA, He Q, Mmbaga BT, Vissoci JRN. Cross-Culture Adaptation and Psychometric Properties of the DrInC Questionnaire in Tanzanian Swahili. Front Public Health. 2018 Nov 20;6:330.

441. Miller WR, Tonigan S, Longabaugh R. THE DRINKER INVENTORY OF CONSEQUENCES (DrInC). Alcohol Res Health. 1995;

452. Kirouac M, Witkiewitz K. Revisiting the Drinker Inventory of Consequences: An extensive evaluation of psychometric properties in two alcohol clinical trials. Psychol Addict Behav. 2018;32(1):52–63.

463. Nadkarni A, Garber A, Costa S, Wood S, Kumar S, MacKinnon N, et al. Auditing the AUDIT: A systematic review of cut-off scores for the Alcohol Use Disorders Identification Test (AUDIT) in low- and middle-income countries. Drug Alcohol Depend. 2019 Sep 1;202:123–33.

474. Bohn MJ, Babor TF, Kranzler HR. The Alcohol Use Disorders Identification Test (AUDIT): validation of a screening instrument for use in medical settings. J Stud Alcohol. 1995 Jul;56(4):423–32.

485. Vissoci JRN, Hertz J, El-Gabri D, Andrade Do Nascimento JR, Pestillo De Oliveira L, Mmbaga BT, et al. Cross-Cultural Adaptation and Psychometric Properties of the AUDIT and CAGE Questionnaires in Tanzanian Swahili for a Traumatic Brain Injury Population. Alcohol Alcohol. 2018 Jan 1;53(1):112–20.

496. Saunders JB, Aasland OG, Babor TF, de la Fuente JR, Grant M. Development of the Alcohol Use Disorders Identification Test (AUDIT): WHO Collaborative Project on Early Detection of Persons with Harmful Alcohol Consumption--II. Addiction. 1993 Jun;88(6):791–804.

Field Code Changed

50. Hennink M, Kaiser BN. Sample sizes for saturation in qualitative research: A systematic review of empirical tests. Soc Sci Med. 2022 Jan;292:114523.

51. McQueen K, Namey E, Guest G. Applied Thematic Analysis. 2014, editor. SAGE Publications Inc; 2014.

52. Foley G, Timonen V. Using Grounded Theory Method to Capture and Analyze Health Care Experiences. Health Serv Res. 2015 Aug;50(4):1195-210. doi: 10.1111/1475-6773.12275. Epub 2014 Dec 18. PMID: 25523315; PMCID: PMC4545354.

53. Turner C, Astin F. Grounded theory: what makes a grounded theory study? Eur J Cardiovasc Nurs. 2021 Apr 28;20(3):285-289. Doi: 10.1093/eurjcn/zvaa034. PMID: 33772270.

54. Chapman AL, Hadfield M, Chapman CJ. Qualitative research in healthcare: an introduction to grounded theory using thematic analysis. J R Coll Physicians Edinb. 2015;45(3):201-5. doi: 10.4997/JRCPE.2015.305. PMID: 26517098.

5547. Staton CA, Zhao D, Ginalis EE, Hirshon JM, Sakita F, Swahn MH, et al. Alcohol Availability, Cost, Age of First Drink, and Its Association with At-Risk Alcohol Use in Moshi, Tanzania. Alcohol Clin Exp Res. 2020 Nov 10;44(11):2266–74.

5648. Ao TT-H, Sam N, Kiwelu I, Mahal A, Subramanian SV, Wyshak G, et al. Risk factors of alcohol problem drinking among female bar/hotel workers in Moshi, Tanzania: a multi-level analysis. AIDS Behav. 2011 Feb;15(2):330–9.

4957. El-Gabri D, Barcenas LK, Meier B, Mvungi M, Haglund M, Gerardo CJ, et al. Injury patients' perceptions of drink-driving: A qualitative assessment of drink-driving behavior in Moshi, Tanzania. PLoS ONE. 2020 May 5;15(5):e0230662.

580. Zhao D, Nickenig Vissoci JR, Mmbaga BT, Abdullah AS, Staton CA. Alcohol-Related Risky Behavior Patterns and Their Association With Alcohol Use and Perceived Alcohol Stigma in Moshi, Tanzania. J Stud Alcohol Drugs. 2020 Sep;81(5):614–23.

591. Zaleski M, Pinsky I, Laranjeira R, Ramisetty-Mikler S, Caetano R. Intimate partner violence and alcohol consumption. Rev Saude Publica. 2010 Feb;44(1):53–9.

6052. Foran HM, O'Leary KD. Alcohol and intimate partner violence: a meta-analytic review. Clin Psychol Rev. 2008 Oct;28(7):1222–34.

6153. Messersmith LJ, Halim N, Steven Mzilangwe E, Reich N, Badi L, Holmes NB, et al. Childhood trauma, gender inequitable attitudes, alcohol use and multiple sexual partners: correlates of intimate partner violence in northern tanzania. J Interpers Violence. 2021 Jan;36(1–2):820–42.

6254. Mootz JJ, Muhanguzi FK, Panko P, Mangen PO, Wainberg ML, Pinsky I, et al. Armed conflict, alcohol misuse, decision-making, and intimate partner violence among women in Northeastern Uganda: a population level study. Confl Health. 2018 Aug 6;12:37.

6355. Pauley A, Thatcher EC, Sarafian JT, Zadey S, Shayo F, Mmbaga BT, et al. Alcohol Use among Emergency Medicine Department Patients in Tanzania: A Comparative Analysis of Injury Versus Non-Injury Patients. medRxiv. 2023 May 4;

6456. Matzopoulos RG, Truen S, Bowman B, Corrigall J. The cost of harmful alcohol use in South Africa. S Afr Med J. 2014 Feb;104(2):127–32.

6557. Jørgensen MB, Pedersen J, Thygesen LC, Lau CJ, Christensen AI, Becker U, et al. Alcohol consumption and labour market participation: a prospective cohort study of transitions between work, unemployment, sickness absence, and social benefits. Eur J Epidemiol. 2019 Apr;34(4):397–407.

6658. WHO. No level of alcohol consumption is safe for our health [Internet]. World Health Organization. 2023 [cited 2023 May 11]. Available from: https://www.who.int/europe/news/item/04-01-2023-no-level-of-alcohol-consumption-is-safe-for-our-health

Field Code Changed

5967. Anderson BO, Berdzuli N, Ilbawi A, Kestel D, Kluge HP, Krech R, et al. Health and cancer risks associated with low levels of alcohol consumption. Lancet Public Health. 2023 Jan;8(1):e6–7.

680. Kalichman SC, Simbayi LC, Kaufman M, Cain D, Jooste S. Alcohol use and sexual risks for HIV/AIDS in sub-Saharan Africa: systematic review of empirical findings. Prev Sci. 2007 Jun;8(2):141–51.

691. Ferreira-Borges C, Rehm J, Dias S, Babor T, Parry CDH. The impact of alcohol consumption on African people in 2012: an analysis of burden of disease. Trop Med Int Health. 2016 Jan;21(1):52–60.

7062. Bello B, Moultrie H, Somji A, Chersich MF, Watts C, Delany-Moretlwe S. Alcohol use and sexual risk behaviour among men and women in inner-city Johannesburg, South Africa. BMC Public Health. 2017 Jul 4;17(Suppl 3):548.

7163. Zablotska IB, Gray RH, Koenig MA, Serwadda D, Nalugoda F, Kigozi G, et al. Alcohol use, intimate partner violence, sexual coercion and HIV among women aged 15-24 in Rakai, Uganda. AIDS Behav. 2009 Apr;13(2):225–33.

7264. Center for Disease Control and Prevention. Alcohol Use and Your Health [Internet]. Alcohol and Public Health. 2022 [cited 2023 May 10]. Available from: https://www.cdc.gov/alcohol/fact-sheets/alcohol-use.htm#:~:text=Over%20time%2C%20excessive%20alcohol%20use,liver%20disease%2C%20and%20digestive%20problems.&text=Cancer%20of%20the%20breast%2C%20mouth,liver%2C%20colon%2C%20and%20rectum

Field Code Changed

7365. Walton MA, Chermack ST, Shope JT, Bingham CR, Zimmerman MA, Blow FC, et al. Effects of a brief intervention for reducing violence and alcohol misuse among adolescents: a randomized controlled trial. JAMA. 2010 Aug 4;304(5):527–35.

74~~66~~. Ward CL, Mertens JR, Bresick GF, Little F, Weisner CM. Screening and Brief Intervention for Substance Misuse: Does It Reduce Aggression and HIV-Related Risk Behaviours? Alcohol Alcohol. 2015 May 1;50(3):302–9.

75~~67~~. Griner D, Smith TB. Culturally adapted mental health intervention: A meta-analytic review. Psychotherapy. 2006;43(4):531–48.

76~~68~~. Huey SJ, Polo AJ. Evidence-based psychosocial treatments for ethnic minority youth. J Clin Child Adolesc Psychol. 2008 Jan;37(1):262–301.

Supporting Information

1. Fig1
2. Fig2
3. S1 Checklist – STROBE Checklist
4. S2 Checklist – PLOS ONE Clinical Studies Checklist
5. S3 Checklist – PLOS ONE Human Subjects Research Checklist
6. S1 Questionnaire – PLOS Inclusivity in Global Research Questionnaire

Formatted: Font: (Default) Times New Roman, Font color: Black

Other

1. S1 File – Duke University Health System Institutional Review Board Approval Notice
2. S2 File – Kilimanjaro Christian Medical University College Research Ethical Clearance Certificate
3. S3 File – National Institute for Medical Research (Tanzania) Research Ethical Clearance Certificate

Formatted: Font: (Default) Times New Roman, 11 pt, Font color: Black

Formatted: Indent: Left: 0", First line: 0", Space After: 5.5 pt

February 9th, 2024

To Whom It May Concern:

We appreciate the helpful suggestions presented to us by our reviewers and thank them for the opportunity to improve our manuscript. We have incorporated the changes requested by both reviewers and our academic editor in our attached submission with our specific modifications outlined below. Our revised manuscript has been attached to this resubmission packet.

Reviewer #1:

Comment: Knowledge gap is not adequately described.
Response: *We thank the reviewer for this comment. We have edited the final paragraph of the introduction to more clearly define the knowledge gap that we aimed to address with this research. This modification now reads, 'However, literature has yet to explore the specific societal perceptions and implications of alcohol use within the Moshi community. Understanding the full scope of alcohol's impact and why its use remains endemic is essential to mitigating its effects. This knowledge, in turn, can be used to better inform and shape future alcohol-reduction interventions.'*

Comment: Study design not clearly described. The authors describe using secondary data analysis while on the other hand the authors describe the method for data collection were survey for quantitative data and in-depth interview for qualitative data. Is the author using already existing data for secondary analysis or collected the primary data during the study time?
Response: *We thank the reviewer for this clarification. We have updated the study design section of our manuscript to read, 'This study used a sequential explanatory mixed-methods approach to explore community perceptions and implications of alcohol use among KCMC's Emergency Department (ED) and Reproductive Health Center (RHC) patients in Moshi, Tanzania.' In this revision, we have removed mention of a secondary analysis as primary data was collected during the study timeline and is analyzed in this manuscript.*

Comment: Description regarding study sample/population not clear. The author does not explain the rationale of using female participants who attended RCH clinic while based on standard the pregnant woman or lactating mother were the mostly attending RCH clinic and are not allowed to take any kind of alcohol at that time due to pregnancy or lactation.
Response: *We appreciate this feedback from the reviewer and the opportunity to improve our manuscript in addressing it. We have added greater detail around our decision to enroll RHC patients under the section 'Study Setting.' This addition reads, 'the RHC…was selected because of its primarily female patient population, which facilitated a richer, gynocentric perspective on alcohol use which has been under-represented in past local research. Measuring alcohol use in pregnant or postpartum women was also important for maternal and child health given the high*

rates of alcohol use during pregnancy previously described in this setting.' The data we collected on alcohol use in pregnancy will be explored in an upcoming manuscript from our team.

Comment: The inclusion and exclusion criteria is not clearly stated.
<u>Response:</u> *We appreciate this feedback from the reviewer. We have more clearly stated our eligibility criteria in our updated section entitled 'Study Population.' This addition reads, 'Those enrolled met the following eligibility criteria: 1) fluency in Kiswahili, 2) ability to provide informed consent, 3) aged 18 or older, 4) not a prisoner, and 5) received initial care at KCMC's ED or RHC.'*

Comment: The reasons for stratified sampling is not stated.
<u>Response:</u> *We appreciate this comment from the reviewer. We have updated the "Study Design and Sampling Technique" subsection of the methods to outline the enrollment groups and rationale in accordance with our study aims. This addition reads, 'a systematic random sampling strategy was employed to acquire a representative sample of our three patient populations, male ED, female ED patients, and female RHC patients, and better describe the characteristics of each. Except for ED females, every third patient was approached for potential using the ED triage or RHC intake registries. To meet enrollment goals given that significantly fewer women seek care at the ED than men, every eligible female ED patient was approached for study participation'*

Comment: Data collection is not clear. The reason of using systematic random sampling for male participants attending at Emergency Department and Female participant attending at RCH clinic only while including every female participant attending ED not stated
<u>Response:</u> *We appreciate this feedback from the reviewer and have updated the relevant section as such. Under the same section specified in our comment above, our manuscript now reads: 'Except for ED females, every third patient was approached for potential study participation using the ED triage or RHC intake registries. For women seeking ED care, every eligible individual was approached for study participation. This was done as significantly fewer women seek care at the ED and more women were needed in this particular study to accurately determine the prevalence of AUD.'*

Comment: It is also not stated how the data analysis would be adjusted to accommodate the stratification
<u>Response:</u> *We thank the reviewer for this comment. Given that our study sought to describe alcohol-related consequences and perceptions primarily as they relate to gender and clinical unit, data stratification was not performed. Instead, descriptive statistics were used in this analysis with the intention of understanding these populations holistically.*

Comment: Data analysis for qualitative data is not clearly stated. The author describes using thematic analysis and grounded theory. Which method is used in analyzing qualitative data?
Response: *We appreciate the reviewer for bringing this gap in our methods to our attention. We have heavily revised our qualitative data analysis section to list a grounded theory methodology as well as provided a more in-depth discussion of the exact analysis methods employed. This modification can be seen in our 'Data Analysis Procedure' subsection of our 'Qualitative Data' methodology.*

Comment: The reasons for using grounded theory is not stated, also, it is not utilized either.
Response: *We appreciate this comment from the reviewer. We have made substantial changes to our qualitative data analysis section to better describe why this methodology was chosen and how it was utilized in our analysis process.*

Comment: To report if the questionnaire has been tested for internal validity not stated.
Response: *We appreciate this feedback from the reviewer. We have updated our "Data Collection and Instruments" subsection of our quantitative methods to incorporate the previous cultural adaptation and validation of the DrInC instrument in this study setting. We have additionally modified our "Data Collection and Instruments" subsection of our qualitative methods to read, 'the interview guide was piloted by research team members at KCMC to ensure interview relevancy and cultural and linguistic validity prior to the commencement of the interviews.'*

Comment: The author did not describe the demographic characteristics of the participants participating in quantitative survey.
Response: *We appreciate this feedback. We have included an additional table containing the demographic characteristics of the entire study sample in this revised manuscript version.*

Comment: Most of the quantitative data before regression analysis is not supported by statistical data
Response: *We thank the reviewer for bringing this feedback. We have added a table on study population demographics to the beginning of our quantitative results section.*

Comment: Data analysis in qualitative data is not sufficiently described. How does the end up with themes and sub themes without codes and emerging themes?
Response: *We appreciate this important question from the reviewer. We have updated our qualitative data analysis section to better describe our analysis process. The relevant part of this addition reads, 'US and Tanzanian study team members co-developed the initial codebook from the first several IDIs. This codebook was inductively and deductively generated, using broad categories from the interview guide to provide a general framework for qualitative themes along with organic generation of codes without preconceived notions from the study team in*

concordance with grounded theory methodology. The themes established within the codebook facilitated the creation of content memos for each emerging theme and sub-theme which in turn provided the basis for our qualitative findings section below. The codebook remained an evolving document as new themes emerged throughout data collection.'

Comment: The protocol is in Standard English; however, there are numerous typographical and grammatical errors.
Response: *We appreciate this feedback from the reviewer. We have revised this manuscript to address grammatical errors.*

Comment: I think the author needs to provide the recommendations to relevant authorities based on the findings of the study.
Response: *We appreciate this suggestion from the reviewer and have added a recommendation to the relevant authorities at the conclusion of our discussion section. This addition reads, 'we recommend that local policy and healthcare efforts acknowledge and incorporate the roles of alcohol use in future alcohol-related programming and treatment initiatives in the Moshi community.'*

Reviewer #2:

Comment: L114: The line states that "Factors underlying differential expectations of use will also be explored" how was this included in this study?
Response: *We thank the reviewer for bringing this incongruity to our attention. As this manuscript did not fully explore factors underlying expectations of use, we have removed this sentence from the manuscript.*

Comment: L137: Were all the participants recruited involved with alcohol use (already in use of alcohol) or anyone who falls in an interval of 3 in either EMD or RCH was involved?
Response: *We thank the reviewer for this question. To answer your initial inquiry, perceived or measured alcohol use was not considered when assessing individuals for enrollment eligibility. We have updated the "Study Population" section of our methods to clarify this point accordingly.*

Comment: L152: The line is openly stated but I suggest it to be restated as "researcher administered questionnaire was employed so as …"
Response: *We appreciate this suggestion from the reviewer. We have restated this sentence to clarify that all survey questions were delivered orally by a member of the research team.*

Comment: L163: Although there are no clear cutoff points for DrInC tool, what were the cutoff points used by previous studies that could be used as reference? OR what was the reference used to determine the cutoff points used in the current study?

Response: *We appreciate this question from the reviewer and acknowledge this gap in our manuscript. We have updated our section 'Instruments and Variable Measurements' to further explain this gap. This change now reads, '…while the DrInC tool has been validated in the study setting (DOI: 10.3389/fpubh.2018.00330.), no particular clinically relevant cut-off score for this tool exists, in part because this is among the first studies to apply this tool among Kiswahili-speaking patients, but also because ideally individuals would not experience any alcohol-related consequences. To help account for this lack of cut-off score, DrInC scores were correlated with alcohol use as is detailed further below.'*

Comment: L202: Was there any software tool used to analyze the qualitative data?
Response: *Thank you for bringing this gap in our methods section to our attention. We have added to our manuscript that 'All qualitative analysis was conducted in NVivo software version 12 and approved by the entire research team prior to the reporting of these results' to the end of our qualitative data analysis procedure subsection.*

Comment: The discussion part is good, however the perceived harm which is one of the qualitative results was more discussed as compared to perceived benefits. The comparison from different study could provide more information which can be used for future interventions of alcohol use disorders.
Response: *We appreciate this comment from the reviewer and acknowledge our discrepancy between discussed harms versus benefits. We have expanded upon our discussion of the perceived benefits of alcohol use, particularly in explaining the effects of increased social unity and financial growth for local sellers, throughout the discussion section. However, in general, IDI participants mentioned substantially more alcohol-related harms than benefits and thus were given greater space in our discussion section overall.*

Comment: Also, I would suggest adding different studies which did not concur with the results obtained from the current study so as to bring more challenges to the future researchers.
Response: *We have added greater discussion and reference to other studies that challenge our conclusions. For example, one addition reads, 'While this statement [from the WHO] and our qualitative data reports no physical benefits with alcohol use, past research has indicated that moderate alcohol intake can have positive circulatory and cardiovascular effects as well as being protective against the incidence of type 2 diabetes.'*

Academic Editor:

Comment: List the authors before the authors' affiliation.
Response: *Thank you for the feedback. The authors are listed above their respective affiliations on page 1.*

Comment: Authors' contribution has to come at the end.
<u>Response:</u> *We appreciate this revision. The authors' contributions have been moved to page 37 after the manuscript's conclusion section.*

Comment: No need of repeating the title again in page 3
<u>Response:</u> *We appreciate this revision. The title has been deleted from page 3.*

Comment: Have a heading abstract, then its sub-heading.
<u>Response:</u> *We acknowledge this request and have updated the headings and sub-headings for the abstract.*

Comment: Conclude the background of abstract with the general purpose of the study.
<u>Response:</u> *We appreciate this suggestion from the editor. We have updated the last sentence of our background within our abstract to read, 'through the voices of Kilimanjaro Christian Medical Center (KCMC) patients, this study aimed to investigate community perceptions surrounding alcohol and the impact of its use in this region.'*

Comment: In the method section have a sentence on the data collection method and tool, as well it is not clear on the data analysis method used in the study.
<u>Response:</u> *We thank the editor for this comment and have edited the methods section of our abstract accordingly. Our last sentences now read, 'The impact and perceptions of alcohol use were measured through Drinkers' Inventory of Consequences (DrInC) sores and analyzed in RStudio using descriptive proportions. IDI responses were explored through a grounded theory approach using both inductive and deductive coding methodologies in NVivo.'*

Comment: Conclusion section is not talking on the key findings obtained from the quantitative part of the study, as well in the conclusion what do you mean be ED men and ED women? These were men and women attending emergency department they cannot be called ED men and women
<u>Response:</u> *We appreciate this comment from the editor. We have edited the discussion section to include a more thorough discussion of quantitative results and how these in turn interact with our qualitative findings. Additionally, we have also removed "ED men" and "ED women" phrasing from these sections and replaced it with "men and women attending the ED".*

Comment: In line 79, the sentence has language issue, line 112 change the sentence to past tense, this is a report.
<u>Response:</u> *We appreciate these comments from the editor and both sentences have been updated accordingly. The first now reads, 'While hazardous alcohol consumption is a global issue, its use is increasing especially rapidly in low- and middle-income countries.' The latter sentence has*

been updated to '…this analysis addressed this gap by…' in accordance with your feedback.

Comment: Line 118, what do you mean by secondary analysis, did the study used secondary data? How while you have qualitative part of the study
<u>Response:</u> We thank the editor for this question. At the prompting of this comment and with further discussion, we confirm that this was not a secondary analysis; rather, we collected primary, cross-sectional data between October and May 2022 and analysis occurred following the completion of all data collection. This sentence now reads, 'this cross-sectional study used a sequential explanatory mixed-methods approach to explore community perceptions and implications of alcohol use among ED and RHC patients at KCMC between October 2021 and May 2022.'

Comment: What was the study design, mixed method is not a design rather an approach.
<u>Response:</u> We appreciate this important question and clarification. As also outlined in the comment above, we have revised our methods section to describe our study design and analytical methods more accurately, including our cross-sectional study design and sequential explanatory mixed-methods approach.

Comment: Re-arrange this section to subheadings as follows
i. Study setting-justification of the selected setting and design, which design in the mixed method was used?
ii. Study population- the inclusion and exclusion criteria
iii. Quantitative part
1. Study design
2. Sample size estimation
3. Sampling technique
4. Data collection method and instrument
5. Variables and variables measurement
6. Data analysis procedure
iv. Qualitative
1. Study design
2. Sample size estimation
3. Sampling technique
4. Data collection method and instrument
5. Data analysis procedure
This is important for clarity, in the current state the section is not clear
<u>Response:</u> We appreciate this format and organization revision from the editor. We have revised our methods section to include the outlined subheadings with the exceptions of 'study setting' and 'data collection method and instrument.' The former has been renamed as 'study overview and setting' as the initial paragraph in this section describes the broad study methods and

reasoning for our mixed-methods approach. The latter has been added as 'Data Collection Method' to improve manuscript clarity as a high amount of overlap exists between our 'instrument' and 'variable' content.

Comment: Line 137 there is RHC, this is mentioned for the first time, please clarify.
Response: *We appreciate this comment. However, RHC had previously been clarified as the Reproductive Health Center in the first sentence of our methods section, proceeding line 137. Thus, no change was made.*

Comment: How did you conduct data analysis? What analysis software did you use?
Response: *Thank you for these questions. Data analysis has been noted in our A statement noting that all quantitative data analysis was conducted in R Studio is listed on line 234. A statement has been added to the qualitative data analysis section clarifying that all qualitative analysis was conducted in NVivo software and can be found on line 292.*

Comment: Describe the study respondents' characteristics (frequency distribution table)
Response: *We appreciate this suggestion from the editor. We have included a demographics table of the entire study population in the revised version of this manuscript.*

Comment: No need for table 3 after you have attended a above
Response: *We thank the editor for this suggestion. We have removed Table 3 from the manuscript.*

Comment: No discussion for quantitative results
Response: *We appreciate this comment from the editor. We have edited the discussion section to better incorporate our quantitative findings and describe how our qualitative data contradicts or supports these data.*

Comment: Why men and women in emergency department?
Response: *We appreciate this question from the editor and have revised our methods section to make this clearer for future readers. Men and women presenting at the emergency department were enrolled in this study as previous research has suggested the higher prevalence of alcohol use among ED patients. Given that this study sought to understand the consequences of drinking itself, a patient population with above average alcohol intake was well-suited for understanding the full scope of the impacts of alcohol use. The corresponding revision can be seen in the second paragraph of our 'study overview and setting section' and is also detailed in the last response of this rebuttal letter.*

Comment: Why mixed method, what did you want to achieve by combining the approaches (quantitative and qualitative), at what point did these two approaches meet?

Response: *Thank you for this question and the opportunity for us to strengthen our manuscript in answering it. A mixed-method approach was employed to better contextualize alcohol use in describing both the quantitative prevalence of alcohol-related consequences among ED and RHC patients in Moshi, Tanzania, as well as the more nuanced community perceptions of its use. Consequently, we have added to the end of our study design section, 'this mixed-methods approach was chosen to facilitate the triangulation of data so that robust and accurate epidemiological conclusions could be drawn while also providing deeper cultural context for the complex social, emotional, and physical implications of alcohol use in this community.' Additionally, we have revised our discussion section to better explore the overlap between qualitative and quantitative data.*

Comment: Why health facility study and not community-based study, what motivated you to select health facility and ED in particular?
Response: *We appreciate this question and have updated the 'study overview and setting' section of our manuscript in line with this suggestion. To respond to this question, this update reads, 'the ED was selected as one of the two study sites to better understand risky alcohol use behaviors given the correlation between excessive alcohol use and injuries like road traffic incidents or violence that necessitate emergency care. Additionally, previous work from our team in developing alcohol-reduction interventions at KCMC's ED meant that continued research in this unit would align with programmatic goals and increase the likelihood of intervention success. The RHC, on the other hand, was chosen because of its primarily female patient population, which facilitated a richer, gynocentric perspective on alcohol use which has been under-represented in past local research. Measuring alcohol use in pregnant or postpartum women was also important for maternal and child health given the high rates of alcohol use during pregnancy previously described in this setting. As such, enrolling patients from these two clinical units allowed us to better explore the intersection of gender and hazardous drinking, the primary goal of the overarching study.'*
