# Supplementary material for: Navigating alcohol’s impact: A mixed-methods analysis of community perceptions and consequences in Northern Tanzania
Source: PLoS One. 2025 Sep 17;20(9):e0331490. doi: 10.1371/journal.pone.0331490 (PMC12443300; doi:10.1371/journal.pone.0331490)
Supplement: Attachment — Submitted filename: PONE-D-23-33073 REVEIWER COMMENTS.docx [file pone.0331490.s005.docx]

**Introduction:**

- Knowledge gap is not adequately described

**Methods:**

**Study design**

- Study design not clearly described. The author describe using secondary data analysis while on the other hand the authors describe the method for data collection were survey for quantitative data and in-depth interview for qualitative data. Is the author using already existing data for secondary analysis or collected the primary data during the study time?

**Study sample**

- Description regarding study sample/population not clear. The author does not explain the rationale of using female participants who attended RCH clinic while based on standard the pregnant woman or lactating mother were the mostly attending RCH clinic and are not allowing to take any kind of alcohol at that time due to pregnancy or lactation.
- Also the inclusion and exclusion criteria is not clearly stated
- The reasons for stratified sampling is not stated.

**Data collection and Analysis**

- Data collection is not clear. The reason of using systematic random sampling for male participants attending at Emergency Department and Female participant attending at RCH clinic only while including every female participant attending ED not stated
- It is also not stated how the data analysis would be adjusted to accommodate the stratification
- Data analysis for qualitative data is not clear stated. The author describes using thematic analysis and grounded theory. Which method is used in analyzing qualitative data?
- The reasons of using grounded theory is not stated, also, it is not utilized either.
- To report if the questionnaire have been tested for internal validity not stated

**Results**

- The author did not describe the demographic characteristics of the participants participating in quantitative survey
- Most of the quantitative data before regression analysis is not supported by statistical data
- Data analysis in qualitative data is not sufficiently described. How does the end up with themes and sub themes without codes and emerging themes?

**Generally**

- I think this is a good study with social relevance
- The protocol is in Standard English; however, there are numerous typographical and grammatical errors.
- I think the manuscript need major revision before considered for publication.
- I think the author needs to provide the recommendations to relevant authority based on the findings of the study.
