# Supplementary material for: Navigating alcohol’s impact: A mixed-methods analysis of community perceptions and consequences in Northern Tanzania
Source: PLoS One. 2025 Sep 17;20(9):e0331490. doi: 10.1371/journal.pone.0331490 (PMC12443300; doi:10.1371/journal.pone.0331490)
Supplement: Attachment — Submitted filename: Response to Reviewers.docx [file pone.0331490.s006.docx]

February 9th, 2024

To Whom It May Concern:

We appreciate the helpful suggestions presented to us by our reviewers and thank them for the opportunity to improve our manuscript. We have incorporated the changes requested by both reviewers and our academic editor in our attached submission with our specific modifications outlined below. Our revised manuscript has been attached to this resubmission packet.

Reviewer #1:

**Comment:** Knowledge gap is not adequately described.

*Response:* *We thank the reviewer for this comment. We have edited the final paragraph of the introduction to more clearly define the knowledge gap that we aimed to address with this research. This modification now reads, ‘However, literature has yet to explore the specific societal perceptions and implications of alcohol use within the Moshi community. Understanding the full scope of alcohol’s impact and why its use remains endemic is essential to mitigating its effects. This knowledge, in turn, can be used to better inform and shape future alcohol-reduction interventions.’*

**Comment:** Study design not clearly described. The authors describe using secondary data analysis while on the other hand the authors describe the method for data collection were survey for quantitative data and in-depth interview for qualitative data. Is the author using already existing data for secondary analysis or collected the primary data during the study time?

*Response:* *We thank the reviewer for this clarification. We have updated the study design section of our manuscript to read, ‘This study used a sequential explanatory mixed-methods approach to explore community perceptions and implications of alcohol use among KCMC’s Emergency Department (ED) and Reproductive Health Center (RHC) patients in Moshi, Tanzania.’ In this revision, we have removed mention of a secondary analysis as primary data was collected during the study timeline and is analyzed in this manuscript.*

**Comment:** Description regarding study sample/population not clear. The author does not explain the rationale of using female participants who attended RCH clinic while based on standard the pregnant woman or lactating mother were the mostly attending RCH clinic and are not allowed to take any kind of alcohol at that time due to pregnancy or lactation.

*Response: We appreciate this feedback from the reviewer and the opportunity to improve our manuscript in addressing it. We have added greater detail around our decision to enroll RHC patients under the section ‘Study Setting.’ This addition reads, ‘the RHC…was selected because of its primarily female patient population, which facilitated a richer, gynocentric perspective on alcohol use which has been under-represented in past local research. Measuring alcohol use in pregnant or postpartum women was also important for maternal and child health given the high rates of alcohol use during pregnancy previously described in this setting.’ The data we collected on alcohol use in pregnancy will be explored in an upcoming manuscript from our team.*

**Comment:** The inclusion and exclusion criteria is not clearly stated.

*Response: We appreciate this feedback from the reviewer. We have more clearly stated our eligibility criteria in our updated section entitled ‘Study Population.’ This addition reads, ‘Those enrolled met the following eligibility criteria: 1) fluency in Kiswahili, 2) ability to provide informed consent, 3) aged 18 or older, 4) not a prisoner, and 5) received initial care at KCMC’s ED or RHC.’*

**Comment:** The reasons for stratified sampling is not stated.

*Response: We appreciate this comment from the reviewer. We have updated the “Study Design and Sampling Technique” subsection of the methods to outline the enrollment groups and rationale in accordance with our study aims. This addition reads, ‘a systematic random sampling strategy was employed to acquire a representative sample of our three patient populations, male ED, female ED patients, and female RHC patients, and better describe the characteristics of each.* *Except for ED females, every third patient was approached for potential using the ED triage or RHC intake registries. To meet enrollment goals given that significantly fewer women seek care at the ED than men, every eligible female ED patient was approached for study participation’*

**Comment:** Data collection is not clear. The reason of using systematic random sampling for male participants attending at Emergency Department and Female participant attending at RCH clinic only while including every female participant attending ED not stated

*Response: We appreciate this feedback from the reviewer and have updated the relevant section as such. Under the same section specified in our comment above, our manuscript now reads: ‘Except for ED females, every third patient was approached for potential study participation using the ED triage or RHC intake registries. For women seeking ED care, every eligible individual was approached for study participation. This was done as significantly fewer women seek care at the ED and more women were needed in this particular study to accurately determine the prevalence of AUD.’*

**Comment:** It is also not stated how the data analysis would be adjusted to accommodate the stratification

*Response: We thank the reviewer for this comment. Given that our study sought to describe alcohol-related consequences and perceptions primarily as they relate to gender and clinical unit, data stratification was not performed. Instead, descriptive statistics were used in this analysis with the intention of understanding these populations holistically.*

**Comment:** Data analysis for qualitative data is not clearly stated. The author describes using thematic analysis and grounded theory. Which method is used in analyzing qualitative data?

*Response:* *We appreciate the reviewer for bringing this gap in our methods to our attention. We have heavily revised our qualitative data analysis section to list a grounded theory methodology as well as provided a more in-depth discussion of the exact analysis methods employed. This modification can be seen in our ‘Data Analysis Procedure’ subsection of our ‘Qualitative Data’ methodology.*

**Comment:** The reasons for using grounded theory is not stated, also, it is not utilized either.

*Response: We appreciate this comment from the reviewer. We have made substantial changes to our qualitative data analysis section to better describe why this methodology was chosen and how it was utilized in our analysis process.*

**Comment:** To report if the questionnaire has been tested for internal validity not stated.

*Response: We appreciate this feedback from the reviewer. We have updated our “Data Collection and Instruments” subsection of our quantitative methods to incorporate the previous cultural adaptation and validation of the DrInC instrument in this study setting. We have additionally modified our “Data Collection and Instruments” subsection of our qualitative methods to read, ‘the interview guide was piloted by research team members at KCMC to ensure interview relevancy and cultural and linguistic validity prior to the commencement of the interviews.’*

**Comment:** The author did not describe the demographic characteristics of the participants participating in quantitative survey.

*Response: We appreciate this feedback. We have included an additional table containing the demographic characteristics of the entire study sample in this revised manuscript version.*

**Comment:** Most of the quantitative data before regression analysis is not supported by statistical data

*Response: We thank the reviewer for bringing this feedback. We have added a table on study population demographics to the beginning of our quantitative results section.*

**Comment:** Data analysis in qualitative data is not sufficiently described. How does the end up with themes and sub themes without codes and emerging themes?

*Response: We appreciate this important question from the reviewer. We have updated our qualitative data analysis section to better describe our analysis process. The relevant part of this addition reads, ‘US and Tanzanian study team members co-developed the initial codebook from the first several IDIs. This codebook was inductively and deductively generated, using broad categories from the interview guide to provide a general framework for qualitative themes along with organic generation of codes without preconceived notions from the study team in concordance with grounded theory methodology. The themes established within the codebook facilitated the creation of content memos for each emerging theme and sub-theme which in turn provided the basis for our qualitative findings section below. The codebook remained an evolving document as new themes emerged throughout data collection.’*

**Comment:** The protocol is in Standard English; however, there are numerous typographical and grammatical errors.

*Response:* *We appreciate this feedback from the reviewer. We have revised this manuscript to address grammatical errors.*

**Comment:** I think the author needs to provide the recommendations to relevant authorities based on the findings of the study.

*Response: We appreciate this suggestion from the reviewer and have added a recommendation to the relevant authorities at the conclusion of our discussion section. This addition reads, ‘we recommend that local policy and healthcare efforts acknowledge and incorporate the roles of alcohol use in future alcohol-related programming and treatment initiatives in the Moshi community.’*

Reviewer #2:

**Comment:** L114: The line states that "Factors underlying differential expectations of use will also be explored" how was this included in this study?

*Response: We thank the reviewer for bringing this incongruity to our attention. As this manuscript did not fully explore factors underlying expectations of use, we have removed this sentence from the manuscript.*

**Comment:** L137: Were all the participants recruited involved with alcohol use (already in use of alcohol) or anyone who falls in an interval of 3 in either EMD or RCH was involved?

*Response: We thank the reviewer for this question. To answer your initial inquiry, perceived or measured alcohol use was not considered when assessing individuals for enrollment eligibility. We have updated the “Study Population” section of our methods to clarify this point accordingly.*

**Comment:** L152: The line is openly stated but I suggest it to be restated as "researcher administered questionnaire was employed so as …"

*Response: We appreciate this suggestion from the reviewer. We have restated this sentence to clarify that all survey questions were delivered orally by a member of the research team.*

**Comment:** L163: Although there are no clear cutoff points for DrInC tool, what were the cutoff points used by previous studies that could be used as reference? OR what was the reference used to determine the cutoff points used in the current study?

*Response*: *We appreciate this question from the reviewer and acknowledge this gap in our manuscript. We have updated our section ‘Instruments and Variable Measurements’ to further explain this gap. This change now reads, ‘…while the DrInC tool has been validated in the study setting (DOI: 10.3389/fpubh.2018.00330.), no particular clinically relevant cut-off score for this tool exists, in part because this is among the first studies to apply this tool among Kiswahili-speaking patients, but also because ideally individuals would not experience any alcohol-related consequences. To help account for this lack of cut-off score, DrInC scores were correlated with alcohol use as is detailed further below.’*

**Comment:** L202: Was there any software tool used to analyze the qualitative data?

*Response:* *Thank you for bringing this gap in our methods section to our attention. We have added to our manuscript that ‘All qualitative analysis was conducted in NVivo software version 12 and approved by the entire research team prior to the reporting of these results’ to the end of our qualitative data analysis procedure subsection.*

**Comment:** The discussion part is good, however the perceived harm which is one of the qualitative results was more discussed as compared to perceived benefits. The comparison from different study could provide more information which can be used for future interventions of alcohol use disorders.

*Response: We appreciate this comment from the reviewer and acknowledge our discrepancy between discussed harms versus benefits. We have expanded upon our discussion of the perceived benefits of alcohol use, particularly in explaining the effects of increased social unity and financial growth for local sellers, throughout the discussion section. However, in general, IDI participants mentioned substantially more alcohol-related harms than benefits and thus were given greater space in our discussion section overall.*

**Comment**: Also, I would suggest adding different studies which did not concur with the results obtained from the current study so as to bring more challenges to the future researchers.

*Response: We have added greater discussion and reference to other studies that challenge our conclusions. For example, one addition reads, ‘While this statement [from the WHO] and our qualitative data reports no physical benefits with alcohol use, past research has indicated that moderate alcohol intake can have positive circulatory and cardiovascular effects as well as being protective against the incidence of type 2 diabetes.’*

Academic Editor:

**Comment:** List the authors before the authors’ affiliation.

*Response:* *Thank you for the feedback. The authors are listed above their respective affiliations on page 1.*

**Comment:** Authors’ contribution has to come at the end.

*Response:* *We appreciate this revision. The authors’ contributions have been moved to page 37 after the manuscript’s conclusion section.*

**Comment:** No need of repeating the title again in page 3

*Response: We appreciate this revision. The title has been deleted from page 3.*

**Comment:** Have a heading abstract, then its sub-heading.

*Response: We acknowledge this request and have updated the headings and sub-headings for the abstract.*

**Comment:** Conclude the background of abstract with the general purpose of the study.

*Response: We appreciate this suggestion from the editor. We have updated the last sentence of our background within our abstract to read, ‘through the voices of Kilimanjaro Christian Medical Center (KCMC) patients, this study aimed to investigate community perceptions surrounding alcohol and the impact of its use in this region.’*

**Comment:** In the method section have a sentence on the data collection method and tool, as well it is not clear on the data analysis method used in the study.

*Response:* *We thank the editor for this comment and have edited the methods section of our abstract accordingly. Our last sentences now read, ‘The impact and perceptions of alcohol use were measured through Drinkers’ Inventory of Consequences (DrInC) sores and analyzed in RStudio using descriptive proportions. IDI responses were explored through a grounded theory approach using both inductive and deductive coding methodologies in NVivo.’*

**Comment:** Conclusion section is not talking on the key findings obtained from the quantitative part of the study, as well in the conclusion what do you mean be ED men and ED women? These were men and women attending emergency department they cannot be called ED men and women

*Response: We appreciate this comment from the editor. We have edited the discussion section to include a more thorough discussion of quantitative results and how these in turn interact with our qualitative findings. Additionally, we have also removed “ED men” and “ED women” phrasing from these sections and replaced it with “men and women attending the ED”.*

**Comment:** In line 79, the sentence has language issue, line 112 change the sentence to past tense, this is a report.

*Response:* *We appreciate these comments from the editor and both sentences have been updated accordingly. The first now reads, ‘While hazardous alcohol consumption is a global issue, its use is increasing especially rapidly in low- and middle-income countries.’ The latter sentence has been updated to ‘…this analysis addressed this gap by…’ in accordance with your feedback.*

**Comment:** Line 118, what do you mean by secondary analysis, did the study used secondary data? How while you have qualitative part of the study

*Response: We thank the editor for this question. At the prompting of this comment and with further discussion, we confirm that this was not a secondary analysis; rather, we collected primary, cross-sectional data between October and May 2022 and analysis occurred following the completion of all data collection. This sentence now reads, ‘this cross-sectional study used a sequential explanatory mixed-methods approach to explore community perceptions and implications of alcohol use among ED and RHC patients at KCMC between October 2021 and May 2022.’*

**Comment:** What was the study design, mixed method is not a design rather an approach.

*Response:* *We appreciate this important question and clarification. As also outlined in the comment above, we have revised our methods section to describe our study design and analytical methods more accurately, including our cross-sectional study design and sequential explanatory mixed-methods approach.*

**Comment:** Re-arrange this section to subheadings as follows
i. Study setting-justification of the selected setting and design, which design in the mixed method was used?
ii. Study population- the inclusion and exclusion criteria
iii. Quantitative part
1. Study design
2. Sample size estimation
3. Sampling technique
4. Data collection method and instrument
5. Variables and variables measurement
6. Data analysis procedure
iv. Qualitative
1. Study design
2. Sample size estimation
3. Sampling technique
4. Data collection method and instrument
5. Data analysis procedure
This is important for clarity, in the current state the section is not clear

*Response: We appreciate this format and organization revision from the editor. We have revised our methods section to include the outlined subheadings with the exceptions of ‘study setting’ and ‘data collection method and instrument.’ The former has been renamed as ‘study overview and setting’ as the initial paragraph in this section describes the broad study methods and reasoning for our mixed-methods approach. The latter has been added as ‘Data Collection Method’ to improve manuscript clarity as a high amount of overlap exists between our ‘instrument’ and ‘variable’ content.*

**Comment:** Line 137 there is RHC, this is mentioned for the first time, please clarify.

*Response:* *We appreciate this comment. However, RHC had previously been clarified as the Reproductive Health Center in the first sentence of our methods section, proceeding line 137. Thus, no change was made.*

**Comment:** How did you conduct data analysis? What analysis software did you use?

*Response:* *Thank you for these questions. Data analysis has been noted in our A statement noting that all quantitative data analysis was conducted in R Studio is listed on line 234. A statement has been added to the qualitative data analysis section clarifying that all qualitative analysis was conducted in NVivo software and can be found on line 292.*

**Comment:** Describe the study respondents’ characteristics (frequency distribution table)

*Response*: *We appreciate this suggestion from the editor. We have included a demographics table of the entire study population in the revised version of this manuscript.*

**Comment:** No need for table 3 after you have attended a above

*Response: We thank the editor for this suggestion. We have removed Table 3 from the manuscript.*

**Comment:** No discussion for quantitative results

*Response: We appreciate this comment from the editor. We have edited the discussion section to better incorporate our quantitative findings and describe how our qualitative data contradicts or supports these data.*

**Comment:** Why men and women in emergency department?

*Response:* *We appreciate this question from the editor and have revised our methods section to make this clearer for future readers. Men and women presenting at the emergency department were enrolled in this study as previous research has suggested the higher prevalence of alcohol use among ED patients. Given that this study sought to understand the consequences of drinking itself, a patient population with above average alcohol intake was well-suited for understanding the full scope of the impacts of alcohol use. The corresponding revision can be seen in the second paragraph of our ‘study overview and setting section’ and is also detailed in the last response of this rebuttal letter.*

**Comment:** Why mixed method, what did you want to achieve by combining the approaches (quantitative and qualitative), at what point did these two approaches meet?

*Response:* *Thank you for this question and the opportunity for us to strengthen our manuscript in answering it. A mixed-method approach was employed to better contextualize alcohol use in describing both the quantitative prevalence of alcohol-related consequences among ED and RHC patients in Moshi, Tanzania, as well as the more nuanced community perceptions of its use. Consequently, we have added to the end of our study design section, ‘this mixed-methods approach was chosen to facilitate the triangulation of data so that robust and accurate epidemiological conclusions could be drawn while also providing deeper cultural context for the complex social, emotional, and physical implications of alcohol use in this community.’ Additionally, we have revised our discussion section to better explore the overlap between qualitative and quantitative data.*

**Comment:** Why health facility study and not community-based study, what motivated you to select health facility and ED in particular?

*Response:* *We appreciate this question and have updated the ‘study overview and setting’ section of our manuscript in line with this suggestion. To respond to this question, this update reads, ‘the ED was selected as one of the two study sites to better understand risky alcohol use behaviors given the correlation between excessive alcohol use and injuries like road traffic incidents or violence that necessitate emergency care. Additionally, previous work from our team in developing alcohol-reduction interventions at KCMC’s ED meant that continued research in this unit would align with programmatic goals and increase the likelihood of intervention success.* *The RHC, on the other hand, was chosen because of its primarily female patient population, which facilitated a richer, gynocentric perspective on alcohol use which has been under-represented in past local research. Measuring alcohol use in pregnant or postpartum women was also important for maternal and child health given the high rates of alcohol use during pregnancy previously described in this setting. As such, enrolling patients from these two clinical units allowed us to better explore the intersection of gender and hazardous drinking, the primary goal of the overarching study.’*
