# Supplementary material for: Navigating alcohol’s impact: A mixed-methods analysis of community perceptions and consequences in Northern Tanzania
Source: PLoS One. 2025 Sep 17;20(9):e0331490. doi: 10.1371/journal.pone.0331490 (PMC12443300; doi:10.1371/journal.pone.0331490)
Supplement: Attachment — Submitted filename: Response to Reviewers - Impact.docx [file pone.0331490.s009.docx]

December 8th, 2024

To whom it may concern,

We very much appreciate the helpful suggestions and methodological questions raised by Reviewer 1 and thank them for the opportunity to further improve our manuscript. We have incorporated the requested changes. Our specific modifications are outlined below, and our revised manuscript has been attached to this resubmission packet.

**INTRODUCTION**

**Comment:** Knowledge gap is not adequately described. The author did not describe what is a real problem under the study

*Response: We thank the reviewer for this comment. We have edited the last paragraph of the introduction to better define and outline our knowledge gap for our readers. This change reads: “However, an exploration of the specific societal views of alcohol use within Moshi or the perceived harms and benefits that underlie continued consumption has yet to be conducted. Understanding the full scope of alcohol’s impact and role within the community is essential in combatting excess intake as it allows local leaders and researchers to address the root cause of dependence.” We hope this clarifies the concerns regarding the knowledge gap we aim to fill in this manuscript.*

**Comment:** The author does not indicate any justification of undertaking this study.

*Response: We thank the reviewer for this suggestion and the opportunity to improve our manuscript in addressing it. We have strengthened the justification portion of our introduction, which can be found in the last paragraph of the introduction. Our justification now reads, “Understanding the full scope of alcohol’s impact within the community is essential in combatting excess intake as it allows local leaders and researchers to address the root cause of dependence. This knowledge can, in turn, better inform future alcohol-reduction policies, interventions, and research initiatives, allowing them to be more targeted, socioculturally appropriate, and effective in decreasing alcohol use and related harm in Moshi [36]. This process of development can also serve as a model for population-specific harm reduction interventions in other settings.”*

**METHODS**

*Quantitative*

**Comment:** Study design is not clearly described as the author does not illustrate if the study is hospital based or community based because in many areas within the manuscript the authors indicate both **patients and community members** eg in L# 647. Therefore, the authors should be consistent so as to make it easy understood by readers.

*Response: We appreciate this feedback from the reviewer. We have modified our “Study Overview” section of the methods to state that this was a hospital-based study. Additionally, we have updated our terminology throughout the introduction, methods, and quantitative results section to describe participants as patients rather than community members. However, the use of the word ‘community’ has been retained within the qualitative portion of our manuscript as patients directly describe the impact of alcohol on their community; for example, IDI #19 states that his “community strongly hates women who drinks alcohol” or IDI #2 says “people who drink Gongo and Dadii are really stigmatized in the community.” In these quotes and framing these statements, we use the term community. We hope these edits and discussion clarify any misunderstandings regarding the study setting and population.*

**Comment:** Description regarding study sample/population not clear. In a quantitative part the author stated that “the study team planned to enroll more women the men” but the author does not give any justification on that?

*Response: We thank the reviewer for raising this concern and appreciate the opportunity to clarify. We acknowledge that our initial explanation of the study sample size estimation may have been unclear. In the revised manuscript, we provide the following justification:*

*“Quantitative sample size was calculated based on the difference in average Alcohol Use Disorder Identification Test (AUDIT) scores between our RHC and ED patient populations, as the prevalence of alcohol use disorder (AUD) was a primary point of interest in the broader study [42]. Based on existing literature and input from local research team members, we hypothesized the prevalence of AUD to be 10% among RHC women, 15% among ED women, and 30% among ED men. Thus, at 80% power and 90% confidence, a sample size of 587 participants was calculated to sufficiently estimate the prevalence of risky drinking across the three subgroups. Given that the expected difference in prevalence was narrower between the two female groups than between male and female groups, the study team initially prioritized enrolling more women to ensure sufficient statistical power to detect differences between the two female populations.*

*Preliminary data analysis revealed that AUD prevalence was much higher than anticipated (40% in ED women and 45% in ED men). Recalculations of sample size based on these updated prevalence estimates revealed 1,200 patients would be needed to determine differences in proportions between these two groups. While the study timeline and funding could not accommodate the newly estimated sample size of 1200 participants, with IRB approval, the study’s target sample size was increased to enroll as many male and female participants as feasible within the study timeline.”*

*We hope that this revision provides a clearer justification for our enrollment strategy, specifically addressing the reasoning for prioritizing female enrollment based on narrower expected differences.*

**Comment:** Description regarding instruments used in the data collection is not clear

*Response: We appreciate this comment from the reviewer. We have added an additional paragraph to our “Instruments and Variable Measurements” section to provide greater clarity on our instruments. This addition reads: “The quantitative surveys administered to participants consisted of three major components: (1) collection of basic demographic data, such as participants’ age and gender; (2) assessment of typical alcohol use practices, including frequency and quantity of consumption as well as typical expenditure on alcohol; and (3) administration of validated instruments, specifically the AUDIT and DrInC tools. The Alcohol Use Disorders Identification Test (AUDIT) is a 10-item screening tool developed by the World Health Organization to identify hazardous, harmful, and dependent patterns of alcohol use. Scores range from 0 to 40, with higher scores indicating more hazardous consumption and scores of 8 or greater signaling clinically significant risk for adverse alcohol-related health effects. The AUDIT has undergone extensive psychometric and clinical validation across diverse populations and has been specifically adapted and validated in Tanzania through its KiSwahili translation, ensuring cultural and linguistic appropriateness for this context.” Please let us know if further description is needed.*

**Comment:** Also the author stated that “DrInC tools has been validated” the author does not indicate the type of validity employed in the validation process and unfortunately the DrInC items in description differ with the item in the referred table. Additionally the author does not tell anything about the reliability of the tool used.

*Response: We thank the reviewer for this feedback regarding the DrInC tool and appreciate the opportunity to improve our manuscript in answering these concerns. We have provided greater explanation of the tools reliability and validity which can be found under the ‘Instruments and Variable Measurements’ section of the Methods. This addition reads, “Prior confirmatory factor analysis conducted in the study setting demonstrated the construct validity of the tool, confirming its five-domain structure as reflective of distinct alcohol-related consequences [43]. Reliability was also supported by high internal consistency, with Cronbach’s alpha exceeding 0.8 across all domains [43]. These findings validate the tool’s suitability amongst KiSwahili-speaking participants, ensuring cultural and linguistic relevance in this context [43].”*

**Comment:** The author does not describe clearly a cutoff points for DrInC tool, what were the cutoff points used by previous studies that could be used as reference? OR what was the reference used by the authors to determine the cutoff points used in the current study?

*Response: We appreciate this question from the reviewer and have clarified the issue regarding cutoff points for the DrInC tool. This description reads, "Although the DrInC tool quantifies a range of alcohol-related consequences, it does not measure their severity or frequency, and no universally accepted clinical cutoff score exists. Designed to capture the broad spectrum of alcohol-related issues, the tool has not been associated with specific cutoff scores in previous studies [43–45]. Consistent with this approach, we did not apply a cutoff score in our study, and instead, DrInC scores were analyzed in relation to alcohol use patterns as described below.”*

**Comment:** Also the author describe AUDIT as tool in the analysis part but does not included in the instrument used description.

*Response: We appreciate this important comment and have provided greater description of the AUDIT survey instrument. The manuscript has been updated accordingly within the “Instruments and Variable Measurement” section, which has been outlined in a previous response.*

*Qualitative*

**Comment:** Description regarding sample size estimation in a qualitative part is not clear and sufficient.

*Response: Thank you for this feedback. We have thoroughly revised this section to better describe and justify our sample size estimation. This addition reads, “Of the 676 individuals initially enrolled in this study, all 655 patients who had fully completed quantitative surveys were eligible for IDI enrollment. The study team aimed to enroll 20 IDI participants — ten male participants from the ED group and ten female participants, five each from the ED and RHC groups — or until data saturation was reached, whichever occurred first. This approach was chosen to provide equal representation from both genders, while also prioritizing thematic saturation as the primary determinant for concluding data collection.*

*This original estimated sample size of 20 participants was informed by commonly accepted recommendations in qualitative research, which suggest 15-20 interviews as a general guideline for reaching saturation in relatively homogenous groups [50]. Saturation was also explicitly defined as the point at which no new themes emerged after three consecutive interviews for each subgroup (ED men, ED women, and RHC women). To account for the iterative nature of qualitative research, the team regularly monitored emerging themes throughout data collection and concluded after 19 interviews when saturation was reached across all subgroups. This approach helped balance initial pragmatic estimates of sample size with the flexibility needed to capture all relevant themes.”*

**Comment:** The author stated that “the study team aimed to enroll 20 ID participants or until data saturation has been met” which one does the author rely on? How sure the author anticipated that the saturation will be reached within 20 participants?

*Response: We appreciate this question from the reviewer and have edited this section accordingly, as can also be seen in our response above. While we initially targeted 20 participants based on qualitative research guidelines, the study did not rely solely on this initial sample size but prioritized thematic saturation as the primary determinant for concluding data collection. Using a pragmatic initial estimate of 20 participants while retaining some degree of flexibility within our sampe size estimate allowed us to ensure that all relevant themes were captured and provided a more robust criterion for ensuring data adequacy. This sentiment can be seen in the revisions detailed above.*

**Comment:** Also, which criteria does the author use to select 10 men out of 114 and 10 women, five from each group? so as to avoid bias?

*Response: We appreciate this comment from the reviewer and have better emphasized within our ‘Sample Size Estimation’ section that this was done to ensure equal gender representation in IDI responses. This change is detailed in our reply above.*

**Comment:** Does the author take 19 patients from all 676 enrolled patients or 19 from 655 patients who completed survey in quantitative part?

*Response: We thank the reviewer for this important question. We have updated the ‘Sample Size Estimation’ and Sampling Technique’ portions of our qualitative methods to clarify that only participants with fully completed quantitative surveys were eligible for participation in IDI.*

**Comment:** Which criteria the author used to decide to select 20 participants from 655? Which sampling technique the author utilized to select 20 participants out of 655 participants?

*Response: We thank the reviewer for bringing these gap in our manuscript to our attention and have revised the “Sampling Technique” section of our methods to better detail our purposive sampling strategy. This revision reads: “A purposive sampling strategy was employed to ensure representation across key demographic and experiential characteristics from the 655 participants who completed the initial quantitative survey. Participants were purposively selected to ensure diversity across key factors, including age, marital status, education level, occupation, tribe, and religion. Additionally, IDI participants were chosen to reflect varying personal experiences with and perspectives on alcohol use, from patients who only reported neutral or positive past experiences with alcohol, used to drink but are now abstinent, have a close friend or relative that drinks heavily, or suffered significant negative consequences from alcohol. Preliminary quantitative findings also informed participant selection to better explore notable patterns identified in the data. For example, early quantitative analysis indicated that divorced or widowed women had above-average alcohol intake, prompting the inclusion of a recently divorced woman with high alcohol consumption to explore this trend in greater depth.*

*To ensure a representative sample and minimize potential bias, the characteristics of IDI participants were reviewed monthly by the study lead. Any imbalances or gaps in representation were addressed through adjustments to subsequent participant selection. This iterative process ensured that diverse demographic and experiential perspectives were captured while aligning with the study’s overarching focus.” We hope that this revision satisfactorily addresses the concerns raised by the reviewer.*

**Comment:** The author does not indicate the sample population used in the pilot of the interview guide.

*Response: We appreciate this comment from the reviewer and have clarified our phrasing on pilot testing to better illustrate the sample population used. This change now reads, “The interview guide was piloted with the study lead and three KiSwahili-speaking research team members, all of whom were local healthcare professionals and researchers familiar with both the target population and healthcare context. Pilot testing was done to ensure its cultural and linguistic validity prior to the commencement of the interviews.” Please let us know if any further revisions are needed.*

**Comment:** The author does not illustrate if the participants was informed about the reimbursement provided to them before been interviewed?

*Response: We appreciate this feedback on our manuscript and the opportunity to improve our work in answering it. We have revised this section to clarify that participants were informed prior to participation in the study that they would be reimbursed for travel to and from KCMC. This clarification reads: “Participants were informed prior to participation that they would be given a small stipend (5000 TSH, equivalent to approximately $2 USD) to reimburse their travel expenses.” We hope this edit resolves any concerns regarding interviewee reimbursement.*

**Comment:** The author does not give the sufficient rationale of using grounded theory in the data analysis. Why does the author not consider using thematic analysis as it was before rather than using a grounded theory?

*Response: We appreciate these questions and the opportunity to clarify our qualitative analysis methodology. Unlike thematic analysis, which organizes data around predefined themes, grounded theory was selected because this topic is under-researched in the local context, necessitating a theory-building approach to derive new insights directly from the data. We have now elaborated on this rationale in the revised manuscript. Our revised ‘Data Analysis Procedure’ now reads: “A grounded theory approach was chosen because this topic—societal perceptions and implications of alcohol use in Moshi—remains poorly understood in the local context, with no prior research to draw from. Unlike thematic analysis, which is often used to organize and interpret pre-existing frameworks or themes, grounded theory is designed to generate new, theory-driven insights directly from the data itself. This approach allowed us to iteratively learn from participants’ responses and develop a holistic understanding of alcohol use perceptions specific to this setting, rooted in the data rather than external frameworks.*

*US and Tanzanian study team members co-developed the initial codebook from the first several IDIs. This codebook was inductively and deductively generated, using broad categories from the interview guide as an initial framework while also enabling organic generation of codes without preconceived notions, in alignment with grounded theory methodology.” We hope that this revision provides better rationalization for our grounded theory approach.*

**RESULTS**

**Comment:** The author just interpret only the demographic characteristics of the participants

*Response: Thank you for your comment. We have expanded the results section to more thoroughly describe statistical analyses of alcohol-related behaviors and their consequences, including DrInC and AUDIT scores, and their associations across populations.*

**Comment:** L# 288-291 the author does not indicate any statistical data that support the given interpretation

*Response: We thank the reviewer for bringing this discrepancy to our attention. We have removed this sentence from the results section. Instead, we reference these data points (what was previously found in line 288-291) in the discussion section as this has been previously published by our team:* [*doi.org/10.1371/journal.pgph.0002009*](http://doi.org/10.1371/journal.pgph.0002009)*.*

**Comment:** Also the alcohol-related consequences or impact as it was the aim of the study should be presented or showed in this quantitative part before taken it into qualitative part but the author does not provide any results regarding those alcohol impact.

*Response: We appreciate the opportunity to clarify this important point. In line with this feedback, we have added a dedicated section on alcohol-related consequences within the quantitative results for greater clarity. Here, we now present AUDIT and DrInC scores and subscores in better detail and assess their variation across patient groups.*

*This revision reads: “Descriptive analyses revealed significant variation in alcohol consumption and related consequences across populations. ED men had the highest average AUDIT scores (mean = 6.76, SD = 8.16), followed by ED women (mean = 3.07, SD = 4.76) and RHC women (mean = 1.86, SD = 3.46). Similarly, DrInC scores showed that ED men experienced the most alcohol-related consequences (mean = 16.4, SD = 19.6), followed by ED women (mean = 9.11, SD = 13.1) and RHC women (mean = 5.47, SD = 9.33) (Table 3). DrInC subscores for physical, interpersonal, social responsibility, and impulse control consequences followed the same trend, where ED men followed by ED women and RHC women faced a higher burden of alcohol-related physical, interpersonal, intrapersonal, impulse control, and social responsibility harms (Table 3, Figure 1). Of note, ED women showed the largest variability in DrInC scores in several subcategories, including physical and intrapersonal consequences (Figure 1).” Finally, we have added greater interpretation and contextualization of DrInC scores within the discussion section. We hope that these changes address the reviewer’s concerns.*

**Comment:** The author does not provide the descriptive analysis regarding AUDIT and DrInC score before taking it to find its association

*Response: We thank the reviewer for bringing this gap in our results to our attention. We now have summarized trends in AUDIT scores across groups in paragraph two of the quantitative results section before delving into the linear relationship in paragraph three. This revision can be seen in our response above. Additionally, we have incorporated average AUDIT scores into Table 3 for clarity. We once again thank the reviewer for this comment.*

**Comment:** Additionally in the previous reviewer comments the author responded that “descriptive statistics were used in this analysis with the intention of understanding these populations holistically” but there is no any descriptive analysis performed rather the author performed linear regression to establish the association between AUDIT and DrInC score

*Response: We thank the reviewer for raising this concern, however, we respectfully clarify that descriptive statistics were indeed employed to summarize population characteristics and alcohol-related outcomes. These analyses are presented in Table 2 (demographics) and Table 3 (AUDIT and DrInC score summaries), along with visual representation in Figure 1 (boxplots of DrInC subscores). While our descriptive analyses provide a holistic understanding of the study population, the linear regression analyses in Figure 1 specifically explore the association between AUDIT and DrInC scores, addressing a separate analytic objective.*

**Comment:** L#295-297 the information described is not been found in the table 2 which the author referring to

*Response: We thank the reviewer for bringing this error to our attention. We have updated this section to note that this data can be found in Table 3 instead of Table 2.*

**Comment:** Why does the author include the P-values in the demographic information table?

*Response: We appreciate this thought from the reviewer and have removed P-Values from our ‘Table 2: Study Population Demographics’ table.*

**Comment:** The author does not indicates those variables analyzed so as to get these P-value? and which statistical test did he/she use? The author should consider to separate the P-Value data from the demographic information table and to have its own table that will shows those variable analyzed

*Response: We thank the reviewer for bringing these gaps in Table 2 to our attention and have removed the P-Values as noted above. Table 2 now consists entirely of descriptive statistics as described in our quantitative results.*

**Comment:** L# 288-289 the author stated that “Men attending ED found to spend the most of their money on alcohol per week” but unfortunately the author does not shows the data that justify this interpreted results since in demographic information table it indicates the personal income per month and there is no any information indicates how much money spent on alcohol by all three groups of participants so as to reach to that conclusion the author ended with

*Response: We appreciate the reviewer for bringing this gap to our attention and acknowledge the error in our last submission. We have removed mention of men’s spending on alcohol use from the results section, and instead now only discuss it in the discussion section, as this data comes from a previously published paper from our team:* [*doi.org/10.1371/journal.pgph.0002009*](http://doi.org/10.1371/journal.pgph.0002009)*.*

**Comment:** L# 205-206 the stated that “Overall and category specific DrInC scores for the three patient populations were assessed through descriptive frequencies and proportions” but on the results section there is no any descriptive frequency and proportion illustrated there. Therefore the authors should revise a section of DATA ANALYSIS Procedure in quantitative so as to tally with the results provided

*Response: We thank the reviewer for bringing this inconsistency to our attention. We have revised this sentence: “AUDIT and DrInC scores for the three patient populations were assessed by calculating means and standard deviations for overall and category-specific scores.” We hope that this change addresses the concerns raised by this reviewer.*

**Comment:** The author does not provide any interpretation regarding the information provided in figure 1, figure 2 and in table 3

*Response: We thank the reviewer for providing this suggestion so that we can better improve our manuscript. We have expanded the quantitative results section to better describe Figures 1 and 2 and tables 2 and 3. The revised descriptions for Figures 1 and Table 2 and 3 have been previously provided in this revision letter. The expanded interpretation of Figure 2 is as follows: “Linear regression analyses demonstrated a significant linear association between AUDIT and DrInC scores across all groups (Figure 2). The association was strongest for ED women (R² = 0.76, p < 0.001), reflecting that higher alcohol use strongly correlates with more alcohol-related consequences in this group. The association was slightly weaker for ED men (R² = 0.65, p < 0.001) and RHC women (R² = 0.53, p < 0.001), indicating population-specific differences in the impact of alcohol consumption (Figure 2).” These results have also been better contextualized within the discussion section, as seen in our replies below. Please let us know if further interpretation is needed.*

**Comment:** The authors does not describe which criteria they use to select 19 participants out of 655 participants for qualitative.

*Response: We appreciate this feedback on our manuscript. To address this feedback, we have heavily revised our ‘Sampling Technique’ and ‘Data Collection and Instruments’ sections of our qualitative methods to better describe the criteria for selecting IDI participants.*

**Comment:** The author did not describe the demographic characteristics of the participants participating in qualitative as not all participants from the quantitative were included on the qualitative. Therefore the author should provide the table showed the characteristic of the participants involved in qualitative part.

*Response: We appreciate this feedback from the reviewer. We have provided an additional table within the revised version of this manuscript that displays interviewee demographics for the 19 IDI participants included within this study as requested.*

**DISCUSSION**

**Comment:** The discussion part is good, however the perceived harm which is one of the qualitative results was more discussed than perceived benefits. The author should make comparison from different study conducted in the same area of the study which can be used for future interventions.

*Response: We appreciate this comment from the reviewer and have added a stronger discussion around alcohol’s perceived benefits to our discussion section. This revision reads: “In this analysis, we found that participants saw both positive and negative impacts of alcohol use at the individual, interpersonal, and community levels. While alcohol was viewed as contributing to harms like interpersonal violence, stigma, and financial instability, it was also recognized for its role in fostering social unity and maintaining traditional customs within Moshi. Participants frequently describe alcohol as a facilitator of unity, enabling individuals to come together for celebrations, rituals, or even conflict resolution. These findings align with global literature on the duality of alcohol's social effects, where its consumption is not only a public health challenge but also an entrenched part of social and cultural life.” This helps to underlie some of our previous revisions highlighting positive economic benefits of alcohol use, which read: “Other important economic-based findings from this analysis are the impacts at broader community and government levels, the effects of which were noted by participants to be overall positive. IDI participants believed that high rates of consumption were advantageous for sellers of alcohol and rising national excise taxes on alcohol products contributed to increasing revenue for the government of Tanzania.”*

*However, overall, we agree that alcohol’s perceived harms are discussed more than alcohol’s perceived benefits. This arises from (1) the quantitative focus on harms, given that the DrInC tool focuses solely on negative consequences, and (2) IDI participants mentioned substantially more alcohol-related harms than benefits. As such, alcohol-related harms were given greater space in our discussion section to reflect our findings. While we believe it may be misleading to have benefits and harms equally represented in our discussion section, we hope these revisions help provide a more balanced perspective.*

**Comment:** L# 531-533 describe that the current study use the existing data. Does the author use the existing data or they conduct survey? The author should revise this part to make it clear

*Response: Thank you for this feedback. We have revised this sentence for clarity. This sentence now reads: “Our paper extends existing research in Moshi through prospectively collected surveys and IDIs to offer a more nuanced understanding of alcohol’s social, economic, and physical implications among patients, their families, and community members.”*

**Comment:** The author should discuss the findings revealed in the study in relation to other research conducted on the same area

*Response: We thank the reviewer for this suggestion and have added several additional, important research works also conducted in Tanzania to our discussion. For example, the work by McCoy et al. helps to better contextualize our economic findings, as we say: “McCoy et al. note that women who produce alcohol in Tanzania have limited control over any earned income and are placed at greater risk of sexual assault or unsafe sex practices, indicating that the benefits for sellers may exist primarily for men.” We hope these revisions address the concerns raised by the reviewer.*

**Comment:** The author does not discuss the findings for quantitative data, they discuss only the qualitative findings

*Response: We thank the reviewer for this comment and have gone back through our discussion section to ensure our quantitative results have been adequately highlighted in each section. Examples of how this is highlighted are:*

(1) *“For example, our quantitative analysis revealed that men responded affirmatively to experiencing approximately three different types of interpersonal consequences related to alcohol use via the DrInC scale, compared to 0.39 for female RHC patients and one for female ED patients.” in paragraph two of our discussion,*
(2) *“Our analysis highlights a gendered component to alcohol-related financial consequences, with men scoring over twice as high as women in the social responsibility category of drink DrInC, which encapsulates questions like ‘I have missed days or work’ and ‘I have spent too much’ in paragraph four of our discussion, and*
(3) *“. Male ED patients were seen to experience a wider range of physical consequences than women presenting at the ED and RHC as illustrated by our DrInC physical harm scores. Men answered positively to experiencing 2.54 alcohol-related physical harms compared to 0.60 from female RHC patients and 1.39 female ED patients. IDI data provided more information on the types of physical harm thought to be suffered by this patient population” in paragraph 6 of our discussion.*

*We hope that these incorporations are sufficient for the review, and ask to let us know if further changes are recommended.*

**Comment:** Also the author includes the recommendation within the discussion section. It will be more wise, presentable and easily pinpointed by the respective authorities for action if the author separate the recommendation on the separate from discussion with its sub- title before limitation of the study section and it should tally with the results obtained from the study

*Response: We appreciate this feedback from the reviewer and have added a separate ‘Recommendations’ section accordingly. This recommendation section now reads: “Based on the data we present, we recommend four key areas for future directions research, intervention, and policy efforts: 1) First, researchers should further explore the perceived social benefits associated with alcohol use, particularly its role in fostering unity and maintaining unity within Moshi. This knowledge will facilitate a better understanding of the drivers of its current widespread use, even among harmful consequences. 2) To build upon further research and maximize program efficacy, alcohol reduction interventions should consider the distinct sociocultural contexts of alcohol use within this region and implement these nuanced considerations within intervention study designs. This will allow researchers and health professionals to create more culturally sensitive programs that can more effectively reduce local alcohol misuse.3) Finally, the micro- and macroeconomic implications of alcohol use, including perceived financial benefits and economic losses, should be analyzed to inform evidence-based alcohol-related policies in Tanzania.” We again that the reviewer for this suggestions and opportunity to strengthen our manuscript.*

**CONCLUSION**

**Comment:** The author should conclude in relation with the revealed results

*Response: We have reworked the conclusion section of this manuscript to better summarize our results, in line with the reviewer’s helpful suggestion. Out conclusion now reads: “Alcohol use in Moshi, Tanzania, was perceived to have both positive and negative impacts on patients and their communities. Social harms such as stigma, interpersonal conflict, and intimate partner violence were contrasted by perceived benefits like fostering social unity and upholding cultural traditions. Economic harms, including excessive household spending and decreased income, outweighed the broader financial benefits of alcohol sales and tax revenue. Physically, alcohol use was associated with injuries, risky sexual behaviors, and chronic health conditions, with no reported physical benefits. These findings offer critical insight into alcohol’s dual role in Moshi as both a source of harm and facilitator of social cohesion, underscoring the need to integrate sociocultural, financial, and physical considerations into alcohol-reduction programming and policy efforts. Future research should examine alcohol’s broader implications to guide effective, evidence-based policies and programs.”*

**GENERAL COMMENTS**

**Comment:** The manuscript is in Standard English; however, there are numerous typographical and grammatical errors.

*Response: Thank you for this feedback. We have edited the manuscript in its entirety with this resubmission to address typographical and grammatical errors.*

**Comment:** The author should justify the works so as to make the work clean with crisp edges.

*Response: We thank the reviewer for this recommendation and have gone through our discussion section again to better justify our work and support our mixed methods approach. These changes include the addition of the following sentences: (1) “We used quantitative methods (DrInC scale) to identify the broad scope and frequency of alcohol-related consequences, while qualitative interviews added depth, revealing perceptions of these consequences and their sociocultural context. This mixed-methods approach allows for a more comprehensive understanding of alcohol’s impacts” and (2) “By integrating quantitative and qualitative methods, this study captures both the measurable impacts of alcohol and the perceptions that shape its role in Moshi’s sociocultural and economic fabric” to the beginning and end of our discussion section in concordance with your recommendation.*

**Comment:** The author need to make a results for a quantitative clear and understood

*Response: We have done a thorough revision of the quantitative results section as well as bolstered their contextualization within our discussion section. These changes have been explicitly outlined in our responses above. We hope that these changes sufficiently address the concerns raised.*

**Comment:** The result from quantitative and qualitative should tally with the title of the study as the current findings based more on the perception of alcohol consumption and live behind the impact of alcohol impacts which was supposed to be revealed and elaborated on the quantitative part so as to provide justification for proceeding with qualitative part of the study

*Response: We thank the reviewer for this feedback. We have reworked our manuscript title to reflect this sentiment better. Our manuscript is now entitled: “Navigating Alcohol’s Impact: A Mixed-Methods Analysis of Community Perceptions and Consequences in Northern Tanzania.” Furthermore, we hope that the extensive changes made in our results and discussion sections better triangulate our quantitative and qualitative results. We ask the reviewer to please let us know if further changes are needed.*
