# Supplementary material for: Navigating alcohol’s impact: A mixed-methods analysis of community perceptions and consequences in Northern Tanzania
Source: PLoS One. 2025 Sep 17;20(9):e0331490. doi: 10.1371/journal.pone.0331490 (PMC12443300; doi:10.1371/journal.pone.0331490)
Supplement: Attachment — Submitted filename: Response to Reviewers - Impact - 7.14.docx [file pone.0331490.s012.docx]

July 14, 2025

To whom it may concern,

We appreciate the helpful suggestions and questions raised by Reviewer 1 and thank them for the opportunity to further improve our manuscript. We have incorporated the requested changes. Our specific modifications are outlined below, and our revised manuscript has been attached to this resubmission packet.

**INTRODUCTION**

**Comment:** LN# 72-75 The author should rephrase the sentence to make it clear

*Response: We thank the reviewer for this suggestion. We have revised Lines 71-74 to more clearly state how alcohol is embedded in social and cultural norms in Tanzania. The revised version now states: “Although alcohol use is associated with significant harms, it remains deeply embedded in cultural and social practices around the world [22]. In Tanzania, for example, alcohol is often normalized from a young age and used as a form of social currency—practices that contribute to higher levels of consumption [23].”*

**Comment:** LN# 91-94. The author addresses this comment insufficiently since the authors report their previous work it could be better if they could indicate the prevalence of alcohol consumption and prevalence of alcohol-related injuries in the area.

*Response: We appreciate this comment from the reviewer. We have revised Lines 92-97 to include an additional sentence that reports the prevalence of alcohol-related injuries at KCMC. The revised sentence states, “Among injury patients presenting to the Kilimanjaro Christian Medical Centre Emergency Department, nearly 30% had been consuming alcohol when the injury occurred.” Additionally, we have revised Lines 89-90 to specify that the prevalence of alcohol use is 2.5 times higher than in nearby regions and one of the highest reported rates of alcohol intake in Tanzania. We hope these revisions sufficiently address the reviewer’s concerns.*

**METHODS:**

**Comment:** Description regarding instruments used in the data collection is not clear

*Response: We appreciate this comment from the reviewer. We have revised the quantitative instruments and variable measurements section to more clearly describe the data collection tools used in our study. Specifically, we have clarified the format, items, and scoring of the Drinker Inventory of Consequences (DrInC) assessment in Lines 222-228 stating, “The Drinker Inventory of Consequences (DrInC) was used to quantitatively assess the consequences of alcohol use among participants. This 50-item, yes/no questionnaire has been previously cross-culturally adapted and clinically validated at Kilimanjaro Christian Medical Centre (KCMC) [51]. The tool evaluates alcohol-related consequences across five domains: Physical, Social Responsibility, Interpersonal, Intrapersonal, and Impulse Control (Table 1) [52,53]. Of the 50 items, 45 assess negative consequences and are positively scored, while 5 serve as control items, reflecting perceived benefits of alcohol use (e.g., enjoying the taste or drinking without problems), and are reverse scored. Responses were collected for both the past three months and across the participant’s lifetime.”*

**Comment:** The author stated that&quot; the survey tool used consist 50 items from

5 domains and you refer to table 1 but in the actual table there are 45 items. where are those 5 items? or which item are you referring to?. Also, the author should consider rechecking LN# 211-211 which table are you referring? Since the actual Table 1 there are 45 items and not 50 as the author started.

*Response: We appreciate this important question from the reviewer. We have revised Lines 222-286 to more clearly articulate the structure, subcategories, and scoring of the DrInC tool. Specifically, the five subcategories of the DrInC tool contain 45 items that measure adverse consequences related to alcohol use, which are depicted in Table 1. An additional five control items that reflect perceived alcohol-related benefits are included to comprise 50 total items. All 50 items were assessed in the study and scored appropriately (45 items positively scored, 5 items reverse scored).*

**Comment:** The author does not indicate the sample population used in the pilot of the interview guide

*Response: We thank the reviewer for this comment. We have revised Lines 324-328 to more clearly describe our sample population for the qualitative interview pilot. Notably, we have added the clinical and research backgrounds of the KCMC research assistants who the guide was piloted with and their role in ensuring the cultural and linguistic validity of the interview guide.*

**Comment:** What was the sample used in the pilot? is the same sample included in the actual study? it would be better if you indicated the sample used in the pilot. are they the same patients used in the study? or? are they from the same clinical departments?

*Response: We thank the reviewer for this thoughtful comment regarding the pilot sample. We have revised the manuscript to explicitly describe the individuals involved in piloting the interview guide. This addition reads: “The interview guide was piloted with the study lead and three Kiswahili-speaking research team members from the research department at Kilimanjaro Christian Medical Centre (KCMC). These team members, who were not part of the final study sample, had professional backgrounds in nursing, medicine, and qualitative research, and were familiar with the target population and healthcare setting. Their feedback informed refinements to ensure the cultural and linguistic validity of the guide prior to initiating interviews with participants. Pilot interviews were conducted solely for refinement purposes and were not included in the study dataset.”*

*As noted, the pilot was conducted with three Kiswahili-speaking research team members at KCMC with professional experience in nursing, medicine, and qualitative research. Although these individuals were not part of the study sample, their familiarity with the local healthcare context and the target population allowed them to provide meaningful feedback on both linguistic clarity and cultural relevance. The pilot interviews were used exclusively to refine the guide and were not included in the study data or analysis. While we acknowledge that piloting directly with members of the target population can offer additional advantages, this approach was not conducted with this study. Nonetheless, we are confident that our piloting process appropriately ensured the guide’s validity and suitability for use in this context.*

GENERAL COMMENTS:

**Comment:** The authors address all comments adequately except for the remaining few which were inadequately addressed and it would be wise if the author could address them to make the manuscripts better.

*Response: We thank the reviewer for this comment and the opportunity to enhance our work. We hope our revisions have adequately addressed the reviewer’s comments.*

**Comment:** The author should justify the work to make the work clean with crisp edges. This is about the alignment of the work and not justification of work as the author tries to address this comment.

*Response: We appreciate this comment from the reviewer. We have made two changes to the manuscript to address the reviewer’s suggestions. First, we have revised Lines 106-107 of the introduction section to more clearly justify the work and the knowledge gap. The revised text states, “The justification of this work lies in its potential ability to inform future alcohol-reduction policies, interventions, and research initiatives, allowing them to be more targeted, socioculturally appropriate, and effective in decreasing alcohol use and related harm in Moshi.” This statement helps to emphasize the broader alignment of this work towards alcohol-reduction strategies and improving health outcomes.*

*Second, we have included an additional sentence in Lines 726-728 of the discussion section to articulate how our research expands and aligns with existing alcohol research in the region. The revised text states, “Building on existing alcohol research in the region, insights from this analysis can help guide future culturally relevant research and alcohol reduction efforts in Moshi, and inform similar interventions in other communities.” We hope these revisions adequately address the reviewer’s concerns.*
