# Supplementary material for: Navigating alcohol’s impact: A mixed-methods analysis of community perceptions and consequences in Northern Tanzania
Source: PLoS One. 2025 Sep 17;20(9):e0331490. doi: 10.1371/journal.pone.0331490 (PMC12443300; doi:10.1371/journal.pone.0331490)
Supplement: Attachment — Submitted filename: Reviewer Comments JAN-2025.docx [file pone.0331490.s011.docx]

**Reviewer Comments**

| **S/N** | **SECTION** | **DESCRIPTION** |
| --- | --- | --- |
| 1 | Introduction | - LN# 72-75 The author should rephrase the sentence to make it clear - LN# 91-94. The author addresses this comment insufficiently since the authors report their previous work it could be better if they could indicate the prevalence of alcohol consumption and prevalence of alcohol-related injuries in the area |
|  |  |  |
| 2 | Methods | **QUANTITATIVE**  Instruments and Variable Measurements   - Description regarding instruments used in the data collection is not clear - The author stated that" the survey tool used consist 50 items from 5 domains and you refer to table 1 but in the actual table there are 45 items. where are those 5 items? or which item are you referring to?. Also, the author should consider rechecking LN# 211-211 which table are you referring? Since the actual Table 1 there are 45 items and not 50 as the author started. |
| 3 |  | **QUALITATIVE**  Sample Size Estimation   - Data Collection and Instruments   - The author does not indicate the sample population used in the pilot of the interview guide   - What was the sample used in the pilot? is the same sample included in the actual study? it would be better if you indicated the sample used in the pilot. are they the same patients used in the study? or? are they from the same clinical departments? |
|  |  |  |
| 4 | General comments | - The authors address all comments adequately except for the remaining few which were inadequately addressed and it would be wise if the author could address them to make the manuscripts better - The author should justify the work to make the work clean with crisp edges. This is about the alignment of the work and not justification of work as the author tries to address this comment. |
|  |  |  |
