# Supplementary material for: Navigating alcohol’s impact: A mixed-methods analysis of community perceptions and consequences in Northern Tanzania
Source: PLoS One. 2025 Sep 17;20(9):e0331490. doi: 10.1371/journal.pone.0331490 (PMC12443300; doi:10.1371/journal.pone.0331490)
Supplement: Attachment — Submitted filename: Reviewer Comments August_2025.docx [file pone.0331490.s014.docx]

**Reviewer Comments**

| **S/N** | **DESCRIPTION** |
| --- | --- |
|  | The author address all the comments therefore editorial team can proceed with the publication process |
|  |  |
