# Supplementary material for: Navigating alcohol’s impact: A mixed-methods analysis of community perceptions and consequences in Northern Tanzania
Source: PLoS One. 2025 Sep 17;20(9):e0331490. doi: 10.1371/journal.pone.0331490 (PMC12443300; doi:10.1371/journal.pone.0331490)
Supplement: Attachment — Submitted filename: Reviewer Comments.pdf [file pone.0331490.s008.pdf]

Reviewer Comments

S/N	SECTION	DESCRIPTION
1	Introduction	• Knowledge gap is not adequately described. The author did not describe what is a real problem under the study<br>• The author does not indicate any justification of undertaking this study
2	Methods	**QUANTITATIVE**<br>Study design<br>• Study design is not clearly described as the author does not illustrate if the study is hospital based or community based because in many areas within the manuscript the authors indicate both **patients and community members** eg in L# 647. Therefore, the authors should be consistence so as to make it easy understood by readers<br>Description regarding study sample/population not clear<br>• In a quantitative part the author stated that "the study team planned to enroll more women the men but the author does not give any justification on that?<br><br>Instruments and Variable Measurements<br>• Description regarding instruments used in the data collection is not clear<br>• Also the author stated that "DrInC tools has been validated" the author does not indicate the type of validity employed in validation process and unfortunately the DrInC items in description differ with the item in the referred table<br>• Additionally the author does not tell anything about the reliability of the tool used<br>• The author does not describe clearly a cutoff points for DrInC tool, what were the cutoff points used by previous studies that could be used as reference? OR what was the reference used by the authors to determine the cutoff points used in the current study?<br>• Also the author describe AUDIT as tool in the analysis part but does not included in the instrument used description
		**QUALITATIVE**<br>Sample Size Estimation<br>• Description regarding sample size estimation in a qualitative part is not clear and sufficient as:<br>o The author stated that "the study team aimed to enroll 20 ID participants or until data saturation has been met" which one does the author rely on? How sure the author anticipated that the saturation will be reached within 20 participants?<br>o Which criteria the author used to decide to select 20 participants from 655?

		- Also, which criteria does the author use to select 10 men out 0f 114 and 10 women, five from each group? so as to avoid bias? - Sampling technique - Does the author take 19 patients from all 676 enrolled patients or 19 from 655 patients who completed survey in quantitative part? - Which sampling technique the author utilized to select 20 participants out of 655 participants? - Data Collection and Instruments - The author does not indicate the sample population used in the pilot of the interview guide - The author does not illustrate if the participants was informed about the reimbursement provided to them before been interviewed? - Data Analysis Procedure - The author does not give the sufficient rationale of using grounded theory in the data analysis - Why the author does not consider to use thematic analysis as it was before rather than using a grounded theory?
3	Results	**QUANTITATIVE DATA** - The author just interpret only the demographic characteristics of the participants - L# 288-291 the author does not indicate any statistical data that support the given interpretation - Also the alcohol-related consequences or impact as it was the aim of the study should be presented or showed in this quantitative part before taken it into qualitative part but the author does not provide any results regarding those alcohol impact - The author does not provide the descriptive analysis regarding AUDIT and DrInC score before taking it to find its assscieation - Additionally in the previous reviewer comments the author responded that “descriptive statistics were used in this analysis with the intention of understanding these populations holistically” but there is no any descriptive analysis performed rather the author performed linear regression to establish the association between AUDIT and DrInC score - L#295-297 the information described is not been found in the table 2 which the author referring to - Why does the author include the P-values in the demographic information table? - The author does not indicates those variables analyzed so as to get these P-value? and which statistical test did he/she use?

		• The author should consider to separate the P-Value data from the demographic information table and to have its own table that will shows those variable analyzed<br>• L# 288-289 the author stated that "Men attending ED found to spend the most of their money on alcohol per week" but unfortunately the author does not shows the data that justify this interpreted results since in demographic information table it indicates the personal income per month and there is no any information indicates how much money spent on alcohol by all three groups of participants so as to reach to that conclusion the author ended with<br>• L# 205-206 the stated that "Overall and category specific DrInC scores for the three patient populations were assessed through descriptive frequencies and proportions" but on the results section there is no any descriptive frequency and proportion illustrated there. Therefore the authors should revise a section of DATA ANALYSIS Procedure in quantitative so as to tally with the results provided<br>• The author does not provide any interpretation regarding the information provided in figure 1, figure 2 and in table 3
		**QUALITATIVE**<br>• The authors does not describe which criteria they use to select 19 participants out of 655 participants for qualitative<br>• The author did not describe the demographic characteristics of the participants participating in qualitative as not all participants from the quantitative were included on the qualitative. Therefore the author should provide the table showed the characteristic of the participants involved in qualitative part
4	Discussion	• The discussion part is good, however the perceived harm which is one of the qualitative results was more discussed than perceived benefits. The author should make comparison from different study conducted in the same area of the study which can be used for future interventions<br>• L# 531-533 describe that the current study use the existing data. Does the author use the existing data or they conduct survey? The author should revise this part to make it clear<br>• The author should discuss the findings revealed in the study in relation to other research conducted on the same area<br>• The author does not discuss the findings for quantitative data, they discuss only the qualitative findings<br>• Also the author includes the recommendation within the discussion section. It will be more wise, presentable and easily pinpointed by the respective authorities for action if the author separate the recommendation on the separate from discussion with its sub- title

		before limitation of the study section and it should tally with the results obtained from the study
5	Conclusion	• The author should conclude in relation with the revealed results
6	General comments	• The manuscript is in Standard English; however, there are numerous typographical and grammatical errors<br>• The author should justify the works so as to make the work clean with crisp edges<br>• The author need to make a results for a quantitative clear and understood<br>• The result from quantitative and qualitative should tally with the title of the study as the current findings based more on the perception of alcohol consumption and live behind the impact of alcohol impacts which was supposed to be revealed and elaborated on the quantitative part so as to provide justification for proceeding with qualitative part of the study
